# The epigenetic processes of meiosis in male mice are broadly affected by the widely used herbicide atrazine

**DOI:** 10.1186/s12864-015-2095-y

**Published:** 2015-10-30

**Authors:** Aurore Gely-Pernot, Chunxiang Hao, Emmanuelle Becker, Igor Stuparevic, Christine Kervarrec, Frédéric Chalmel, Michael Primig, Bernard Jégou, Fatima Smagulova

**Affiliations:** Inserm U1085 IRSET, 263 Avenue du Général Leclerc, 35042 Rennes, France; EHESP, Avenue du Professeur Léon-Bernard, 35043 Rennes, France; Present address: University of Zagreb, Faculty of Food Technology and Biotechnology, 10000 Zagreb, Croatia

**Keywords:** Meiosis, Double-strand breaks, Gene-Chip, ChIP-Seq, H3K4me3, Atrazine

## Abstract

**Background:**

Environmental factors such as pesticides can cause phenotypic changes in various organisms, including mammals. We studied the effects of the widely used herbicide atrazine (ATZ) on meiosis, a key step of gametogenesis, in male mice.

**Methods:**

Gene expression pattern was analysed by Gene–Chip array. Genome-wide mapping of H3K4me3 marks distribution was done by ChIP-sequencing of testis tissue using Illumina technologies. RT-qPCR was used to validate differentially expressed genes or differential peaks.

**Results:**

We demonstrate that exposure to ATZ reduces testosterone levels and the number of spermatozoa in the epididymis and delays meiosis. Using Gene-Chip and ChIP-Seq analysis of H3K4me3 marks, we found that a broad range of cellular functions, including GTPase activity, mitochondrial function and steroid-hormone metabolism, are affected by ATZ. Furthermore, treated mice display enriched histone H3K4me3 marks in regions of strong recombination (double-strand break sites), within very large genes and reduced marks in the pseudoautosomal region of X chromosome.

**Conclusions:**

Our data demonstrate that atrazine exposure interferes with normal meiosis, which affects spermatozoa production.

**Electronic supplementary material:**

The online version of this article (doi:10.1186/s12864-015-2095-y) contains supplementary material, which is available to authorized users.

## Background

Environmental factors can alter DNA methylation and epigenetic modifications in the germline [51, 90]. We studied the effects of the widely used herbicide atrazine (ATZ) on meiosis. In many countries, ATZ is the most common contaminant detected in rivers [19, 72, 92]. ATZ residues are even detected in soil and aqueous environments in countries where it has been banned [59, 102, 103]. Low levels of ATZ metabolites in pregnant women are associated with low birth weight [16, 61]. In mammals, ATZ is metabolized by several groups of xenobiotic-metabolizing enzymes. The metabolism of ATZ interferes with oxidative phosphorylation and cytochrome P450 function, resulting in decreased oxygen consumption [10, 14, 39, 47]. ATZ is known to alter reproductive processes in rodents [96, 101], reptiles [18], birds [104], goats [81], amphibians [28–30] and fish [63, 69]. ATZ targets many tissues, including the testes [67, 68, 101], ovaries [8, 17, 41, 52], brain [5, 7], liver [25, 36, 38, 78]. The effects of ATZ on reproduction have been extensively studied; however, the effect of ATZ on meiosis has not been elucidated.

Meiosis is the division of germ cells and is essential for the production of haploid gametes. In most organisms, homologous recombination (HR) is important for the proper segregation of chromosomes during meiosis. HR predominantly occurs in discrete areas of the genome known as recombination hotspots. Histone H3 trimethylation at lysine 4 (H3K4me3) by PRDM9 methyltransferase is indispensable for the proper formation of double-strand breaks (DSBs) throughout the genome, except in the pseudoautosomal region (PAR) of X chromosome [12]. Germ cells lacking PRDM9 are unable to repair DSBs at the pachytene stage of gametogenesis, and knock-out mice are infertile in both sexes [27, 53]. It has also been shown that environmental pollutants affect HR both in male and female. The first study illustrating the effects of environmental exposure on meiosis was the bisphenol A (BPA) treatment study, in which the authors showed that embryonic exposure to BPA causes meiotic anomalies, which lead to aneuploidy in adult female mice [34]. Importantly, meiotic effects in animals were induced by environmentally relevant doses of BPA [34]. BPA exposure of adult rat delays meiosis initiation and induces accumulation of meiotic DNA DSBs in the late meiotic stage [48]. In nematodes BPA exposure also results in impaired chromosome synapsis and disruption of meiotic double-strand break repair (DSBR) progression of C. elegans [4].

We hypothesized that ATZ may also alter meiosis because it reduces sperm number and changes testis morphology in animals [1, 101]. Here, we studied the mechanisms by which ATZ affects meiosis in mice. We showed that exposure to ATZ increases testosterone levels and reduces the number of spermatozoa in epididymis, causing a delay of meiosis. Using Gene-Chip and ChIP-Seq analysis of H3K4me3 marks, we found that a wide range of cellular functions, including GTPase activity, mitochondrial function and steroid-hormone metabolism, are affected by ATZ. Furthermore, histone H3K4me3 marks are enriched in treated mice in regions of strong recombination (DSB sites) within very large genes and reduced at the PAR region of the X chromosome. We therefore propose that ATZ disrupts normal meiosis.

## Results

### Experimental design

We treated adult male mice with ATZ at a concentration of 100 mg/l in water for three weeks and followed with two weeks without treatment to cover one full round of spermatogenesis (35 days). Given that mice drink approximately 5 ml of water per day, we estimate that the average intake of ATZ was approximately 25 mg/kg/day. This dose is relatively low compared to that used previously [1, 67, 96]. We chose this dose of ATZ because doses higher than 50 mg/kg/day are known to change the weight of internal organs and are considered as toxic [101]. To study the effect of ATZ on the first wave of spermatogenesis, we treated newborn animals from postnatal day 1 through day 20 dpp by administering ATZ suspended in oil to the lactating dams at a dose of 25 mg/kg/day.

### Low levels of atrazine do not affect testicular morphology and cell viability

After treatment, the mice were euthanized, and reproductive organs were dissected. We measured the organs and body weights of treated and control animals. We found that the body weight and reproductive organs (testes, epididymis and seminal vesicles) were not affected at this dose (data not shown). To evaluate morphological changes in seminiferous tubules caused by ATZ, we analyzed paraffin-embedded sections stained with hematoxylin and eosin and compared matching stages from treated and control animals. The morphological analysis did not reveal any significant changes in either the seminiferous tubules or the epididymis (Additional file 1: Figure S1 (A-D)). However, we could not exclude small changes in the proportion of cells, which are difficult to distinguish by this method. We verified cell populations by staining with propidium iodide (DNA-staining agent) and sorting by FACS and we comcluded that cell populations were not significantly affected by this treatment (Additional file 1: Figure S2 (A-E)). To examine whether the number of germ or Sertoli cells had changed, we immunostained testicular tissue with antibodies specific for undifferentiated spermatogonial stem cells (PLZF) and Sertoli cells (GATA1) (Additional file 1: Figure S3 (A-B)). We found that treatment did not significantly alter the number or proportion of those cell types (Additional file 1: Figure S3C). To evaluate the rate of apoptosis, we performed TUNEL assays using the *In Situ* Cell-Death Detection kit. We did not detect significant changes in the rate of apoptosis in adult ATZ-treated mice compared to control (Additional file 1: Figure S3D). Thus, treatment with a relatively low dose of atrazine does not affect organ weight or the cell populations within seminiferous tubules.

### Decreased sperm count and serum testosterone levels in ATZ-treated mice

**Fig. 1:**
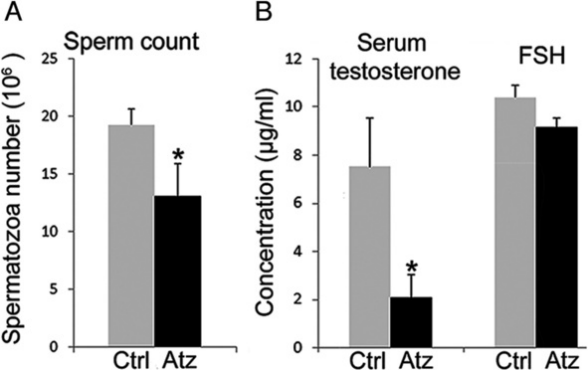
Low-dose ATZ treatment decreases spermatozoa numbers and testosterone levels. **a** The number of spermatozoa in the epididymis decreased. **b** The amount of testosterone in the serum decreased; *n* = 10/group; values are means +/−SD, while FSH was not significantly affected (*p < 0.05, *n* = 10/group, values are means +/−SD)

One essential metric of reproductive state is the number of spermatozoa per epididymis. To evaluate the effect of ATZ, we counted spermatozoa in the epididymis of treated and control mice. We found that ATZ-treated mice had a significantly lower sperm count (68 %) than untreated animals (*p < 0.05; *n* = 10, Fig. 1a). To assess endocrine function after treatment, we measured serum levels of testosterone and follicle-stimulating hormone (FSH). We found that serum testosterone was 3.4 times lower in treated animals (*p < 0.05, *n* = 10), while the level of FSH was not significantly affected (*p* = 0.06, *n* = 10, Fig. 1b). Our data show that a relatively low dose of atrazine decreases sperm counts and serum testosterone levels.

### Analysis of the synaptonemal complex reveals delayed meiosis

**Fig. 2:**
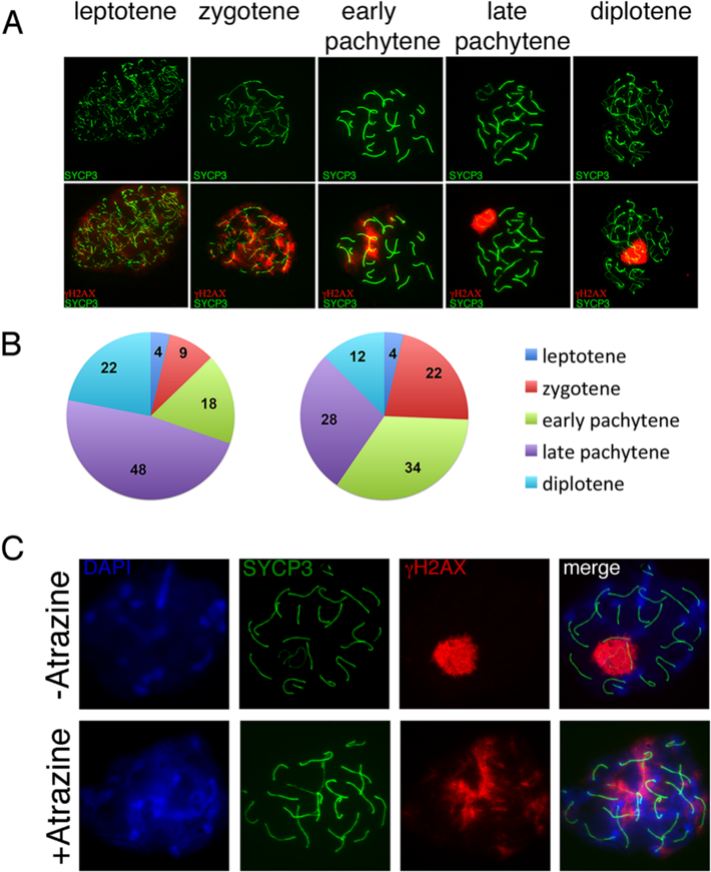
Atrazine causes delayed meiosis in mice. **a** γH2AX staining changes during meiotic prophase I substages. **b** The distribution of meiotic substages in control (left panel) and ATZ-treated (right panel) mice; 600 cells from control and treated mice were classified according to γH2AX and SYCP3 staining. Note that the number of spermatocytes in the zygotene and early pachytene stages is increased in ATZ-treated animals, while the number of spermatocytes in the late stages decreased. **c** Surface spreads from control and ATZ-treated testis stained with anti-γH2AX (red) and anti-SYCP3 (green) antibodies. In control mice, the majority of meiotic cells have γH2AX marks at the sex bodies only. In ATZ-treated mice, the majority of meiotic cells have γH2AX marks at many chromosomes

We hypothesized that a decrease in sperm count might reflect defects in meiotic progression or spermiogenesis. We thus asked whether ATZ negatively affects the meiotic stages of spermatogenesis. To analyze meiotic progression in treated mice, we performed surface spreads from testis tissue and analyzed synaptonemal-complex (SC) formation. The SC is formed exclusively during prophase 1 of meiosis, and its major role is to bring the chromosomes together [32]. Prophase I is subdivided into several sub-stages (leptotene, zygotene, pachytene, diplotene and diakinesis). We immunostained the surface cell spreads with antibodies against SYCP3 (marker of synaptonemal complex proteins) and γH2AX (stains chromatin at DSB sites) and then classified cells into meiotic substages (Fig. 2a) as previously described [50, 62], (see Materials and Methods for more details). During the leptotene phase, there is an increase in the number and intensity of γH2AX-positive domains throughout the nucleus. In the zygotene stages, the number of γH2AX domains and the intensity of γH2AX staining decreased, as the staining was concentrated over the chromatin. At the early pachytene stage, γH2AX had almost disappeared from the chromatin of the autosomes, but there were still some visible traces of marks on autosomes, and marks appeared to cover the sex chromosomes. At the late pachytene stage, γH2AX was detected only at the sex chromosomes. The proportions of prophase I substages detected in control mice were similar to those detected by other authors [13, 20]. However, we found that the number of early stage spermatocytes increased in ATZ-treated animals (2.4 times the number observed in the zygotene and 1.9 times that observed in the early pachytene stages), while the late stages decreased (1.8 times fewer than observed in the diplotene stages) (Pearson’s Chi-squared test, *p* < 2.2e-16) (Fig. 2b). In control mice, the largest fraction of spermatocytes was in the late pachytene stage, where γH2AX markers were detectable only in sex chromosomes (Fig. 2c). In ATZ-treated animals, the majority of spermatocytes showed patterns of γH2AX staining typical of early stages (Fig. 2c). We performed quantitative analysis of early pachytene cell fractions in control and treated mice, and we detected three major defects in ATZ-treated mice: (1) incomplete synapsing of one or two chromosomes, which was often detected together with the formation of branched chromosome structures; (2) sex-body defects (sex chromosomes with very long chromosomal axes (Additional file 1: Figure S4A). We found that the number of cells with incomplete synapses increased 3.1 times in treated cells compared to untreated cells, the number of cells with sex-chromosome defects increased 3.6 times (Additional file 1: Figure S4B).

The performed analysis of immunostaining pattern of γH2AX marks helped us to evaluate the meiotic progression, however, we do not exclude the fact that our observation is not perfect due to the possibility that meiosis in some cells is deregulated and that the stages could not be defined exactly. Our analysis is designed to observe the general view of meiotic progress. We complete this finding by analyzing meiotic progress during the first wave of spermatogenesis (see section below).

Taken together, our data show that ATZ has an effect on meiotic progression and increases the number of synapsing defects.

### ATZ treatment delays meiotic progression due to persistence of DSBs

**Fig. 3:**
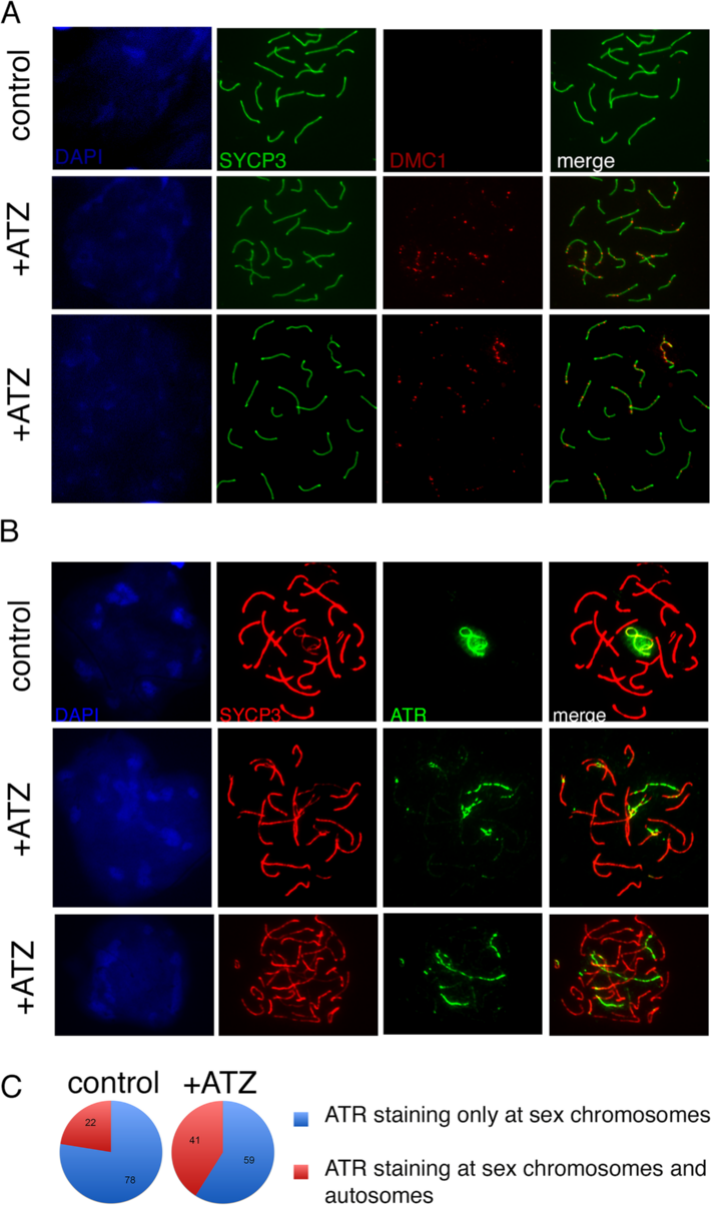
The proportions of DMC1- and ATR-positive cells increase upon treatment. **a** Spreads from control and ATZ-treated mice were stained with anti-DMC1 (red) and anti-SYCP3 (green). In control mice, the DSBs are repaired at this stage, such that no DMC1 foci are detectable. In ATZ-treated samples (middle and low panels), several DMC1 foci are detectable at the pachytene stage. **b** Spreads stained with anti-ATR (green) and anti-SYCP3 (red). In control mice, the ATR signal is detected in sex bodies only, while ATR staining is detected at several chromosomes in ATZ-treated mice. **c** Quantitative analysis of ATR staining. The spreads were costained with ATR and SYCP3 antibodies and classified according to pattern: sex body only (shown in blue) and sex body with staining of autosomal chromosomes (shown in red)

We next asked whether the increase in early stage meiotic cells occurred due to the persistence of DSBs breaks. Chromosomes undergo DSB formation at the leptotene-zygotene stage. At this stage, a maximal number of DSBs can be detected by staining surface spreads with an antibody against DMC1, which is a DNA-binding protein of DSBs. We found that the number of cells with DMC1 foci increased with ATZ treatment (Fig. 3a). Most of the cells positive for DMC1 in ATZ-treated animals had foci at the sex chromosomes. The increase in the number of DMC1-positive cells with treatment could be the result of inefficient repair of DSBs in sex chromosomes.

To further examine synapsing efficiency in ATZ-treated mice, we stained chromosomes with an antibody against ATR (ataxia telangiectasia and Rad3-related), a marker of asynapsed chromosomes and silenced chromatin [97] (Fig. 3b). ATR acts stage-specifically to regulate multiple aspects of mammalian meiotic silencing [75]. If asynapsis persists at the pachytene stage, ATR leads to the induction of repressive post-translational modifications and irreversible gene silencing over many megabases [75]. To analyze the role of ATR in ATZ-treated mice, we counted the number of cells that showed staining of ATR at the sex body only or that showed simultaneous ATR staining of both XY bivalents and autosomes. In ATZ-treated mice, there was 1.8 times more ATR staining of autosomes (Fig. 3c). Consistent with previous data on the role of ATR in chromatin silencing [75], our experiments showed that ATZ treatment increases the number of chromosomes with incomplete synapsing.

### Delayed meiotic progression and increased apoptosis in young prepubertal animals

**Fig. 4:**
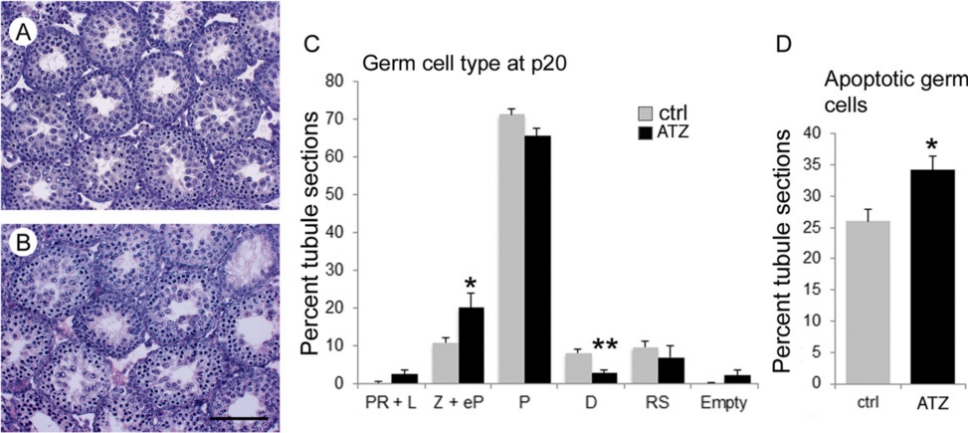
Atrazine delays meiosis in prepubertal mice. H&E straining of histological sections of **a** control and (**b**) ATZ-treated mice. The ATZ-treated mice have reduced numbers of cells in the seminiferous tubules. **c** The percentages of seminiferous tubule cross-sections in which preleptotene and leptotene (PR + L) cells, zygotene and early pachytene (Z + eP) cells, pachytene (P) cells, diplotene **d** spermatocytes and round spermatids (RS) represent the most advanced types of germ cells in control (grey bars) and ATZ-treated (black bars) testes at postnatal day 20. The percentage of empty tubules is also indicated. **d** TUNEL assays on testis histological sections from 20-day-old control and ATZ-treated mice. The results are presented as a percent of tubules containing apoptotic cells, and the data of at least five mice are plotted

To analyze the effect of the ATZ on meiotic progression during the first wave of spermatogenesis, we treated newborn males with ATZ. Young prepubertal animals have a higher proportion of cells at any given meiotic stage because the first wave of spermatogenesis is generally synchronous. The analysis of the testicular sections of treated animals revealed that the number of diplotene cells decreased 2.75 times compared to untreated animals, while the numbers of early pachytene and zygotene cells increased 1.9 times (Fig. 4a-c). These data are consistent with the results obtained from treated adult mice showing that ATZ has a profound effect on meiosis. As the first wave of spermatogenesis involves germ-cell apoptosis to regulate the number of germ cells under normal conditions [74], we assayed this process following ATZ treatment. ATZ treatment of young mice increased the number of apoptotic cells (Fig. 4d) compared to controls. The increased apoptosis rate in young mice is the result of an elevated number of germ cells with meiotic defects, which are eliminated during the first wave of spermatogenesis. Thus, ATZ exposure of young prepubertal mice causes a delay in meiosis similar to that observed in adult males.

### ATZ treatment affects the expression of genes involved in mitochondrial function, steroid-hormone function and GTPase activity

**Fig. 5:**
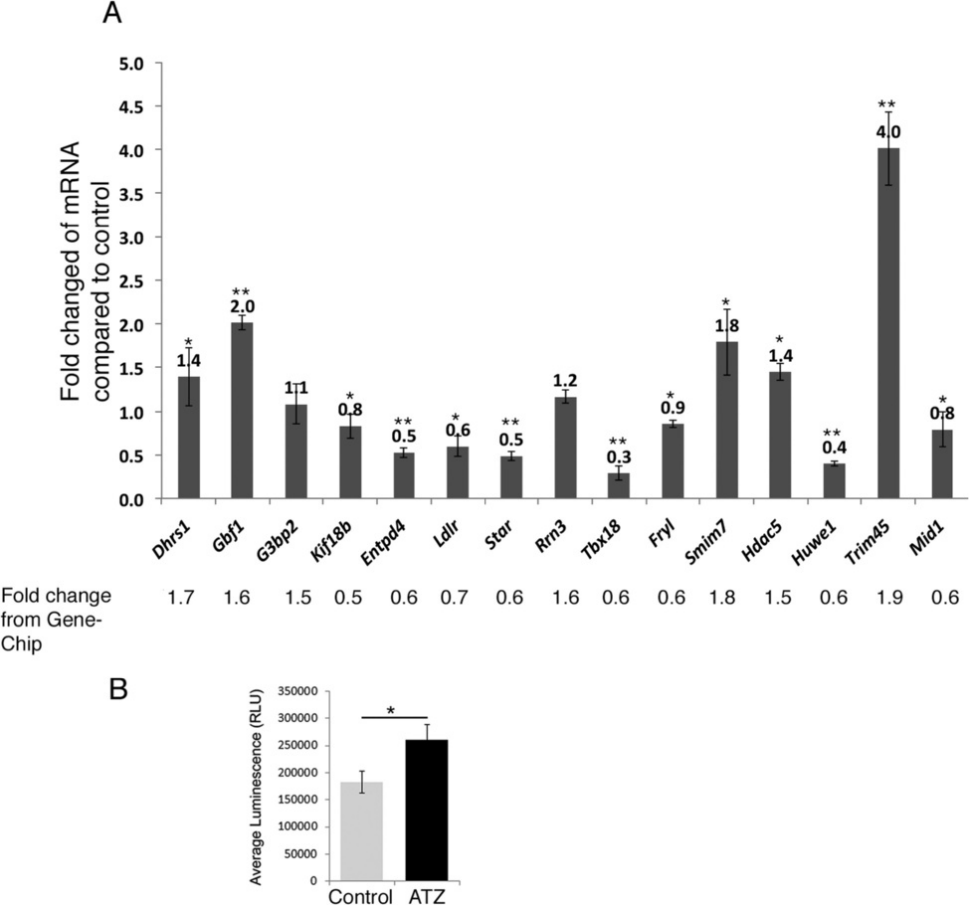
Atrazine affects mRNA transcription and increases production of hydrogen peroxide. **a** qPCR analysis of RNA samples was performed in three independent experiments. qPCR primer sequences for each gene are shown in the Materials and Methods. The data are presented as fold change in treated samples compared to the controls, *p < 0.05, **p < 0.01. **b** Hydrogen peroxide levels increase in the serum of treated animals. The measurement of hydrogen peroxide in the serum of at least three independent experiments is shown. The data are presented as average luminescence values, *p < 0.05

To assess how ATZ affects spermatogenesis at the molecular level, we performed a comparative analysis of gene expression using Gene-Chip analysis. Using a fold-change cutoff value of 1.5 and a *p-*value cutoff of <0.05, we identified 51 genes that were differentially expressed between ATZ-treated and control mice (Additional file 1: Figure S5). The cellular heterogeneity of seminiferous tubules limits the sensitivity of this approach, as patterns of gene expression may vary by cell type. We confirmed differential expression by qPCR (Fig. 5a). Some differentially expressed genes (*Tbx18, Fry1, Rrn3*) were transcription factors, and their roles in spermatogenesis have not yet been elucidated. The expression of oxidative phosphorylation-related genes was increased (*Nd6, Cox18, Dhrs1*). These genes might be activated due to oxidative stress. Oxidative stress is the result of increased production of oxidizing species [11] caused by the metabolism of ATZ [80]. We confirmed that oxidative phosphorylation gene expression was associated with increased ROS by measuring hydrogen peroxide in the serum (Fig. 5b).

The transport of cholesterol across the mitochondrial membrane is a key rate-limiting step in the regulation of steroid-hormone biosynthesis [70, 71, 84]. We found decreased levels of mRNAs encoding *Star,* a major protein responsible for cholesterol transport. The level of *Ldlr* (low-density lipoprotein receptor) also decreased, which affects cholesterol metabolism [35]. Other target genes included small GTPase regulatory proteins, such as *G3bp2*, *Nmt1*, *Sar1a* (Ras GTPase signaling), *Xpo7* (Ran GTPase, RNA export), *Gbf1,* and *Arfgef1* (Arf GTPase protein trafficking). ARF1 GTPase is also required for the maintenance of mitochondrial functionality and dynamics [3].

We performed the analysis by using fold change cut-off value ≥2. The short list includes 9 differentially expressed genes (*Entpd4, Kif18b, 9230104L09Rik, ND6, Acsm2, Speer7-ps1, Speer4e, Xpo7, Gm17019*) (Additional file 1: Table S1). These are genes, which play role in anti-oxidative stress response (*ND6*), xenobiotic metabolism (*Acsm2*), cell division (*Kif18b*), and cell death (*Entpd4*) pathways.

Gene-ChiP analysis revealed that ATZ treatment affects many cellular processes, including steroid-hormone metabolism, oxidative phosphorylation and xenobiotic metabolism.

### Genome-wide sequencing reveals global changes in H3K4me3 marks following ATZ treatment

**Fig. 6:**
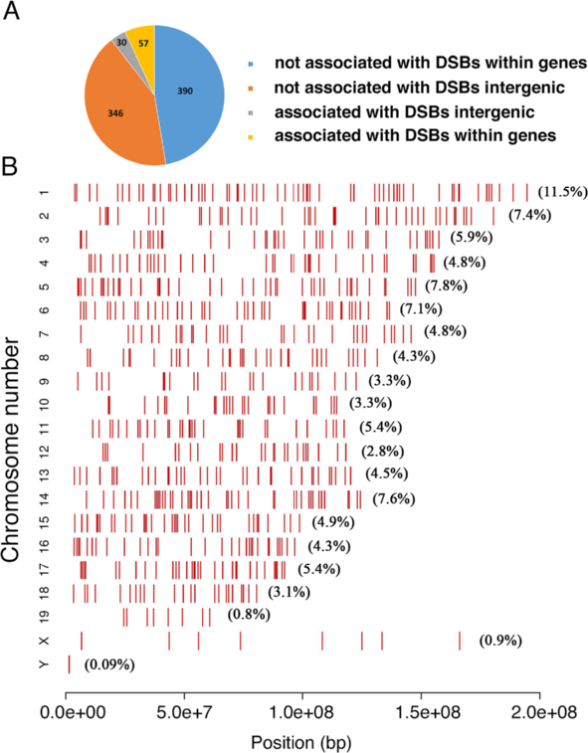
Atrazine globally affects the distributions of H3K4me3 peaks. **a** The distribution of differential H3K4me3 peaks within genic and intergenic regions. The differential peaks were identified as described in the Material and Methods section; the genic regions are sequences located within genes (sequence within gene +/− 1 kb), while the intergenic regions are places that do not overlap the genic regions. The majority of differential peaks are located in the genic regions. **b** The genome-wide distribution of differential H3K4me3 peaks in treated samples: the X-axis shows the chromosome coordinates; the Y-axis shows the chromosome number, and the red bars show the positions of the differential peaks on the chromosomes. The number of peaks per chromosome correlates with chromosome length; the longest, chromosome 1, has the greatest number of differential peaks (numbers in brackets indicate the percent of differential peaks located within a chromosome)

H3K4me3 is a pivotal mark of active chromatin because it acts as a platform for the binding of multiple histone acetyltransferases, histone demethylases and nucleosome-remodeling complexes [44, 100]. As H3K4me3 marks are essential for meiosis, we asked whether treatment with ATZ affects these marks. To this end, we analyzed the genome-wide distribution of H3K4me3 marks in testis tissue. We found that 823 peaks were changed (Fig. 6a and Additional file 1: Files S1 and S2); of these, 799 peaks were increased in ATZ-treated mice, and only 24 were reduced. We confirmed the randomly chosen differential peaks by ChIP-QPCR (Additional file 1: Figure S6) (see Materials and Methods for details).

Among the differential H3K4me3 marks, 447 were located within coding genes (within gene +/−1 kb); this comprised only 54 % of total altered peaks, almost half of which were located in intergenic regions (Fig. 6a). 25.3 % of differential peaks are located within TSSs. Large numbers of peaks were located far from transcription start sites, suggesting that distally located elements, such as enhancers and ncRNA, are affected and may contribute to gene regulation. We compared the differential peaks to known ncRNA maps and found 180 peaks overlapping ncRNA (Additional file 1: File S3). The functions of most ncRNAs have yet to be elucidated.

**Fig. 7:**
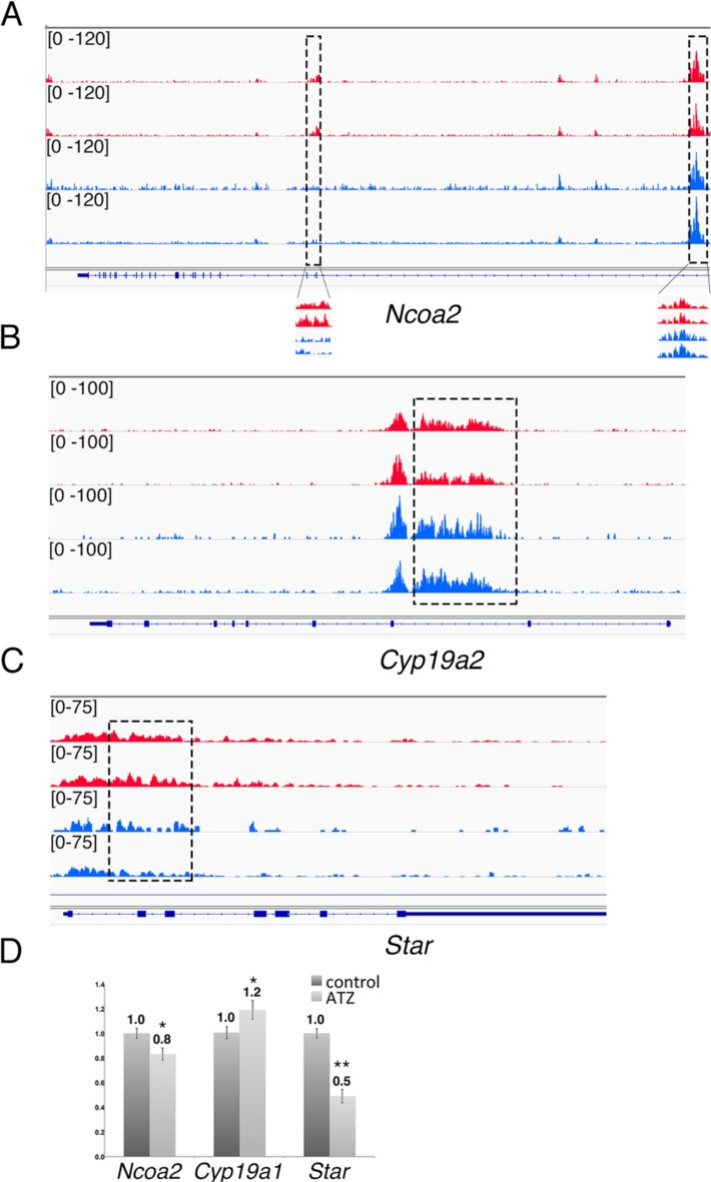
ATZ affects the number of H3K4me3 marks and mRNA levels of genes involved in androgen signaling and testosterone metabolism. **a** The differential H3K4me3 peaks in the *Ncoa2* gene (there are two regions with changes in H3K4me3 marks outlined in boxes), **b** *Cyp19a1* gene and **c** *Star* gene. The Y-axis presents the plotted values of tags at each position after normalizing to all reads in the same dataset (intensity range shown on the left side of the plot); the X-axis shows the schema of the gene; rectangles are exons, and lines are introns. Note that the differential regions are located within genes. The H3K4me3 profiles from control biological replicates are shown in red, and those from ATZ-treated mice are in blue. For illustration, the “Integrative Genomic Viewer” (IGV) software version 2.3.36 was used. **d** qPCR analysis of the *Ncoa2*, *Cyp19a1*, and *Star* genes. qPCR analysis of RNA samples was performed in three independent experiments. Primer sequences for each gene are shown in the Materials and Methods section. The data are presented as fold change in treated samples compared to controls, *p < 0.05, **p < 0.01

Because endocrine function is affected in ATZ-treated animals, we asked whether H3K4me3 peaks changed in the vicinity of steroidogenesis genes or genes regulating steroid-hormone function. Both increased and decreased numbers of H3K4me3 sites were found in different parts of the *Ncoa2* gene (responsible for steroid-dependent gene activation): H3K4me3 marks were reduced at exon 2 and increased near the TSS region in treated animals (Fig. 7a); expression of the latter gene was downregulated. We found increased H3K4me3 marks in *Cyp19a1*, also known as aromatase (Fig. 7b). The number of H3K4m3 marks was also decreased within the *Star* gene (Fig. 7c), a finding in agreement with the microarray data (Additional file 1: Figure S5). Because STAR activity is essential for steroid-hormone biosynthesis, decreased H3K4me3 marks and *Star* transcription suggest that the function of STAR in steroid-hormone metabolism is modified in ATZ-treated animals.

Subsequently, we asked whether differential H3K4me3 regions were located near genes that share common biological functions. We chose the subset of differential peaks within +/−1 kb genes and performed functional annotation using DAVID software (details and parameters of analysis in Material and Methods section). We identified seven significantly enriched Gene Ontology (GO) clusters (Table 1).

**Table 1:** The Gene Ontology (GO) clusters identified by DAVID

Cluster 1	Enrichment Score: 3.18	Count	*P*_Value
GO:0030695	GTPase regulator activity	16	1.10E-04
GO:0060589	nucleoside-triphosphatase regulator activity	16	1.30E-04
GO:0051056	regulation of small GTPase mediated signal transduction	12	1.90E-04
GO:0005085	guanyl-nucleotide exchange factor activity	9	8.40E-04
GO:0046578	regulation of Ras protein signal transduction	9	2.50E-03
GO:0005096	GTPase activator activity	8	1.50E-02
Cluster 2	Enrichment Score: 2.23	Count	*P*_Value
GO:0008104	protein localization	21	1.80E-03
GO:0015031	protein transport	17	1.00E-02
GO:0045184	establishment of protein localization	17	1.10E-02
Cluster 3	Enrichment Score: 2.02	Count	*P*_Value
GO:0005856	cytoskeleton	28	3.70E-03
GO:0043232	intracellular non-membrane-bounded organelle	39	1.50E-02
GO:0043228	non-membrane-bounded organelle	39	1.50E-02
Cluster 4	Enrichment Score: 1.81	Count	*P*_Value
GO:0045185	maintenance of protein location	4	5.50E-03
GO:0051235	maintenance of location	4	8.80E-03
GO:0032507	maintenance of protein location in cell	3	3.00E-02
GO:0051651	maintenance of location in cell	3	4.10E-02
Cluster 5	Enrichment Score: 1.71	Count	*P*_Value
GO:0015629	actin cytoskeleton	9	8.20E-03
GO:0030054	cell junction	14	1.50E-02
GO:0005912	adherens junction	6	1.70E-02
GO:0070161	anchoring junction	6	2.90E-02
GO:0016323	basolateral plasma membrane	6	4.80E-02
Cluster 6	Enrichment Score: 1.62	Count	*P*_Value
GO:0043167	ion binding	67	2.10E-02
GO:0043169	cation binding	66	2.40E-02
GO:0046872	metal ion binding	65	2.80E-02
Cluster 7	Enrichment Score: 1.52	Count	*P*_Value
GO:0006508	proteolysis	24	7.50E-03
GO:0019941	modification-dependent protein catabolic process	13	3.30E-02
GO:0043632	modification-dependent macromolecule catabolic process	13	3.30E-02
GO:0009057	macromolecule catabolic process	15	4.50E-02
GO:0051603	proteolysis involved in cellular protein catabolic process	13	4.50E-02
GO:0044257	cellular protein catabolic process	13	4.70E-02

The most significant GO cluster contained GTPase regulatory activity genes. Most of these genes are related to small Ras GTPase and Arf GTPase signal transduction regulators. Arf1 is required for vesicle transport [64] and mitochondrial homoeostasis [3]. Other enriched clusters include protein transport, maintenance of protein localization, cell junctions, and metal-ion binding (Table 1, clusters 1–6). Enriched cluster 7 contains genes regulating apoptosis and the DNA-damage response. We performed functional annotation of genes located upstream and downstream of ncRNA (Additional file 1: Table S3). We found that differential peaks overlapping ncRNA were surrounded by genes encoding proteins involved in adherens junctions and phagocytosis. Sertoli cells are responsible for the phagocytic elimination of apoptotic spermatogenic cells during normal and pathological conditions [58] suggesting that ATZ may increase the function of phagocytosis.

By using fold change cut-off value ≥2 we found that 45 genomic loci have differential H3K4me3 marks (Additional file 1: Table S2). 7 peaks are located within TSS of some genes (*Speer4d, Dynlt1b, Trim45, Plcb4, Loxl2, Ank3*), 38 peaks are far from TSS of any genes, suggesting the marks at distally located promoters, enhancers and non-coding RNAs are deregulated. The differential peaks are located within genomic regions nearby genes of spermatogenesis function (*Speer* family*, Synb, Ncoa2, Pabpc1, Ropn1, Dhh*); cell adhesion and motility (*Ank3, Pcdhb3, Dynlt1b, Syt17, Stxbp6*); transcription regulation and signaling (*Trim45, Vgll3, Hhat, Plcb4, Nps, Hgf*); oxidoreductase *(Loxl2, Cyp4f18)*, cell cycle and cell death (*Nedd1, Sgol2b, Entpd4*). The analysis confirms that ATZ damage spermatogenesis function most likely due to metabolism of ATZ.

Our data show that ATZ changes the number of H3K4me3 marks in genes with a wide range of important cellular functions.

### Comparison of ChIP-Seq and Gene-Chip data reveal common genes related to GTPase regulation, mitochondrial function and steroid-hormone levels

ChIP-seq and Gene-Chip have different sensitivities. The ChIP-Seq method is very sensitive; it involves integrating a large number of short sequencing reads. Compared to RNA sequencing, the Gene-ChiP assay is less sensitive in detecting genes with very low expression and is less accurate in detecting highly expressed genes [107]. To compare the two datasets, we calculated the averaged signal values of ChIP-Seq peaks at the TSS of differentially expressed genes. We found that 31 genes were present (Table 2) in both datasets, while six differential genes identified by Gene-Chip had no H3K4me3 peaks either because they were encoded by mitochondrial DNA or because no peaks were identified near the TSS. All differentially expressed GTPase-regulating genes had altered H3K4me3 marks, suggesting that the transcription of genes was mediated via epigenetic modifications of histones. However, 13 differential genes had no changes in H3K4me3 marks at their TSS; the expression of mRNA from these genes could be modulated by other mechanisms, such as distally located enhancers, epigenetic mechanisms and ncRNA. Thus, atrazine induces epigenetic modifications at the TSS of the majority of differentially expressed genes.

**Table 2:** The Gene-Chip and ChIP-seq agreement

Affymetrix ID	Official gene name	FC Gene Chip	FC Chip-Seq	Official gene symbol, full description
17550548	*Acsm2*	2.1	1.2	Acyl-coa synthetase medium-chain family member 2
17205319	*Cox18*	1.8	1.1	cytochrome c oxidase assembly protein 18
17200441	*Dhrs1*	1.7	1.1	Dehydrogenase/reductase (SDR family) member 1
17205257	*Gbf1*	1.6	1.2	Golgi-specific brefeldin A-resistance factor 1
17202563	*MrpL27*	1.5	1.2	Mitochondrial ribosomal protein L27
17205437	*Arfgef1*	1.6	1.2	ADP-ribosylation factor guanine nucleotide-exchange factor 1
17201149	*Xpo7*	1.5	1.4	Exportin 7; similar to Ran-binding protein 16
17308165	*Entpd4*	0.4	0.6	Ectonucleoside triphosphate diphosphohydrolase 4
17449641	*G3bp2*	1.5	1.2	Gtpase activating protein (SH3 domain) binding protein 2
17308149	*Gm16677*	0.6	0.6	Predicted gene 16677
17548436	*Gm17019*	2.1	3.7	Predicted gene 17019
17203369	*Sar1a*	1.6	1.1	SAR1 gene homolog A (S. Cerevisiae)
17515315	*Ldlr*	0.7	0.8	Low density lipoprotein receptor
17500195	*Star*	0.6	0.7	Steroidogenic acute regulatory protein
17201763	*Nmt1*	1.6	1.1	N-myristoyltransferase 1
17203277	*Psma1*	1.5	1.1	Proteasome (prosome, macropain) subunit, alpha type 1
17453132	*Psph*	0.6	0.9	Phosphoserine phosphatase
17323063	*Rrn3*	1.6	1.1	RRN3 RNA polymerase I transcription factor homolog (yeast)
17445634	*Gm9758*	1.8	3.1	Spermatogenesis associated glutamate (E)-rich protein 4e
17446524	*Speer4b*	1.6	1.4	Spermatogenesis associated glutamate (E)-rich protein 4b
17445689	*Speer4c*	1.5	1.5	Spermatogenesis associated glutamate (E)-rich protein 4e
17445673	*Speer4e*	2	6	Spermatogenesis associated glutamate (E)-rich protein 4e
17434884	*Speer7-ps1*	2.1	6.2	Spermatogenesis associated glutamate (E)-rich protein 7, pseudogene 1
17434864	*Speer8-ps1*	1.8	2.4	Spermatogenesis associated glutamate (E)-rich protein 8, pseudogene 1
17529481	*Tbx18*	0.6	0.8	T-box18
17201539	*Kif18b*	1.6	1.1	Kinesin family member 18B
17401007	*Trim45*	1.9	2.4	Tripartite motif-containing 45
17535269	*1700020N15Rik*	0.6	1.2	RIKEN cdna 1700020 N15 gene
17201329	*Ccdc103*	0.6	1.2	Coiled-coil domain containing 103
17207313	*Cpsf7*	0.7	1.2	Cleavage and polyadenylation specific factor 7
17201293	*Eftud2*	0.6	1.2	Elongation factor Tu GTP binding domain containing 2
17206471	*Eif5*	0.6	1.2	Similar to Eukaryotic translation initiation factor 5
17448749	*Fryl*	0.6	1.2	Furry homolog-like (Drosophila)
17208347	*Hdac5*	1.5	1	Histone deacetylase 5
17538773	*Huwe1*	0.6	1	HECT, UBA and WWE domain containing 1
17546762	*Mid1*	0.6	1.5	Similar to midline 1
17207105	*Pkd1*	0.7	1	Polycystic kidney disease 1 homolog
17202313	*Psap*	1.7	1	Prosaposin
17204631	*Smim7*	1.8	0.9	Small integral membrane protein 7
17438319	*Tmem165*	0.6	1.1	Transmembrane protein 165
17392600	*9230104L09Rik*	0.5	ND	RIKEN cdna 9230104 L09 gene
17290163	*Ccl28*	1.6	ND	Chemokine (C-C motif) ligand 28
17413061	*Gm5859*	0.5	ND	Similar to 4933409K07Rik protein
17477347	*Klk1b22*	1.8	ND	Kallikrein 1-related peptidase b22
17233226	*Lilrb4*	0.6	ND	Glycoprotein 49 A; leukocyte immunoglobulin-like receptor, subfamily B, member 4
17532649	*Nd6*	2.3	ND	NADH dehydrogenase subunit 6

### Regions showing altered epigenetic marks are enriched at the *Nr5a2*-binding site

**Fig. 8:**
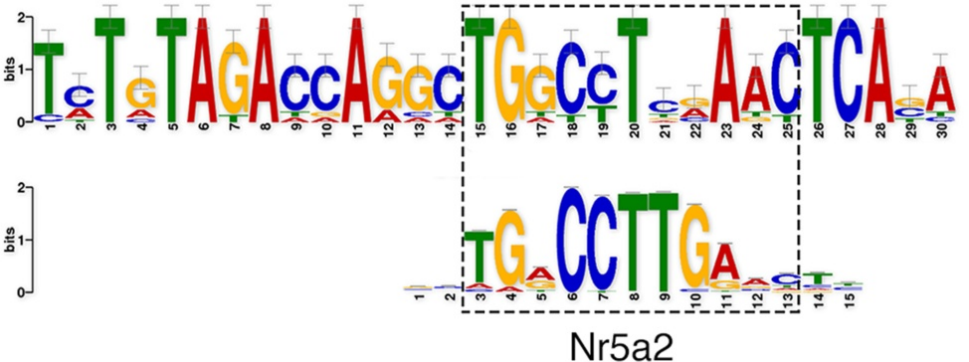
The motif discovered by MEME-ChIP. The top image is a statistically over-represented sequence within the differential peaks discovered by MEME-ChIP [49]. The bottom image is the consensus sequence within the motif discovered by MEME-ChIP. This consensus sequence significantly resembles an *Nr5a2*-binding site

To address whether regulatory DNA motifs are present within regions containing altered epigenetic marks, we used MEME-ChIP [49], which is designed to discover motifs in large sets of DNA sequences. We analyzed the summits of 100-bp sequences within differential peaks. Parameters and details are described in the Materials and Methods. We found a significantly enriched motif among the differential peaks. We compared this motif with known motifs using TomTom [93] and found that part of the discovered motif sequence was significantly similar to the *Nr5a2-*binding site (Fig. 8). Recent work showed that ATZ activates NR5A receptors via phosphorylation and increases cAMP production and PI3K signaling [91]. To further understand the involvement of NR5A2 in the transcriptional regulation of ATZ-treated mice, we performed a functional annotation of genes located near presumed *Nr5a2*-binding sites in ATZ-treated mice. We found enrichment of genes with enzyme inhibitor activity (*Cst13, Cst8, Cst9, Spock3*) and genes encoding proteins involved in the immune response (*Ptprc, Cd55, Daf2*), locomotory behavior (*Nrp2, Ccl21c, Ccl21b, Gm1987, Klhl1*) and lipoprotein metabolism (*Apol10a, Apol9a, Apol7b*) (see also Additional file 1: Table S4).

Our data show that *Nr5a2*-binding sites are significantly enriched in differential peaks in ATZ-treated testes tissue, suggesting an important role for NR5A2 in the transcriptional network of ATZ- treated mice.

### ChIP-Seq reveals deregulation of H3K4me3 marks associated with DSBs

**Fig. 9:**
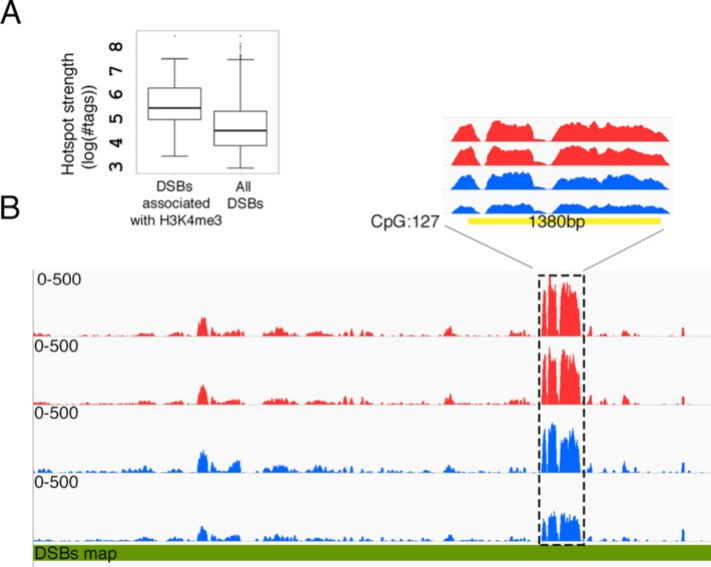
Differential peaks are associated with DSB regions. **a** The association of differential H3K4me3 peaks with the hottest DSB hotspots. The DMC1 tags associated with differential H3K4me3 peaks were extracted from published datasets [12, 86], and intensity values were calculated and presented as log(#tags) on the left plot. The right plot is the intensity of all DSB hotspots signal from the whole dataset [86]. The median signal of DSB peaks associated with differential H3K4me3 peaks was higher than the median signal of all DSBs hotspots, suggesting that differential trimethylation peaks in ATZ-treated mice are associated with the strongest hotspots. **b** The PAR region in ATZ-treated mice has a decreased number of H3K4me3 marks. The DSB map was downloaded from GEO from a previously published work [86]. Plots from controls are in red, plots from treated samples are in blue, and the CpG island is shown in yellow

We compared the altered distribution of H3K4me3 peaks observed in our study to a published DSB-hotspot maps of the C57BL/6 J mouse strain [12, 86]. H3K4me3 marks within 87 regions were elevated in genomic regions overlapping DSB hotspots (Fig. 6a). Fifty-seven of these H3K4me3 peaks were located within genes. Most of the differential H3K4me3 peaks were associated with the strongest DSB hotspots (Fig. 9a).

It is remarkable that H3K4me3 marks at the PAR region of X chromosomes were decreased in ATZ-treated mice (Fig. 9b). This region of PAR contains a CpG island with 127 CpGs.

Interesting, altered H3K4me3 peaks associated with DSBs were located within very large mouse genes (Table 3). *Erbb4* and *Nrxn1* are known to contain common fragile sites (CFS) [88], which are regions of profound genomic instability [45]. Many breakpoints are enriched in AT stretches, which affect replication efficiency by forming secondary structures that stall progression of the replication fork [106]. We examined the AT content in genes and found increased AT content and AT stretches (Table 3).

**Table 3:** The altered H3K4me3 peaks are overlapping with DSB sites in very large genes

Gene	Length (Mb)	Number of DSBs within gene	The highest DSB Signal value within genes	AT content	number of AT stretches
*Erbb4*	1.1	6	15133	64 %	8
*Nrxn1*	1.1	10	2606	63 %	10
*Hs6st3*	0.7	14	2210	59 %	2
*Cntnap5b*	0.7	5	2909	62 %	6
*Hpse2*	0.6	3	3502	60 %	0
*Gmds*	0.5	5	1961	61 %	1
*Dennd1a*	0.5	6	12226	58 %	0

Our data show that ATZ treatment affects H3K4me3 marks at genomic regions overlapping DSBs regions, including PAR and very large genes.

## Discussion

The objective of this study was to investigate the effects of ATZ in meiosis, which is essential for the production of normal gametes. We used atrazine for our study because it is widely distributed; it is the most common pollutant in the environment and is still in use in many countries. Mice were treated with doses lower than those used in many other studies but exceeding the authorized limits. We chose this dose to determine whether it can alter molecular events underlying gametogenesis, although it does not trigger apoptosis or cause obvious changes in testicular morphology. We treated mice for three weeks to cover the period from entry into meiosis until the diplotene stage of prophase I. Specifically, we analyzed meiotic progression, RNA expression and the distribution of genome-wide histone H3K4me3 marks.

### The effects of ATZ on mitochondria and steroid-hormone metabolism

One of the reasons why ATZ is toxic to the testis is its effect on mitochondrial function. Our data suggest that the testicular cells of exposed mice suffer from impaired mitochondrial function, a result consistent with the findings of previous studies [43, 55, 82, 94]. Rats chronically exposed to ATZ have swollen mitochondria with disrupted cristae [47]. Many studies have demonstrated that the oxidative stress induced by atrazine causes increased activity of the antioxidant enzymes superoxide dismutase, catalase, glutathione peroxidase, and glutathione-s-transferase, increasing the production of ROS [2, 39, 85].

We found that exposure of mice to a relatively low dose of ATZ led to diminished testosterone levels. Decreased testosterone can be explained by impaired cholesterol metabolism and increased activity of aromatase CYP19A1, which converts testosterone to estradiol. We found an increase in the level of C*yp19a1* mRNA and increased numbers of H3K4me3 marks in this gene. The increase in CYP19A1 activity after ATZ exposure has been demonstrated in many studies [22, 31, 77, 91]. It was suggested that ATZ activates CYP19A1 via NR5A-receptor binding [23]. There are *Nr5a1*-binding sites in the *Cyp19a1* promoter. However, Pezzi et al. proposed that NR5A2 plays an important role in the regulation of *Cyp19a1* expression in Leydig cells [66]. We also found a putative binding site for NR5A2 within altered H3K4me3 peaks in *Cyp19a1*. Our data support the hypothesis that NR5A2 plays an essential role in activating CYP19A1. It has been suggested that the stimulatory effects of ATZ on the NR5A receptor subfamily (*Nr5a1* and *Nr5a2*) are mediated by receptor phosphorylation, amplification of cAMP and PI3K signaling [91]. These authors proposed that hormone networks are altered in many species exposed to ATZ via the convergence of NR5A activity and cAMP signaling [91]. We found enrichment of several putative NR5A sites near genes encoding proteins involved in the immune response and lipoprotein metabolism. Recent work has shown that the silencing of *Nr5a2* in colon cancer cell lines affects signal transduction, GTPase activity, cholesterol transport, and apolipoprotein expression [9]. We propose that NR5A2 regulates a number of cellular pathways and promotes transcriptional network activation under pathological conditions.

### Effects of ATZ exposure on meiosis and recombination

Specifically, we found increased numbers of cells in the early stages (zygotene and pachytene) of meiosis in ATZ-treated mice. We also detected increased numbers of cells with DMC1 foci on the sex chromosomes. Chromatin at DSB sites contains a special environment near PRDM9-binding motifs, which are nucleosome-depleted [6]. PRDM9-modified nucleosomes create an environment for DNA repair machinery [6]. We suggest that DSB hotspots serve as sensitive targets of DNA-damaging reagents because of their open chromatin structure and enrichment with repeated elements. We found an increase in H3K4me3 at DSB sites associated with strong hotspots, probably reflecting inefficient DSB repair at strong DSB hotspots or repression of HR. If recombination goes wrong way it will be suppressed by cellular mechanisms. Recently, it was found that the helicase *Srs2* in yeast [99], antirecombinase RTEL-1 in *C. elegans* [105], and human *PARI* [54] suppress inappropriate recombination, which is highly toxic to cells as a source of genomic rearrangements.

The disrupting effect of ATZ is most striking at the sex chromosomes. Recombination at sex chromosomes occurs at 40-Mb-long pseudoautosomal regions of X and Y-chromosomes. Recombination at the PAR region differs significantly between sexes: in males, this region has the highest recombination rate [26]. PAR has a different chromatin structure; PAR DNA occupies unusually long chromosomal axes [42], which may play a role in accessibility to DNA damaging factors. DSB formation and repair at PAR is a limiting step in meiosis [42]. ATZ can modulate DSB machinery at PAR. The toxic effect of ATZ on sex chromosomes was previously shown in *Drosophila*, in which it significantly increased X-linked recessive lethality and X or Y loss after treatment by larval feeding [56]. It is also important that the H3K4me3 peak at PAR includes a CpG island, a region where DSB repair could be affected due to chromatin silencing and reduced access to DNA repair machinery [60].

Altered H3K4me3 peaks are associated with DSBs in large genes, such as *Erbb4* and *Nrxn1*. Within human analogues of these genes, there are sites of breakage. Common fragile sites (CFS) are highly conserved between species, and these regions are often enriched in structural variants, including duplications and retrotransposon elements [45]. DNA damage at CFS arises due to exogenous factors, including UV and ionizing radiation, chemotherapy and reactive oxygen species [37]. Exposure to pesticides results in increased susceptibility of fragile sites to breakage in workers exposed to pesticides [57, 79].

It is possible that we failed to detect a set of DSBs not associated with recombination because a set of DSBs induced by ATZ is absent from published datasets. Future work will address this possibility.

In summary, our data elucidates the molecular mechanisms underlying ATZ’s negative effect on meiosis. Importantly, we found a number of effects at the molecular level at a stage in which no morphological changes are detectable.

## Conclusions

In this study, we demonstrated that ATZ affects meiosis in adult and young prepubertal animals. ATZ affects recombination during meiosis and many cellular processes, leading to a decrease in the number of spermatozoa in mice. In addition, the technical approaches used in this research will facilitate the design of studies aimed at understanding the effects of environmental toxicants on reproduction.

## Methods

### Animals and atrazine exposure

All animal procedures were performed according to the guidelines for animal models in research defined by the Ethics Committee and approved by the Ministry of France (reference project number is 01861.02). All experiments were performed by A.G.P. or F.S, who are qualified for laboratory animal care and use. The C57BL/6 J mouse strain was used. Five-week-old adult mice were treated for three weeks with atrazine at a concentration of 100 mg/l in drinking water. The amounts consumed were measured daily, with the ATZ uptake corresponding to nearly 25 mg/kg/days (average uptake 5 ml/day). After three weeks of treatment followed by two weeks without treatment, mice were euthanized, and reproductive organs were dissected. At least five independent experiments were performed with at least six-to-ten animals in each group. Young prepubertal mice were treated from postnatal day 1 until day 20 by administering oil or atrazine suspended in oil to their mothers at a dose of 25 mg/kg/day with at least four animals in each group. The mice were euthanized, and the testes and epididymis were used for analysis.

### Testosterone and FSH quantification

Serum was collected from ketamine/xylazine-anaesthetized adult animals by terminal cardiac exsanguination and aliquots were stored at −20 °C. Testosterone levels in the serum were assayed in duplicate using a commercial radioimmunoassay (RIA) based on competitive binding with I125-labeled testosterone (Immunotech, Beckman Coulter, Villepinte, France), according to the manufacturer’s recommendations. FSH measurements were conducted according a standard protocol provided by a FSH measurement kit (KA2330, Abnova, Walnut CA, USA). In each hormone measurement, the data from at least 10 treated and control animals were averaged and plotted, and the results were expressed in nanograms per milliliter.

### Analysis of histology, apoptosis and numbers of germ cells/Sertoli cells

For the histological analysis, testis samples were fixed in Bouin’s solution and embedded in paraffin. Histological sections (5-μm thick) were stained with hematoxylin and eosin (H&E). The percentage of affected seminiferous tubules was established by counting the H&E-stained cross-sections of seminiferous tubules from the testes of at least three animals. The staging of cells in seminiferous tubes was performed according to an established method [76], with analysis of at least 600 tubule sections. For immunohistochemistry (IHC), the animals were perfused, and the testes were fixed for 24 h in 4 % (wt/vol) paraformaldehyde (PFA) and then embedded in paraffin. For the detection of apoptotic cells, TUNEL assays were performed using the *In Situ* Cell-Death, Fluorescein (Roche Diagnostics, France). For IHC, 5-μm-thick testis sections were incubated overnight at 4 °C with goat anti-PLZF diluted at 1:500 and rat anti-GATA1 diluted at 1:50. The sections were all counterstained with 0.001 % (vol/vol) 4,6-diamidino-2-phenylindole dihydrochloride (DAPI) and mounted in Vectashield (Vector Laboratories, UK).

### Hydrogen peroxide measurement

Measurement of hydrogen peroxide was carried out with the ROS-Glo^TM^H_2_O_2 _Assay kit (Promega, Madison, WI), according to the manufacturer’s instructions. Briefly, 80 μl of serum from adult control and ATZ-treated mice were mixed with 20 μl of H_2_O_2_ substrate and held for 6 h at 37 °C. Then, 100 μl of ROS-Glo^TM^ detection solution was added and incubated for 20 min at room temperature, and relative luminescence was measured in a plate reader.

### Cells sorting by flow cytometry

Germ cells were isolated from control and ATZ-treated testis tissue. The testis cells were subjected to collagenase and DNAse treatment and purified by FACS, as described previously [73]. The dispersed cells were fixed in 1 % (wt/vol) buffered PFA for 20 min at 4 °C and permeabilized in a solution of 0.2 % (wt/vol) saponin containing propidium iodide to label the DNA. The cells were sorted at concentration of 1×10^6^ cells/ml on FACSCalibur cell-sorting machine. The data were analyzed by CellQuestPro software, which was provided with the machine.

### Spermatozoa count

Spermatozoa counts were conducted according to an established protocol [98]. Briefly, the mice were euthanized, and the epididymis was dissected, rapidly frozen in liquid nitrogen and stored at −80 °C until the sperm heads were counted as follows. The organ was first cut with a scalpel into several fragments and homogenized in 50 ml 0.15 M NaCl containing 0.005 % (vol/vol) Triton X-100 (Sigma). After homogenization with three rounds of sonication (12 kHz), an aliquot of the cell suspension was loaded onto a Malassez hemocytometer, and spermatozoa heads were counted. The data from at least ten controls or ATZ-treated animals were averaged and plotted; a *t*-test was then conducted using Excel software.

### Quantitative PCR and microarray experiments

Total RNA was prepared using TRIzol reagent (Life Technologies). Reverse transcription of total RNA was performed using QuantiTect Reverse Transcription (Qiagen) according to the manufacturer’s instructions. Quantitative PCR was performed using the ABI 7500 real-time PCR system. Relative expression values were calculated with ABI SDS software version V2.0.5. The primer sequences used in this research are indicated below. Duplicates of at least three independent experiments were used. Statistical significance was assessed by Student’s *t* test. For Affymetrix microarray experiments, RNA samples were converted to single-stranded biotin-labeled DNA, according to the GeneChip whole-transcript terminal labeling user’s manual, and hybridized to an Affymetrix Gene-Chip mouse gene 2.0 st array. The RNA amplification, biotin labeling and hybridization, washing and scanning were carried out according to Affymetrix protocols. Differentially expressed genes were detected using AMEN software [15] with a fold-change cutoff value of 1.5 and a *p*-value <0.05. The qPCR-quantification Chip-Seq experiments were carried out as previously described [87]. Equal amounts of ChIP and input DNA were taken for PCR. Quantitative PCR was performed using the ABI 7500 real-time PCR system. The copy number for each locus was calculated with ABI SDS software version V2.0.5 using a standard curve. Enrichments were estimated as a ratio of copy number in ChIP samples to the copy number in the input sample. The averaged values of enrichment of at least three treated samples were compared, plotted and expressed as fold change.

The following primers were used for qPCR analysis:

**Table:** 

Gene symbol	5′-- > 3′, forward	5′-- > 3′,reverse
*Dhrs1*	CTGTTCGAGCAAGTAGATCGG	CATAAGTAGTGGCCTCTGAGTC
*Gbf1*	GGTGCAAGTCCCAGGATAAA	GCAGGGTATGCATCAGGTCT
*G3bp2*	TAACAGTGGACAGCCTGAGAG	CATCTTCTGACTCTTCATCAAGTTCTG
*Kif18b*	CCCAGCGACAATACTCCCTG	CCTGAAGACTTAGACTCGGAACAC
*Entpd4*	AAGCTGCCAAGGATTACTGTG	TTTGTAGGTGACAGGAAAGGAG
*Ldlr*	AGGTGTGAAGATATTGACGAGTG	TGAAGAGCAGATAGCCTATGGA
*Star*	TCTCTGCTTGGTTCTCAACTGG	AAACACCTTGCCCACATCTG
*Rrn3*	ACATCATATTGAGATTGCCCTGGT	AGAATCTGAAACATCTATGCCACCT
*Tbx18*	GGGAGGAACAGAATGGGTTTGG	CAGGTGAGGATGTGTAGCAGG
*Fryl*	GATCGCAGTCACCAGGAGCA	AAACACAACGGAGGGATTCTTGG
*Smim7*	AACTTTCCATTCTCCTGCCT	GCCTGAACTTAGACTTGACCC
*Hdac5*	GCATTCTACAACGATCCCTCTG	CACCACTGTCCTGAAGGCTG
*Huwe1*	GCAGAGAACCTCAAGTCAACCA	GCTAAGATCCCGAACAACTCCT
*Trim45*	GCTTTAACGGTAGACCACCTG	CTTCTGTCGCCTATGAGCCTG
*Mid1*	GAAGACCAACAGTCAGCCGT	GAACACGTTGCCAGCCACTC
*Ncoa2*	AAGGCGAAGATTTGCAGTCC	TGTCCAGTGAAGTGATCTTGCC
*Cyp19a1*	CTCATTATCAGCAAGTCCTCAAGCA	TAAAGAAAGGGCGAATTGTTCTCCA
*Star*	TCTCTGCTTGGTTCTCAACTGG	AAACACCTTGCCCACATCTG
Genomic differential peaks coordinates	5′-- > 3′, forward	5′-- > 3′, reverse
chr2a: 57117256-57119105	GCAGATTTACCGCTAGAGAAGGA	CCAAGGGACAGAAGGGAAAGG
chr2b:126752438-126754410	CTTGCTGTGATGTAACAATGCCTG	AGTACCTCTTGCTGAATGAATGTG
chr4:96197763-96198380	AGAGGATAATTACCCTGTACTACCC	GTTCCTTTCTCTGTCTTCAATTCC
chr6a:116626598-116627142	AGGAAGAGGACAAAGGTGGA	CAGGAAGATAAGAAGGTGAGAAGG
chr6b:72197660-72199094	ATTCATGCTTCCCTTCGCCT	TAACACCTACCAGAGCCAACC
chr7:104792205-104794646	TTAATCAGGTGCTCCCTTAAGTCAG	TGAAACAATCACCTGTCTACACCA
chr8:74543719-74544319	CAAGTAAGGGTGGGAGGTGAG	AATGGATGAGGCAACAGTATGAC
chr9:54029137-54032749	CCTGATCTTGAAATGAACCCTACCA	TACACTGTCCACTGTCTCCTCTG
chr13a:120278317-120279418	CCTGCAGAATGCCCAGATTAAA	CCTAAGTAAACCTGAGGTGGACT
chr13b:32360875-32361606	TTGGTTGGTTGATTGGTTGGT	CAGTACGAGTTCTCCTTCTATCCT
chr16:93262599-93264142	CTTAGAACTGGCTTGAGGTGTTGG	TGTGGAGAGGAGTGTGCTTTCAG
chr17a:6429542-6429778	TCTGAGGGAAGAGTAAGACATTTG	CGTGGCTTCAGAGTCTTTACTT
chr17b:91256505-91257806	ACACCTTGGCCTGAATTTGTC	GCACACTTTCAATTTCTCTGACC
chr19:36992713-36994298	CGAATGAAGGCTACGGATGAG	CCTGCTAACTGAGAACACTGG
chrX:166434738-166436029	GCAGTCACTCATGTCATCCC	GTCGCTATGAGTGACTGCTG
chrY:1425238-1426447	CTCTTGATTTGTCTCCTAATTCTCC	CCTCTGTCCCTTTATATGAACC
chr5:143665170-143665301	CACCCATCGCCAAAACTCTTCATCCT	CGCACAGTGCAGCATTTTTTTACC

All Affymetrix microarray data from this study are available and have been deposited in the National Center for Biotechnology Information Gene Expression Omnibus under the following numbers:

**Table:** 

Sample name	Title	Accession number
ATZ_1	Atrazine_treated	GSM1561768
ATZ_2	Atrazine_treated	GSM1561769
ATZ_3	Atrazine_treated	GSM1561770
Ctrl_1	Control	GSM1561771
Ctrl_2	Control	GSM1561772
Ctrl_3	Control	GSM1561773

### Antibodies

The following commercial antibodies were used: goat anti-DMC1 (sc-8973, C-20), goat anti-ATR (sc-1887, N19), mouse anti-SYCP3 (sc-74569, d-1), rat anti-GATA1 (sc-265) antibodies from Santa Cruz. Rabbit anti-H3K4me3 (07–473), from Millipore, rabbit anti-SYCP3 (ab15093) from Abcam, rabbit anti-γH2AX from Trevigen (4411-PC-100) and goat anti-PLZF (AF2944) from R&D systems. Secondary HRP antibodies were purchased from Jackson Laboratories. Fluorescent secondary Alexa antibodies were purchased from Invitrogen.

### Meiotic surface spreads

The seminiferous tubules were chopped in PBS. The cells were then released from the tubules by pipetting and were filtered through a 40-μm cell strainer (Falcon). The cells were pelleted and washed with PBS. The resulting pellet was resuspended in 0.5 % (wt/vol) NaCl, added to glass slides, and allowed to adhere for 10–15 min. The slides were fixed in 2 % (wt/vol) paraformaldehyde with 0.03 % (wt/vol) SDS for 3 min, then 2 % (wt/vol) paraformaldehyde for 3 min and then washed four times in 0.4 % (vol/vol) Photo-Flo 200 (Kodak) for 1 min and air-dried. For quantification of the different γH2AX staining patterns, spermatocyte preparations were analyzed from at least four ATZ-treated mice and controls; at least 600 nuclei were classified according to the criteria described in the text or figure legend. For quantification of the different ATR staining patterns, at least 100 nuclei were classified according to the criteria described in the text or figure legend. We also performed surface spreads as described in a previous study [65] and compared the two methods by analyzing the proportions of cells in different substages. The methods yielded similar results.

### Meiotic prophase I substage analysis

Analysis of the meiotic substages was performed according to previous studies [50, 62].

### Immunofluorescence

The slides were incubated with blocking solution (1 % (vol/vol) donkey serum, 0.3 % (wt/vol) BSA, and 0.005 % (vol/vol) Triton X-100 in PBS) for 20 min at 37 °C in a humidity chamber. The primary antibodies were diluted in blocking buffer and incubated under the same conditions for 1 h. After two 5-min washes in 0.4 % (vol/vol) Photo-Flo/PBS solution, the slides were blocked for an additional 5 min and incubated with secondary antibodies for 20 min at room temperature. The slides were washed twice with 0.4 % (vol/vol) Photo-Flo in PBS, rinsed twice with 0.4 % (vol/vol) Photo-Flo, and allowed to air dry. The images were taken using an AxioImager microscope equipped with an AxioCam MRc5 camera and AxioVision software version 4.8.2 (Zeiss, Le Pecq, France) with a 63X objective lens.

### Chromatin immunoprecipitation and high-throughput sequencing

Chromatin immunoprecipitation was conducted as previously described [12, 87], with small modifications. Testes were fixed for 10 min in 1 % (wt/vol) formaldehyde. After quenching, the tissue was homogenized, filtered through a 40-μm cell strainer, and washed in the following buffers: 1) PBS (twice); 2) 0.25 % (vol/vol) Triton X-100, 10 mM EDTA, 0.5 mM EGTA, 10 mM Tris pH8; 3) 0.2 M NaCl, 1 mM EDTA, 0.5 mM EGTA, 10 mM Tris pH8. Cells were lysed in 1.5 ml of the lysis buffer (1 % (wt/vol) SDS, 10 mM EDTA, and 50 mM TrisCl pH8) with a complete protein-inhibitor cocktail (Roche), and the chromatin was sheared to ~500 bp by sonication. The sample was dialyzed against ChIP buffer (0.01 % (wt/vol) SDS, 1.1 % (vol/vol) Triton X-100, 1.2 mM EDTA, 16.7 mM TrisHCl, 167 mM NaCl). Chromatin was incubated with appropriate antibodies overnight at 4 °C with Dynabead beads (10002D, Invitrogen). The beads were washed in the following buffers: 1) 0.1 % (wt/vol) SDS, 1 % (vol/vol) Triton X-100, 2 mM EDTA, 20 mM TrisHCl, 150 mM NaCl; 2) 0.1 % (wt/vol) SDS, 1 % (vol/vol) Triton X-100, 2 mM EDTA, 20 mM TrisCl pH8, 500 mM NaCl; 3) 0.25 M LiCl, 1 % (vol/vol) Igepal, 1 mM EDTA, 10 mM TrisCl, pH8, 1 % (wt/vol) deoxycholic acid; 4) TE (twice). The chromatin was eluted by 1 % (wt/vol) SDS, 0.1 M NaHCO3 pH9 at 65 °C, and crosslinking was reversed at 65 °C for 5 h. The DNA was deproteinized for 2 h with proteinase K and purified with a MinElute Reaction Clean-Up kit (QIAGEN). The DNA concentration was measured with a Quantus Fluoremeter (E6150, Promega) using the Quantiflor dsDNA system, (E2670, Promega). The libraries were prepared from 50 ng of ChiP and input material using the NEBNext Ultra DNA library Prep Kit for Illumina (NEB #E7370S). The library selection was conducted using excision fragments from a 2 % (wt/vol) agarose gel including GelGreen stain (Gentaur, 41005); 180–220 bp fragments were excised using a Dark Reader transilluminator (Clare Chemical research, Dr-46B). An Illumina Hiseq1500 Genome Analyzer was used to perform massively parallel 50-bp sequencing in Single End mode. We sequenced two biological replicates per condition in multiplexing mode. The reads were demultiplexed and passed through quality control, at which point reads shorter than 50 nucleotides were removed. FastQ files were generated at a genomic platform in Rennes, France.

### Peak calling and differential-peak finding

Between 71 and 97 millions tags were derived from anti-H3K4me3 ChIP and input. The resulting sequences were quality filtered with the Sickle program [40] with –q33 and mapped back to the mouse mm9/NCBI37 genome using Bowtie 1.0.0 with seed length 20. The results were visualized in the Integrative genomics viewer IGV version 2.3.36 [95]. Only tags that passed the quality filter and mapped uniquely to the genome were used. ChIP enrichment was further verified using CHANCE [21]. The H3K4me3 mark peaks were identified using MACS 2.0.1 [24] with two biological replicate samples, including corresponding input, shift-size window 73 bp, no model, with *p*-value threshold <10^−5^. Two biological replicates for each condition were analyzed independently. The set of peaks was verified at an irreproducible discovery rate (IDR) of 0.05 % [46] to confirm that the samples were sufficiently similar to be used in the analysis. The number of mapped reads was multiplied by a scale factor to normalize the total number of reads in different samples. To compare the H3K4me3 CHIP datasets of ATZ-treated and control samples, differential peak calling was performed using several steps. First, from all the peaks called above, we selected the peaks that were reproducible in both datasets in the same condition. From the dataset of selected peaks, we kept only peaks with average expression values above the 5 % quantile; last, we selected the peaks with fold changes above 1.5 and FDR <5 %. Statistical significance was calculated using a Limma test [89]. Annotation of significant differential peaks was performed by CEAS [83]. All sequencing data from this study are publicly available and have been deposited in the National Center for Biotechnology Information Gene Expression Omnibus.

The following samples from ChIP seq data were deposited at GEO:

**Table:** 

Sample name	Title	Accession number
ATZ_1	Atrazine_treated	GSM1563221
ATZ_2	Atrazine_treated	GSM1563222
Input	Atrazine_treated	GSM1563225
Ctrl_1	Control	GSM1563223
Ctrl_2	Control	GSM1563224
Input	Control	GSM1563226

### Gene Ontology (GO) term analyses

Gene Ontology (GO) term analyses were performed using DAVID v6.7 [33] with enrichment thresholds ease 0.05, kappa index 3. Functional classification was performed with the same subset of the gene list with parameter kappa similarity 5 and initial group number 5.

### Meme-ChiP motif search

For the analysis, we used the summits of 100-bp sequences from differential peaks, excluding DSB-associated peaks. Motif finding was performed with MEME-ChIP [49] following default parameters. Identified motifs were compared with known motifs using TomTom [93] with a *q* - value  less than 0.003.

All sequencing and Gene-Chips data from this study are publicly available and have been deposited in the National Center for Biotechnology Information Gene Expression Omnibus, GO number: GSE 64037.

## Additional file

Additional file 1: **Supplementary material.** (PDF 2459 kb)

## Abbreviations

DSB: Double-strand DNA break
PAR: Pseudoautosomal region
ATZ: Atrazine
