# Supplementary material for: The epigenetic processes of meiosis in male mice are broadly affected by the widely used herbicide atrazine
Source: BMC Genomics. 2015 Oct 30;16:885. doi: 10.1186/s12864-015-2095-y (PMC4628360; doi:10.1186/s12864-015-2095-y)
Supplement: Supplementary file 1 — Supplementary material. (PDF 2459 kb) [file 12864_2015_2095_MOESM1_ESM.pdf]

Table S1

Differentially expressed genes in ATZ treated testes, cut-off value ≥2

Gene Name	FC	Gene full name/ function
Entpd4	0.4	Ectonucleoside triphosphate diphosphohydrolase 4, role in salvaging of nucleotides, localizes to lysosomal/autophagic vacuoles
Kif18b	0.5	Kinesin family member 18B, role in spindle formation during mitosis
9230104L09Rik	0.5	RIKEN cDNA 9230104L09 gene, cysteine proteinase inhibitor
ND6	2.3	NADH dehydrogenase subunit 6, oxidative phosphorylation Complex I
Acsm2	2.1	Acyl-CoA synthetase medium-chain family member 2, xenobiotic/medium-chain fatty acid:CoA ligase
Speer7-ps1	2.1	Spermatogenesis associated glutamate (E)-rich protein 7, pseudogene 1
Speer4e	2.0	Spermatogenesis associated glutamate (E)-rich protein 4e
Xpo7	2.0	Exportin 7, Ran GTPase binding, nuclear transport receptor
Gm17019	2.1	Predicted gene 17019

Table S2

Differential H3K4me3 peaks, cut-off value ≥2

chr	start	end	FC	Nearest genes	Function
chr1	13214621	13215994	0.2	*Ncoa2*	Gene encodes nuclear receptor coactivator 2, which aids in the function of nuclear hormone receptors, gene is essential for mouse reproductive function
chr14	69909210	69910543	0.3	*Synb*	The protein plays a major role in placental development and trophoblast fusion and essential for reproduction
chr14	69943852	69944785	0.4	*Entpd4*	This gene encodes a member of the apyrase protein family, which may play a role in salvaging nucleotides from lysosomes and contribute cell death
chr15	36579531	36580059	0.5	*Pabpc1*	Pabpc1 protein may plays role in translational regulation of gene expression during spermatogenesis
chr16	65839556	65839983	0.5	*Vgll3*	Vgll3 expression is associated with a tumor suppressor phenotype in epithelial ovarian cancer, plays role in transcription regulation
chr16	34657682	34658027	0.5	*Ropn1*	Ropporin, a sperm-specific binding protein of rhophilin, that is localized in the fibrous sheath of sperm flagella
chr14	70008610	70009670	0.5	*Loxl2**	This gene encodes a member of the lysyl oxidase gene family, cell growth control. LOXL2 promotes tumor progression possibly by activating multiple signal pathways, oxidoreductase
chr10	69070299	69070962	0.5	*Ank3**	Ankyrins play key roles in activities such as cell motility, activation, proliferation, contact, and the maintenance of specialized membrane domains
chr19	25724428	25725066	0.5	*2610016A17Rik*	RIKEN cDNA 2610016A17 gene
chr5	15217817	15218108	6.2	*Speer7-ps1*	Spermatogenesis associated glutamate (E)-rich protein 4C, opposite strand transcript
chr5	14936867	14937118	6.0	*Speer4e*	Spermatogenesis associated glutamate (E)-rich protein 4e
chr5	14977994	14978281	5.7	*Speer8-ps1*	Spermatogenesis associated glutamate (E)-rich protein 8, pseudogene 2
chr5	14978623	14978993	4.8	*Speer8-ps1*	Spermatogenesis associated glutamate (E)-rich protein 8, pseudogene 1
chr13	120278317	120279418	4.1	*Gm7120*	predicted gene 7120
chr5	15124816	15125295	3.5	*Speer4d**	Spermatogenesis associated glutamate (E)-rich protein
chr17	6494046	6494217	3.4	*Tmem181b-ps*	Transmembrane protein 181B, pseudogene
chr5	15125523	15125706	3.2	*Speer4d*	Spermatogenesis associated glutamate (E)-rich protein 4D
chr5	14914917	14915152	3.1	*Gm9758*	Predicted gene 9758
chr5	15032099	15032493	2.9	*Gm17019*	Predicted gene 17020
chr17	6491637	6491975	2.5	*Tmem181b-ps*	Transmembrane protein 181B, pseudogene
chr18	37463167	37463871	2.8	*Pcdhb3*	Pcdhb3 may play a critical role in the establishment and function of specific cell-cell connections, cell adhesion
chr5	14936238	14936480	2.6	*Speer4e*	Spermatogenesis associated glutamate (E)-rich protein 4e
chr17	6429542	6429778	2.6	*Dynlt1b**	Dynlt1b plays a key role in the dynein-independent function of Tctex-1
chr1	194608391	194608626	2.5	*Hhat*	Skinny hedgehog encodes an enzyme that acts within the secretory pathway to catalyze amino-terminal palmitoylation of 'hedgehog'
chr5	14937599	14937829	2.5	*Speer4e*	Spermatogenesis associated glutamate (E)-rich protein 4e
chr5	14976373	14976907	2.5	*Speer8-ps1*	Spermatogenesis associated glutamate (E)-rich protein 8, pseudogene 1
chr3	100723591	100731489	2.5	*Trim45**	The encoded protein may function as a transcriptional repressor of the mitogen-activated protein kinase pathway
chr5	14944534	14944797	2.4	*Speer8-ps1*	Spermatogenesis associated glutamate (E)-rich protein 8, pseudogene 1
chr17	6492231	6492684	2.4	*Tmem181b-ps*	Transmembrane protein 181B, pseudogene
chr8	73608544	73608886	2.3	*1700026F02Rik*	RIKEN cDNA 1700026F02 gene
chr15	98721379	98721524	2.3	*Dhh*	Desert Hedgehog/Patched 1 signaling specifies fetal Leydig cell fate in testis organogenesis, combinatorial expression of the paracrine factor Dhh and nuclear transcription factor Sf1 is required for Leydig cell development
chr2	135536752	135537192	2.3	*Plcb4**	Plcb4 has a role in phosphatidylinositol phospholipase C activity
chr8	66190792	66191168	2.2	*Sgol2b*	Shugoshin like 2b (S. pombe) may play role in cell cycle
chr8	74543719	74544319	2.2	*Cyp4f18*	Cyp4f18 is necessary for omega oxidation of leukotriene B4 in neutrophils, oxidoreductase activity
chr5	15031093	15031323	2.2	*Gm17019*	Predicted gene 17019
chr7	142450407	142451206	2.2	*Nps*	Nps plays role GPCR signalling
chr7	125554914	125555453	2.2	*Syt17*	Syt17 (Synaptotagmin XVII) play role in transporter activity
chr17	6493079	6493317	2.2	*Tmem181b-ps*	Transmembrane protein 181B, pseudogene
chr17	6475846	6476233	2.2	*Tmem181b-ps*	Transmembrane protein 181B, pseudogene
chr5	16072354	16072830	2.1	*Hgf*	Hepatocyte growth factor regulates cell growth, cell motility, and morphogenesis by activating a tyrosine kinase signaling cascade after binding to the proto-oncogenic c-Met receptor
chr10	92038881	92039318	2.1	*Nedd1*	Nedd1 play role in cell cycle
chr4	37196302	37197507	2.0	*Lingo2*	Lingo2 play role in early development
chr8	68941877	68942087	2.0	*March1*	March1 is a member of membrane-bound E3 ubiquitin ligases
chr12	46228758	46229166	2.0	*Stxbp6*	Stxbp6 plays role in vesicle-mediated transport, phosphatidylinositol-4,5-bisphosphate binding
chr14	44121518	44121728	2.0	*Gm10375*	Predicted gene 10375

* Peaks are located within TSS

Table S3

Functional classification of genes located nearby ncRNAs

GO term number	GO term name	Genes are in cluster	P-Value
GO:0005912	adherens junction	*Ptprc, Tln2, Dlg5, Flnb*	0.004
GO:0006909	phagocytosis	*Cdc42se2, Elmo1, Elmod1*	0.010
GO:0046907	intracellular protein transport	*Slc25a13, Erbb2ip, Stam, Arfip1, Tnpo1, Hook3*	0.056
GO:0005743	mitochondrial inner membrane	*Nat8b, Fech, Slc25a13, Au042651*	0.064
GO:0006468	protein amino acid phosphorylation	*Irak4, Ptprc, Ick, Csnk1e, 4921509C19Rik, Pim1*	0.05

Table S4

Functional annotation of genes with putative Nr5a2 binding site

Go Term number	GO term name	Genes are in cluster	P-Value
GO:0004857	enzyme inhibitor activity	Cst13, Cst8, Cst9, Spock3	0.04
GO:0002253	activation of immune response	Ptprc, Cd55, Daf2	0.03
GO:0007626	locomotory behavior	Nrp2, Ccl21c, Ccl21b, Gm1987, Klhl1	0.05
GO:0042157	lipoprotein metabolic process	Apol10A, Apol9A, Apol7B	0.03

Fig. S1

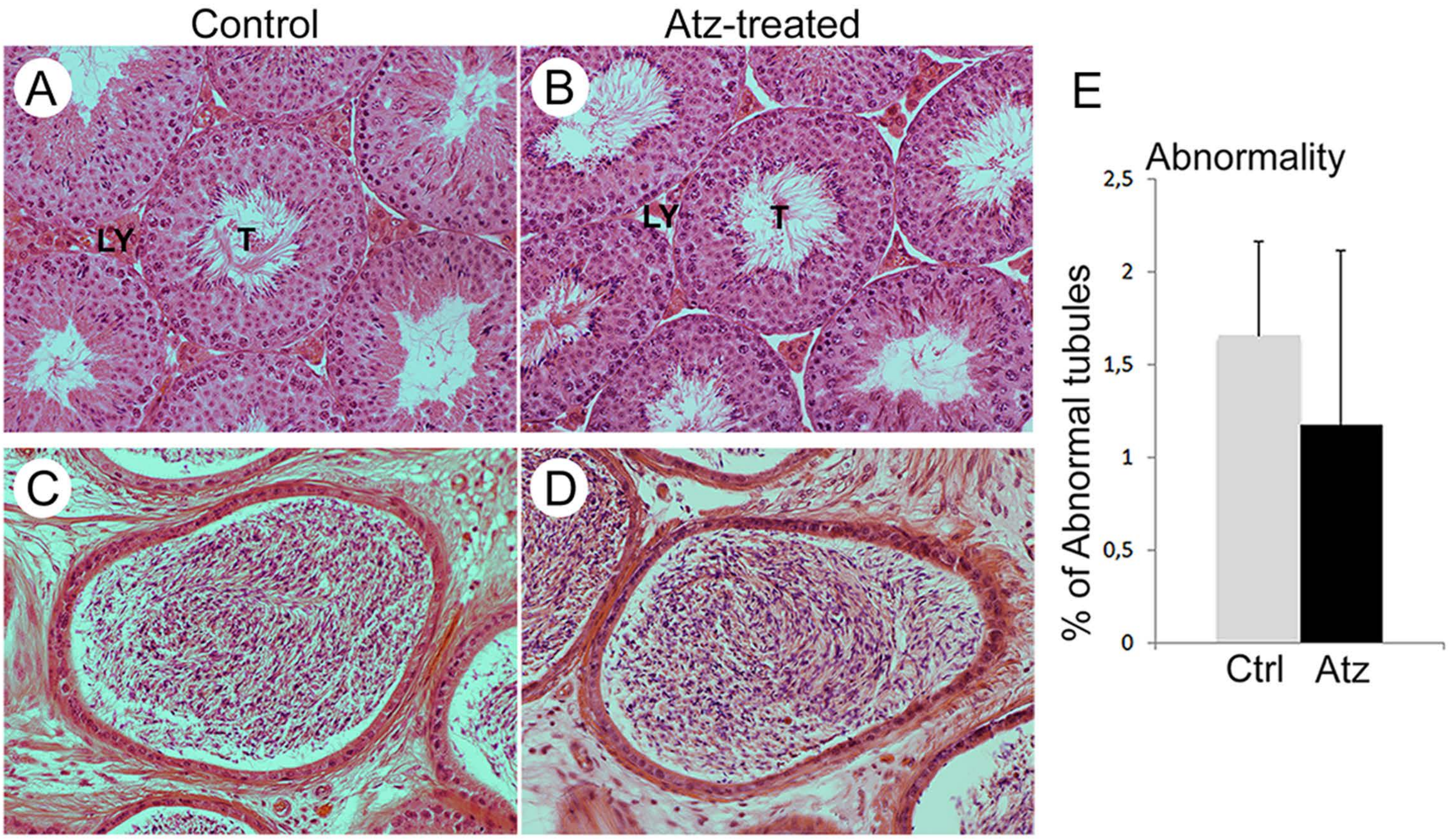

Fig. S1. Low-dose ATZ treatment doesn't affect the morphology of reproductive organs. A-D, Histological H&E stained sections of testis (A-B) and epididymis (C,D), (E) The percentage of abnormal tubules sections in ATZ-treated mice compared to the control. The abnormal tubules correspond to the sections where one or more germ cell layers were missing.

Fig. S2

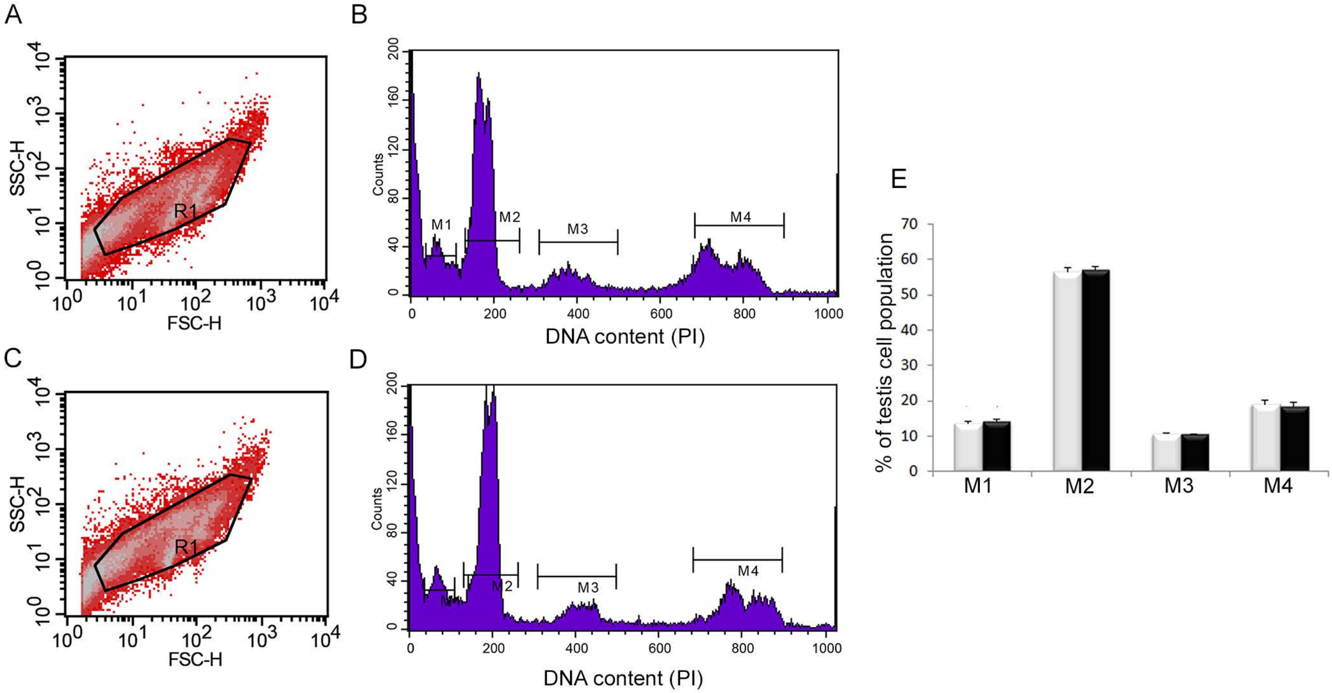

Fig. S2. Low-dose of atrazine doesn't affect cells population in semineferous tubules. (A-D) The example of flow cytometric analysis of germ cells from (A-B) control and (C-D) treated mice. The same populations of cells were analyzed for each sample. The DNA content of cells was evaluated by propidium iodide incorporation (PI), four populations of cells were detected: M1, spermatozoa, M2, haploid cells, M3, diploid cells and M4, tetraploid cells (meiotic cells). (E) A quantitative analysis of each cell types; n=10.

Fig. S3

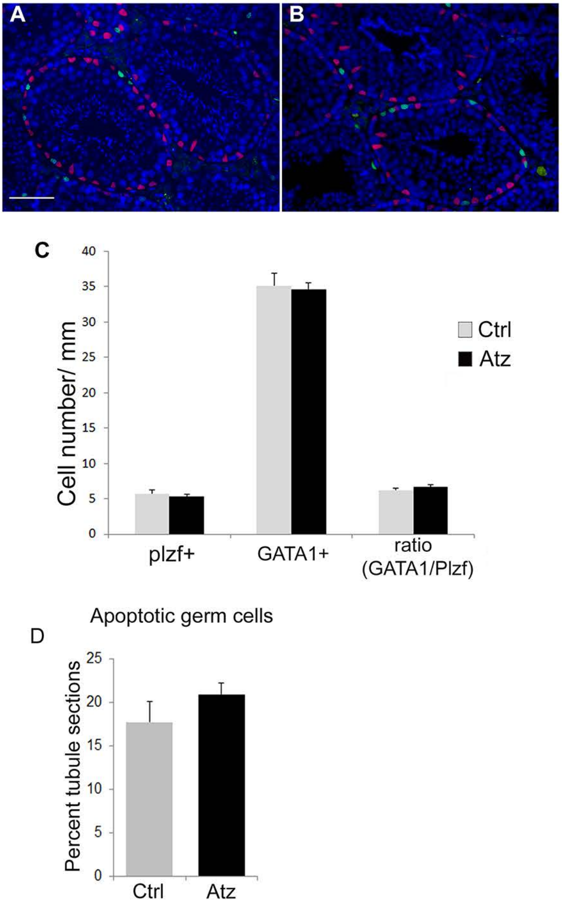

Fig. S3. Atrazine at low-dose doesn't affect the numbers of the undifferentiated spermatogonia and Sertoli cells. (A-B) Histological sections of (A) control and (B) ATZ-treated testes. Undifferentiated spermatogonia were labeled with anti-PLZF antibody (in green), Sertoli cells were labeled with anti- GATA1 (in red) antibodies (C) Labeled Sertoli and spermatogonia cells were counted at stage VII, the contours of each tubule section were measured using the Image J software. The data show the number of cell counts per millimeter of tubule circumference, scale bar: 50μm. (D) The TUNEL assays of testes sections. The results of at least 4 experiments were counted and presented as the percent of tubules containing apoptotic cells.

Fig.S4

A

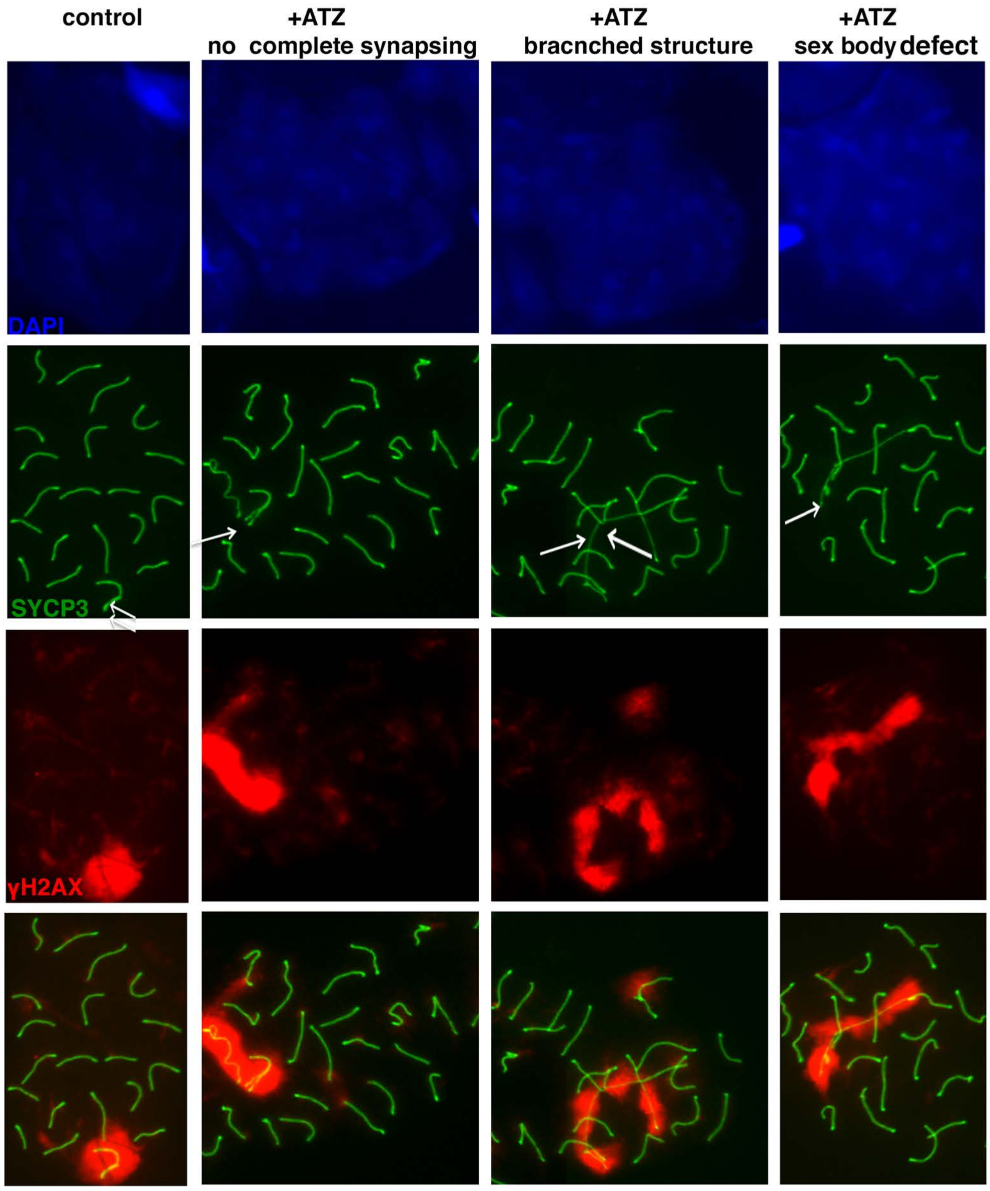

B

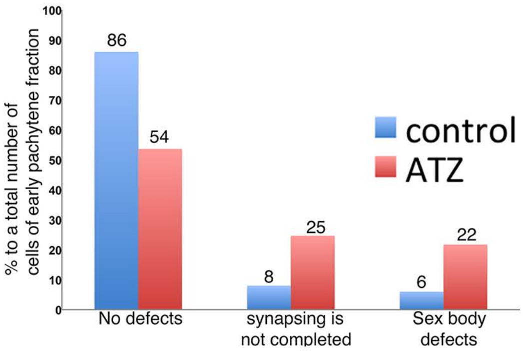

Fig. S4. Atrazine affects the chromosomes synapsing. (A) The analysis of γH2AX and SYCP3 pattern of early pachytene staining: In control samples γH2AX staining is weak at autosomal chromosomes but strong at sex body (first panel), in treated mice there is increase of cells with defects (1) single unsynapsed chromosomes, which show strong γH2AX staining at autosomes (second panel), (2) there is the increase of cells with branched structures (third panel), (3) sex body has a long axes. (B) The distribution of defects was estimated in 100 cells at early pachytene stage from four independent experiments.

ID	GeneName	ATZ_1	ATZ_2	ATZ_3	Ctrl_1	Ctrl_2	Ctrl_3	FC Gene Chip
17207105	*Pkd1*	5.8	6.7	6.2	6.9	6.7	7.0	0.68
17206471	*Eif5*	6.3	5.7	6.0	6.4	7.0	6.9	0.60
17233226	*Lilrb4*	6.2	6.1	5.8	6.9	6.7	6.5	0.63
17413061	*Gm5859*	6.0	6.0	5.9	6.7	7.1	6.6	0.55
17201329	*Ccdc103*	6.5	7.0	7.1	7.6	7.6	7.7	0.61
17529481	*Tbx18*	7.1	6.9	6.9	7.7	7.5	7.5	0.65
17207313	*Cpsf7*	6.4	6.3	6.6	7.1	7.2	6.8	0.65
17535269	*1700020N15Rik*	6.7	6.4	6.6	7.5	7.1	7.0	0.63
17201293	*Eftud2*	6.1	7.0	6.1	7.0	7.0	7.6	0.59
17308165	*Entpd4*	6.1	6.4	6.2	7.6	7.6	7.5	0.40
17308149	*Gm16677*	10.7	10.7	10.5	11.5	11.3	11.4	0.60
17500195	*Star*	10.5	10.5	10.3	11.3	11.3	10.6	0.63
17538773	*Huwe1*	9.6	8.7	9.1	9.6	10.0	9.8	0.64
17392600	*9230104L09Rik*	8.4	8.3	8.6	9.8	9.0	9.0	0.54
17546762	*Mid1*	8.5	8.3	8.0	9.0	9.2	8.7	0.60
17438319	*Tmem165*	8.0	7.7	7.7	8.6	8.7	8.4	0.58
17448749	*Fryl*	8.2	8.0	7.6	8.9	9.0	8.5	0.56
17515315	*Ldlr*	7.7	7.6	7.5	8.3	8.5	7.8	0.66
17453132	*Psph*	7.6	7.4	7.8	8.1	8.9	7.9	0.58
17477347	*Klk1b22*	10.7	10.4	10.2	9.6	9.5	9.7	1.81
17445673	*Speer4e*	10.1	10.2	10.4	9.3	8.9	9.3	2.02
17449641	*G3bp2*	9.7	9.8	10.0	9.4	8.6	9.5	1.55
17550548	*Acsm2*	8.3	9.7	8.6	8.0	7.6	8.1	2.13
17434884	*Speer7-ps1*	8.6	8.6	8.8	7.6	7.5	7.7	2.11
17401007	*Trim45*	8.5	8.7	8.9	7.7	7.8	8.1	1.86
17445689	*Speer4c*	8.4	8.7	8.5	7.9	7.8	8.0	1.54
17532649	*ND6*	8.8	10.5	9.3	8.5	8.2	8.8	2.32
17290163	*Ccl28*	9.1	9.3	9.1	8.7	8.4	8.4	1.55
17203277	*Psma1*	6.6	7.3	7.0	6.2	6.5	6.3	1.54
17203369	*Sar1a*	6.7	7.2	7.4	6.6	6.6	6.3	1.56
17200441	*Dhrs1*	6.9	7.1	7.3	5.8	6.7	6.3	1.75
17201149	*Xpo7*	6.8	6.9	7.0	6.1	6.6	6.1	1.52
17205437	*Arfgef1*	6.9	6.7	6.9	6.1	6.3	6.2	1.57
17205257	*Gbf1*	6.7	6.7	7.2	6.5	5.6	6.3	1.64
17201239	*Xpo7*	7.0	7.0	6.2	6.4	5.0	5.5	2.00
17201539	*Kif18b*	6.7	7.1	6.3	6.2	6.0	6.0	1.56
17208347	*Hdac5*	6.8	6.9	6.6	6.6	5.5	6.2	1.53
17201763	*Nmt1*	8.0	7.8	8.4	7.4	7.1	7.6	1.61
17548436	*Gm17019*	8.1	8.5	8.7	7.2	7.1	7.7	2.09
17202313	*Psap*	7.7	8.3	8.0	7.1	7.0	7.5	1.75
17445634	*Gm9758*	8.0	8.6	8.0	7.5	7.1	7.7	1.77
17434864	*Speer8-ps1*	7.1	7.9	7.3	6.4	6.9	6.5	1.79
17446524	*Speer4b*	7.1	7.7	7.0	6.7	6.5	6.8	1.60
17323063	*Rrn3*	6.9	7.9	7.4	7.0	6.7	6.6	1.61
17202563	*MrpL27*	7.5	7.3	7.8	7.1	6.7	7.0	1.54
17204631	*Smim7*	7.3	7.7	8.2	6.7	7.0	7.1	1.79
17205319	*Cox18*	7.3	8.1	8.3	7.1	7.2	7.0	1.81

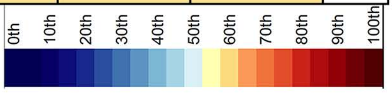

Fig. S5. Atrazine affects the genes expression. The differentially expressed genes were identified by Gene-Chip method. The first column shows the Affymetrix ID number, the second column shows the official gene symbols. The log2 scale of GeneChip values is given for ATZ or control samples. The fold changes of gene expression are shown in a last column.

Fig.S6

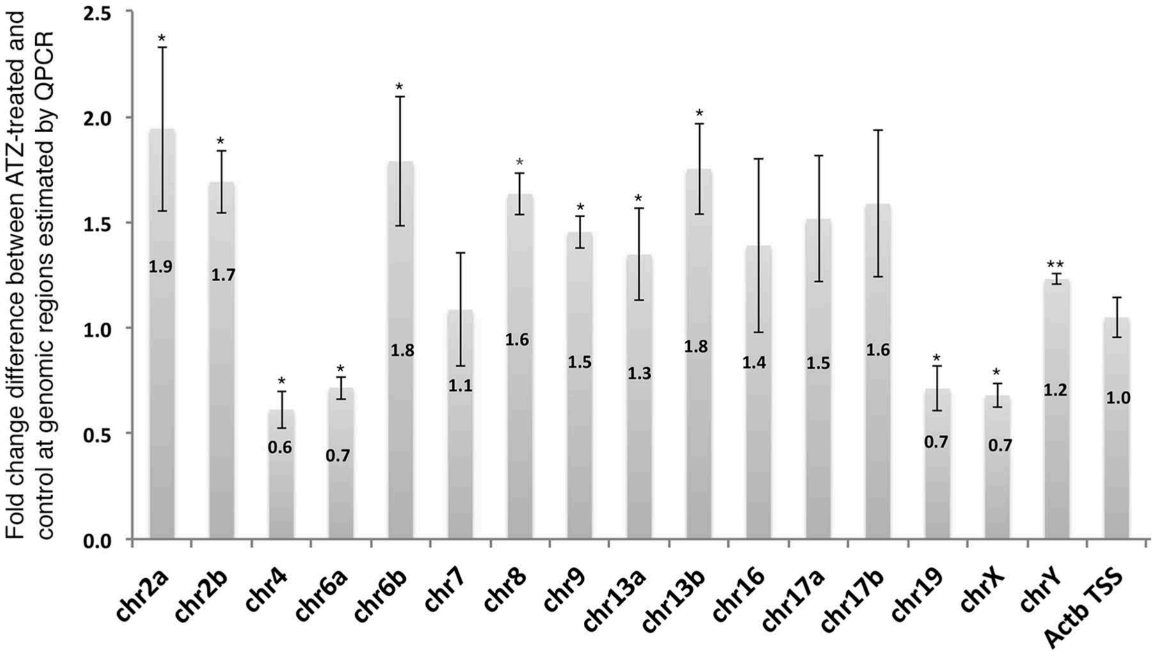

Fold change Estimated by ChIP-seq 1.6 1.5 0.6 1.9 2.2 1.6 2.2 1.6 1.9 2.6 1.6 1.6 1.6 0.5 0.6 1.5

Fig. S6. ChIP-qPCR confirmation of identified differential H3K4me3 peaks. ChIPs were performed from 3 biological replicates of control and treated testes (details in Materials and Methods section). The equal amounts of DNA of ChIP and Input material were taken for qPCR. The ChIP efficiency was estimated by performing qPCR analysis of enrichment of promoters DNA of ActB, housekeeping gene, and as negative control the region of genome with absence any H3K4me3 signal was used. The enrichemnt of *Actb* is similar in both sets of samples and there is no enrichment of negative region (data not shown). ChIP-qPCR was performed as described in Materials and Methods section. Enrichments were estimated as a copy numbers in ChIP samples compared to the copy numbers in the input samples. The averaged values of enrichment of at least three treated samples were compared, plotted and expressed as fold change.

		Suppplementary file 1. Differential H3K4me3 peaks are not associated with DSBs							
Chromosome	Start	End	Fold_Change	H3K4me3_Accu-mulated	H3K4me3_Within_genes	H3K4me3_nearest_upstream_gene	Distance_to_nearest_upstream_gene	H3K4me3_nearest_downstream_gene	Distance to nearest_downstream_gene
chr5	15217817	15218108	6.2	ATZ	Speer7-ps1	4930572O03Rik	54940	4930519H02Rik	151638
chr5	14936867	14937118	6.0	ATZ	Speer4e	Gm9758	21978	Speer8-ps1	8176
chr5	14977994	14978281	5.7	ATZ	Speer8-ps1	Speer4e	39519	Gm17019	50669
chr5	14978623	14978993	4.8	ATZ	NA	Speer8-ps1	82	Gm17019	49957
chr1	13214621	13215994	4.2	cont	Ncoa2	Prdm14	97377	Tram1	338789
chr5	15124816	15125295	3.5	ATZ	Speer4d	Gm17019	91809	4930572O03Rik	32811
chr17	6494046	6494217	3.4	ATZ	NA	Tmem181b-ps	43904	Dynlt1f	106579
chr5	15125523	15125706	3.2	ATZ	Speer4d	Gm17019	92516	4930572O03Rik	32400
chr5	14914917	14915152	3.1	ATZ	NA	Gm9758	28	Speer4e	18479
chr14	69909210	69910543	3.0	cont	Synb	Slc25a37	6050	Gm16677	37221
chr5	15032099	15032493	2.9	ATZ	Gm17019	Speer8-ps1	53558	Speer4d	92424
chr18	37463167	37463871	2.8	ATZ	Pcdha4-g Pcdhb3	Pcdhb2	5899	Pcdhb4	3238
chr14	69943852	69944785	2.7	cont	NA	Synb	31496	Gm16677	2979
chr5	14936238	14936480	2.6	ATZ	Speer4e	Gm9758	21349	Speer8-ps1	8814
chr17	6429542	6429778	2.6	ATZ	Dynlt1b	Dynlt1a	112068	Tmem181b-ps	8372
chr5	14937599	14937829	2.5	ATZ	Speer4e	Gm9758	22710	Speer8-ps1	7465
chr3	100723591	100731489	2.5	ATZ	Trim45	Vtcn1	22746	Ttf2	11294
chr1	194608391	194608626	2.5	ATZ	NA	Hhat	10978	1700065J18Rik	59273
chr17	6491637	6491975	2.5	ATZ	NA	Tmem181b-ps	41495	Dynlt1f	108821
chr5	14976373	14976907	2.5	ATZ	Speer8-ps1	Speer4e	37898	Gm17019	52043
chr5	14944534	14944797	2.4	ATZ	NA	Speer4e	6059	Speer8-ps1	497
chr17	6492231	6492684	2.4	ATZ	NA	Tmem181b-ps	42089	Dynlt1f	108112
chr15	98721379	98721524	2.3	ATZ	NA	Rhebl1	9534	Dhh	1934
chr8	73608544	73608886	2.3	ATZ	NA	1700026F02Rik	57890	Haus8	166137
chr2	135536752	135537192	2.3	ATZ	NA	9630028H03Rik	127796	Plcb4	30374
chr17	6493079	6493317	2.2	ATZ	NA	Tmem181b-ps	42937	Dynlt1f	107479
chr8	66190792	66191168	2.2	ATZ	NA	Spock3	354899	Gm4975	212323
chr5	15031093	15031323	2.2	ATZ	Gm17019	Speer8-ps1	52552	Speer4d	93594
chr7	142450407	142451206	2.2	ATZ	NA	1700120G07Rik	62722	Nps	9096
chr15	36579531	36580059	2.2	cont	NA	Pabpc1	40803	Ywhaz	119957
chr16	65839556	65839983	2.2	cont	Vgll3	Chmp2b	276614	Cadm2	815683
chr17	6475846	6476233	2.2	ATZ	NA	Tmem181b-ps	25704	Dynlt1f	124563
chr7	125554914	125555453	2.2	ATZ	Syt17	4930583K01Rik	165849	Itpripl2	73173
chr5	16072354	16072830	2.1	ATZ	Hgf	Cacna2d1	192025	Speer4f	909110
chr16	34657682	34658027	2.1	cont	Ropn1	Kalrn	143569	Ccdc14	32675
chr14	70008610	70009670	2.1	cont	Loxl2	Entpd4	23811	R3hcc1	87441
chr10	69070299	69070962	2.1	cont	Ank3	Cdk1	254639	Ccdc6	488907
chr8	68941877	68942087	2.0	ATZ	March1	BC030870	1288441	Tma16	58158
chr4	37196302	37197507	2.0	ATZ	NA	Lingo2	297525	1700009N14Rik	2199819
chr14	44121518	44121728	2.0	ATZ	NA	1700001F09Rik	95174	Gm10375	58564
chr19	25724428	25725066	2.0	cont	NA	Dmrt3	26017	2610016A17Rik	20558
chr10	85270633	85270855	2.0	ATZ	NA	Btbd11	147596	Pwp1	63721
chr11	11385440	11385926	2.0	ATZ	NA	Zpbp	23018	4930415F15Rik	3343
chr19	36992713	36994298	2.0	cont	Fgfbp3	Tnks2	24746	Btaf1	6271
chr16	3542341	3542982	2.0	ATZ	NA	NA	NA	Olfr161	49416
chr6	72197660	72199094	1.9	ATZ	NA	Atoh8	12089	Sftpb	54148

chr5	86337839	86338066	1.9	ATZ	NA	Epha5	1491432	Cenpc1	102984
chr5	89172263	89172670	1.9	ATZ	Mob1b	Grsf1	67067	Dck	21368
chr16	12757420	12757654	1.9	ATZ	NA	Shisa9	486423	1700003L19Rik	53829
chr17	6489260	6489496	1.9	ATZ	NA	Tmem181b-ps	39118	Dynlt1f	111300
chr1	50163539	50163765	1.9	ATZ	NA	Slc39a10	3253185	Tmeff2	820602
chr17	80513489	80513760	1.9	ATZ	NA	Hnrpll	51815	Galm	13051
chr7	138757178	138757585	1.9	ATZ	NA	Bub3	41766	Gpr26	400558
chr3	145364044	145365224	1.9	ATZ	NA	Cyr61	51095	Ddah1	56432
chr13	106679224	106679563	1.9	ATZ	NA	Htr1a	441011	Ipo11	904956
chr13	37806834	37809620	1.9	ATZ	NA	Ly86	295929	Rreb1	108287
chr15	61847741	61849545	1.9	ATZ	NA	Myc	25825	Pvt1	19997
chr4	14721166	14721604	1.9	ATZ	Lrrc69	Slc26a7	172241	Otud6b	15048
chrX	125046743	125046996	1.9	ATZ	NA	4921511C20Rik	1116313	4930558G05Rik	539064
chr6	112602449	112602734	1.9	ATZ	Rad18	Oxtr	162647	Srgap3	65232
chr9	122560789	122561878	1.9	ATZ	NA	9530059O14Rik	71024	Mir138-1	30116
chr2	103328416	103330526	1.9	ATZ	NA	Cat	3106	Abtb2	75941
chr16	93262599	93264142	1.9	ATZ	NA	Runx1	436288	1810053B23Rik	79819
chr14	103513557	103515202	1.9	ATZ	Mycbp2	Fbxl3	14831	Scel	397356
chr15	25337053	25337643	1.9	ATZ	Basp1	Cdh18	1932880	Gm5468	6304
chr1	134121907	134122204	1.9	ATZ	Lemd1	Mir135b	27146	Klhdc8a	72999
chr5	11256119	11256449	1.9	ATZ	NA	Gm6455	385311	Speer1-ps1	83959
chr2	60618485	60619430	1.9	ATZ	Mir684-1 Rbms1	Itgb6	57825	Gm13582	183003
chr17	38059443	38060367	1.9	ATZ	NA	Olfr127	17979	Olfr128	146
chr10	105801438	105802642	1.8	ATZ	NA	Ccdc59	516872	Ppfia2	104724
chr14	102641288	102642068	1.8	ATZ	NA	Lmo7	307378	Kctd12	733730
chr8	119526236	119526479	1.8	ATZ	Pkd1l2	Gcsh	8887	Bcmo1	93286
chr16	11106589	11107457	1.8	ATZ	Txndc11	Snn	31511	Zc3h7a	29230
chr11	30471470	30471863	1.8	ATZ	Acyp2	4930505A04Rik	99641	Psme4	199912
chr4	102815586	102817300	1.8	ATZ	Mier1	Wdr78	28682	Slc35d1	27023
chr7	28522343	28523104	1.8	ATZ	Zfp60	C030039L03Rik	30842	Gm10046	27588
chr1	132317624	132318013	1.8	ATZ	Daf2	Thsd7b	201769	Cd55	17594
chr2	156217533	156217700	1.8	ATZ	2900097C17Rik	Cnbd2	16159	Epb4.1l1	29088
chr2	171059519	171060824	1.8	ATZ	NA	Dok5	354244	1700028P15Rik	721555
chr17	6476411	6476645	1.8	ATZ	NA	Tmem181b-ps	26269	Dynlt1f	124151
chr9	77655868	77656207	1.8	ATZ	NA	Gclc	13572	Elovl5	108965
chr14	96282579	96283239	1.8	ATZ	NA	4921530L21Rik	585	Klhl1	221245
chr15	57833085	57834200	1.8	ATZ	Fam83a	Wdr67	31462	9130401M01Rik	19627
chr14	57070152	57070756	1.8	ATZ	Rnf17	Atp12a	62764	Cenpj	74842
chr2	21991213	21991801	1.8	ATZ	NA	Gpr158	239044	Myo3a	157329
chr14	69953593	69956310	1.8	cont	Entpd4 Gm16677	Synb	41237	Loxl2	52973
chr1	138500886	138501281	1.8	ATZ	NA	Kif14	72798	Zfp281	20197
chr17	88602063	88602668	1.8	ATZ	NA	Gm4832	71607	Foxn2	237384
chr5	14945193	14945946	1.8	ATZ	Speer8-ps1	Speer4e	6718	Gm17019	83004
chr16	38407586	38408059	1.8	ATZ	Pla1a	Popdc2	29284	Adprh	37420
chr12	102287546	102288352	1.8	ATZ	Smek1	Ccdc88c	20353	D130020L05Rik	32309
chr7	126889145	126890054	1.8	ATZ	NA	Thumpd1	24866	Acsm3	14383
chr1	13352269	13352932	1.8	ATZ	Ncoa2	Prdm14	235025	Tram1	201851
chr14	72768642	72769553	1.8	ATZ	NA	4930434J06Rik	448397	Fndc3a	168207
chr3	35840290	35840911	1.8	ATZ	NA	Dcun1d1	8402	Mccc1	17320
chr5	104941675	104942629	1.8	ATZ	BC005561	Pkd2	6837	D930016D06Rik	12125

chr17	83847965	83848917	1.8	ATZ	Eml4	Pkdcc	223556	Cox7a2l	52340
chr14	104397145	104397593	1.8	ATZ	4930432J09Rik	Ednrb	153452	Pou4f1	463948
chr3	148175723	148176498	1.8	ATZ	NA	Ttll7	1530009	Lphn2	302052
chr3	89564549	89566063	1.8	ATZ	Chrnb2	Adar	7172	Ube2q1	11468
chr5	47783319	47783794	1.8	ATZ	NA	Lcorl	1534540	Slit2	590600
chr18	27139320	27140251	1.8	ATZ	NA	Celf4	1226836	4930474G06Rik	1578414
chr13	19530318	19530677	1.8	ATZ	NA	Stard3nl	42697	Epdr1	152900
chr2	169065091	169066880	1.8	ATZ	NA	1700017J07Rik	260685	Tshz2	392266
chr11	49195710	49197180	1.8	ATZ	NA	Olfr1390	40739	Olfr1389	46749
chr18	80450663	80451116	1.8	ATZ	NA	Hsbp1l1	6822	Pqlc1	868
chr7	65025550	65026024	1.8	ATZ	Gabrb3	Gabra5	260171	Atp10a	887548
chr3	35597128	35598415	1.8	ATZ	NA	Gm6639	32043	Atp11b	54645
chr4	42087908	42088257	1.8	cont	4933409K07Rik	Gm13308	274498	Gm1987	142786
chr13	74684021	74684602	1.8	ATZ	NA	Zfp825	52623	Erap1	92718
chr17	6429271	6429489	1.8	ATZ	Dynlt1b	Dynlt1a	111797	Tmem181b-ps	8661
chr8	51601691	51602594	1.8	ATZ	NA	Gm2516	100248	Aga	2994462
chr6	34731741	34732540	1.8	ATZ	Agbl3	Cald1	6272	Tmem140	80606
chr14	69900581	69904584	1.8	cont	Slc25a37	Nkx3-1	87866	Synb	3871
chr7	68117865	68118774	1.8	ATZ	NA	Snrpn	832760	B230209E15Rik	555522
chr9	113617811	113618749	1.8	ATZ	NA	Pdcd6ip	434	Clasp2	31842
chr18	77094514	77095422	1.8	cont	Skor2	Smad2	623113	Ier3ip1	73344
chr11	73506782	73507813	1.8	ATZ	Zfp735	Olfr384	89760	Olfr389	82076
chr6	57428835	57429249	1.8	ATZ	NA	Vmn1r20	46239	Ppm1k	27247
chr6	57426415	57427090	1.8	ATZ	NA	Vmn1r20	43819	Ppm1k	29406
chr7	46667691	46667977	1.8	ATZ	Gm5114	Gm21276	147787	Zfp939	36911
chr14	97450641	97451506	1.8	ATZ	NA	Klhl1	532388	Dach1	734560
chr2	101000357	101002460	1.8	ATZ	NA	Lrrc4c	3528536	B230118H07Rik	398478
chr6	52857336	52860198	1.8	ATZ	Jazf1	Tax1bp1	140563	Gm4872	310960
chr2	65106788	65107361	1.8	ATZ	Mir684-1	Cobll1	30105	Slc38a11	47329
chr17	38040471	38040854	1.8	ATZ	Olfr127	Olfr126	51973	Olfr128	19659
chr8	126329483	126330677	1.7	ATZ	Rab4a	6030466F02Rik	65642	Ccsap	34067
chr1	138469744	138470787	1.7	ATZ	NA	Kif14	41656	Zfp281	50691
chr15	14388872	14390787	1.7	ATZ	NA	Cdh6	1285478	Cdh9	2317069
chr14	104437127	104438748	1.7	ATZ	NA	4930432J09Rik	10768	Pou4f1	422793
chr6	135609075	135610578	1.7	ATZ	NA	Emp1	275884	Grin2b	69245
chr11	101983975	101985159	1.7	ATZ	NA	Pyy	14885	Nags	21668
chr5	144412066	144413482	1.7	ATZ	Cyth3	Fam220a	86673	Usp42	57713
chr12	114782407	114783417	1.7	ATZ	Adam6a	Adam6b	52361	Zfp386	2502518
chr9	5120690	5122480	1.7	ATZ	NA	Gria4	324456	Casp1	176037
chr3	84183136	84183927	1.7	ATZ	4930565D16Rik	Trim2	74439	Fhdc1	62191
chr12	70166635	70167966	1.7	ATZ	NA	Gm17821	1396377	Rps29	90743
chr6	49439913	49440484	1.7	ATZ	NA	Stk31	20412	Npy	332244
chr11	84013887	84014648	1.7	ATZ	Acaca	Gm11437	32909	Aatf	221710
chr19	57644795	57645770	1.7	ATZ	NA	6720468P15Rik	57517	Atrnl1	39754
chr2	168023061	168024440	1.7	ATZ	Adnp	E130018N17Rik	43048	Dpm1	10108
chr8	131552476	131552963	1.7	ATZ	Ccdc7	Itgb1	294997	1700008F21Rik	38071
chr13	86706309	86707209	1.7	ATZ	NA	Cox7c	519909	Edil3	2253868
chr6	100072847	100075871	1.7	ATZ	NA	Prok2	396461	Rybp	102688
chr11	43090622	43091789	1.7	ATZ	NA	Atp10b	14835	Mir146	96110
chr6	47072868	47073123	1.7	ATZ	Cntnap2	Tpk1	3456694	Cul1	331200
chr7	31948073	31948979	1.7	ATZ	Gm10640	Gramd1a	12004	Scgb1b2	126559
chr5	15510247	15511699	1.7	ATZ	Cacna2d1	4930519H02Rik	120663	Hgf	547669

chr15	80650055	80652695	1.7	ATZ	Tnrc6b	Fam83f	119200	Mir5113	117774
chr1	37614857	37617687	1.7	ATZ	NA	Mgat4a	21753	2010300C02Rik	50834
chr7	48288116	48289359	1.7	ATZ	Gm4884	4930433I11Rik	37913	Gm5592	250338
chr13	48621523	48622032	1.7	ATZ	NA	Zfp169	12718	Mirlet7d	9349
chr13	3607049	3608474	1.7	ATZ	BC016423	Gdi2	41542	Asb13	24804
chr2	113125638	113127207	1.7	ATZ	Tmco5b	Ryr3	255150	Fmn1	40686
chr2	113927143	113929560	1.7	ATZ	Aqr	C130080G10Rik	39905	Zfp770	89637
chr13	103930028	103930674	1.7	cont	Mast4	Cd180	433317	Srek1	601021
chr18	67708306	67709050	1.7	ATZ	Spire1	Slmo1	68071	Cep76	68001
chr4	58560065	58561619	1.7	ATZ	Lpar1	Mir3095	106095	Olfr267	236032
chr5	39697186	39697578	1.7	ATZ	NA	Clnk	429254	4930513D17Rik	155410
chr6	79098702	79099811	1.7	ATZ	NA	Gm20362	112839	Gm20594	668214
chr10	104751393	104753044	1.7	ATZ	Tmtc2	Gm20765 Gm2	1117817	Gm15663	258563
chr15	9083841	9086297	1.7	ATZ	Lmbrd2	Skp2	13634	Ugt3a1	123287
chr15	3808941	3809403	1.7	ATZ	NA	Ghr	275711	Fbxo4	104161
chrX	55981083	55981440	1.7	ATZ	NA	Zic3	691276	4930550L24Rik	183198
chr12	87588060	87588790	1.7	ATZ	Gpatch2l	Gm805	52008	Esrrb	113277
chr4	87196895	87197341	1.7	ATZ	NA	Slc24a2	320451	Mllt3	218488
chr6	18817948	18818577	1.7	ATZ	Ankrd7	Naa38	13896	Kcnd2	2347532
chr18	22969705	22970634	1.7	ATZ	Nol4	Asxl3	280977	Dtna	603282
chr6	40848538	40850874	1.7	ATZ	Prss58	Moxd2	11045	1700074P13Rik	19585
chr15	50189298	50190448	1.7	ATZ	NA	Csmd3	1565763	Trps1	295857
chr9	55661663	55662115	1.7	ATZ	Scaper	Isl2	267678	Rfpl3s	148281
chr16	38894108	38894591	1.7	ATZ	NA	4930435E12Rik	65246	Igsf11	7867
chr6	40877973	40878730	1.7	ATZ	1700074P13Rik	Prss58	27587	1810009J06Rik	36041
chr12	82478891	82479238	1.7	ATZ	Gm4787	Slc8a3	44724	Adam4	41298
chr15	41563247	41564593	1.7	ATZ	Oxr1	Zfpm2	627109	Abra	132246
chr1	179921652	179921924	1.7	ATZ	Gm16432	Adss	195012	Desi2	195624
chr1	46723911	46724242	1.7	ATZ	NA	Dnahc7b	293516	Slc39a10	140147
chr4	102413398	102415183	1.7	ATZ	NA	Pde4b	133531	Sgip1	17785
chr18	45861158	45861734	1.7	ATZ	A330093E20Rik	Kcnn2	15621	Trim36	463220
chr7	134491512	134492379	1.7	ATZ	NA	Zfp768	2684	Zfp747	23672
chr9	55858768	55859124	1.7	ATZ	NA	Rfpl3s	30029	Gm7444	16319
chr8	123225295	123226578	1.7	ATZ	NA	Cox4i1	27186	Irf8	33698
chr16	24721439	24723477	1.7	ATZ	Lpp	A730098P11Rik	186881	A230028O05Rik	336248
chr1	182991730	182992858	1.7	ATZ	NA	9130409I23Rik	932	Nvl	30696
chr8	26700821	26701414	1.7	ATZ	Letm2	Fgfr1	14635	Whsc1l1	11364
chr14	44377752	44378086	1.7	ATZ	NA	Gm10375	192074	Ear1	18345
chr15	45808319	45809425	1.7	ATZ	NA	Kcnv1	861839	1700022A22Rik	346706
chr1	146597447	146598780	1.7	ATZ	NA	Rgs1	501213	Rgs18	1191
chr9	118241610	118242468	1.7	ATZ	NA	Cmc1	182296	4933432G23Rik	96489
chr11	25740027	25740661	1.7	ATZ	5730522E02Rik	4933427E13Rik	472890	Fancl	546423
chr3	157512913	157515312	1.7	ATZ	NA	Ptger3	205191	Cth	41900
chr17	7151832	7152054	1.7	ATZ	Rsph3b	Mir692-1	52146	Tagap1	7260
chr2	74145133	74145843	1.7	ATZ	4930441J16Rik	Atp5g3	395782	Lnp	207051
chr2	41350136	41351239	1.7	ATZ	Lrp1b Mir684-1	Ppp6c	2268278	Kynu	2059610
chr7	46570637	46571072	1.7	ATZ	NA	Gm21276	50733	Gm5114	91592
chr10	76920427	76921708	1.7	ATZ	NA	Gm17769	36743	Fam207a	27692
chr8	94093291	94095124	1.7	ATZ	Fto	Rpgrip1l	256170	Irx3	227286
chr8	84730718	84732474	1.7	ATZ	NA	Inpp4b	84258	Il15	123049
chr5	39239690	39240402	1.7	ATZ	Clnk	Zfp518b	163625	4930513D17Rik	612586
chr5	5076030	5076398	1.7	ATZ	Cdk14	Fzd1	317814	1700015F17Rik	361429

chr2	146113613	146115587	1.7	ATZ	Ralgapa2	A930019D19Rik	20717	4933406D12Rik	253080
chr14	15942542	15943519	1.7	ATZ	NA	Nek10	103335	Lrrc3b	246512
chr10	70409180	70409842	1.7	cont	Bicc1	D630013N20Rik	290469	C730027H18Rik	221643
chr7	53682403	53683553	1.7	ATZ	Kcnc1	Myod1	47941	Sergef	14976
chr6	6243671	6244169	1.7	ATZ	NA	Slc25a13	76498	Shfm1	264106
chr11	11364223	11364954	1.7	ATZ	NA	Zpbp	1801	4930415F15Rik	24315
chr4	84509387	84510265	1.7	ATZ	NA	Bnc2	188397	Cntln	19966
chr14	45120024	45120479	1.7	ATZ	BC061237	1700047E10Rik	329122	Gm8267	216363
chr7	74187318	74189756	1.7	ATZ	Adamts17	Cers3	218740	Lysmd4	177674
chr15	51829878	51832906	1.7	ATZ	NA	Rad21	6572	Aard	38747
chr12	65048892	65050025	1.7	ATZ	NA	Lrfn5	2102647	Fscb	522295
chr5	20149397	20150712	1.7	ATZ	Magi2	Gnai1	2283166	Phtf2	113770
chr2	68494659	68497174	1.7	ATZ	4932414N04Rik	4933409G03Rik	40139	Cers6	202440
chr2	65107486	65108183	1.7	ATZ	Mir684-1	Cobll1	30803	Slc38a11	46507
chr5	84241499	84242309	1.7	ATZ	NA	Tecrl	457279	Epha5	241479
chr9	67542172	67543343	1.7	ATZ	NA	Tln2	134662	C2cd4b	63901
chr17	22655565	22656648	1.7	ATZ	NA	Zfp946	61909	Vmn2r111	28260
chr13	112277401	112278213	1.7	ATZ	Gpbp1	Actbl2	229444	Mier3	198173
chr3	28792548	28794186	1.7	ATZ	Gm1527	Rpl22l1	86211	Egfem1	187313
chr16	9035020	9036278	1.7	ATZ	NA	1810013L24Rik	176003	Grin2a	541525
chr5	79011809	79012603	1.7	ATZ	NA	Pea15b	1071036	Lphn3	2438015
chr8	68943451	68944005	1.7	ATZ	March1	BC030870	1290015	Tma16	56240
chr14	99488842	99489368	1.7	ATZ	Dis3	Bora	15516	Pibf1	9284
chr6	40878942	40880372	1.7	ATZ	1700074P13Rik	Prss58	28556	1810009J06Rik	34399
chr17	46469000	46469865	1.7	ATZ	NA	Abcc10	4028	Zfp318	50850
chr17	38088958	38089459	1.7	ATZ	Olfr761	Olfr128	27519	Olfr129	102085
chr3	135213575	135213834	1.7	ATZ	Manba	Ube2d3	83433	Nfkb1	33785
chr11	72423270	72423799	1.7	ATZ	Ube2g1	Spns3	59522	Ankfy1	79705
chr4	34501000	34501901	1.7	ATZ	Akirin2	Spaca1	363958	Orc3	12129
chr17	62796310	62798491	1.7	ATZ	NA	Nudt12	3643565	Efna5	153815
chr13	95317580	95319459	1.7	ATZ	Ap3b1	Gm9776	188702	Tbca	239439
chr10	70412049	70413235	1.6	ATZ	Bicc1	D630013N20Rik	293338	C730027H18Rik	218250
chr7	48409328	48409749	1.6	ATZ	NA	Gm4884	108656	Gm5592	129948
chr11	74744736	74745811	1.6	ATZ	Smg6	Srr	5436	Hic1	232256
chr6	127749306	127749457	1.6	cont	NA	Prmt8	30129	Tspan11	88183
chr12	17613628	17613934	1.6	ATZ	NA	Odc1	55320	Hpcal1	83686
chr13	8856679	8858877	1.6	ATZ	Wdr37	Adarb2	97125	Idi1	25975
chr2	16838314	16839115	1.6	ATZ	NA	Plxdc2	164572	4930515L03Rik	1190
chr16	58719238	58719934	1.6	ATZ	NA	Gpr15	401	Cldn25	8089
chr13	49999668	50000245	1.6	ATZ	NA	Iars	170032	Gm906	340305
chr13	76408015	76408452	1.6	ATZ	NA	Ttc37	82584	Mctp1	113957
chr5	8178113	8178615	1.6	ATZ	Adam22	Sri	108868	Dbf4	218354
chr16	4658820	4659610	1.6	ATZ	Coro7	Vasn	7654	Dnaja3	24460
chr6	116537492	116539733	1.6	ATZ	NA	Olfr215	4530	Zfp422	34301
chr8	60007688	60008195	1.6	ATZ	Galnt7	Hmgb2	12892	AW046200	122355
chr3	107350845	107351688	1.6	ATZ	NA	Slc6a17	29909	Ubl4b	4928
chr2	80475903	80476675	1.6	ATZ	NA	Dusp19	4085	Nup35	2294
chr5	134825120	134825489	1.6	ATZ	NA	Gtf2i	34504	Gtf2ird1	8042
chr7	142448929	142450184	1.6	ATZ	NA	1700120G07Rik	61244	Nps	10118
chr14	24937827	24938301	1.6	ATZ	NA	Kcnma1	114400	Dlg5	14874
chr9	13105243	13106913	1.6	ATZ	4930568E12Rik	Cntn5	3120999	Phxr4	127668
chr1	120331257	120332801	1.6	ATZ	Clasp1	Mki67ip	100849	2900060B14Rik	22445

chr5	107350031	107350874	1.6	ATZ	Hfm1	Zfp644	224182	Cdc7	42467
chr5	88406205	88407873	1.6	ATZ	1700066N21Rik	Csn3	44916	Cabs1	603
chr11	74574346	74575455	1.6	ATZ	NA	Pafah1b1	36460	Mettl16	8910
chr13	21530870	21531309	1.6	ATZ	Pgbd1	Zkscan3	36246	Zscan26	2735
chr8	44611514	44612333	1.6	ATZ	Adam26b	Zfp42	219151	Adam26a	41297
chr14	20237394	20237681	1.6	ATZ	NA	Ube2e2	511253	Gm5458	175679
chr9	83137753	83139451	1.6	ATZ	NA	Hmgn3	97539	Lca5	145511
chr16	69877225	69878723	1.6	ATZ	NA	Speer2	13236	Gbe1	435471
chr6	57552754	57553137	1.6	ATZ	Herc6	Ppm1k	67334	Pyurf	81596
chr13	94992671	94993215	1.6	ATZ	Scamp1	Lhfpl2	27307	Gm9776	133489
chr9	43873127	43873502	1.6	ATZ	NA	Thy1	16465	Usp2	1602
chr5	134606521	134606885	1.6	ATZ	Gatsl2	Auts2	1588308	Wbscr16	17043
chr4	22864951	22866296	1.6	ATZ	NA	Pou3f2	449438	Mms22l	1557313
chr7	28759663	28760232	1.6	ATZ	Zfp780b	1700049G17Rik	45214	Gm4636	9258
chr14	8949459	8950279	1.6	ATZ	Pxk	Rpp14	25099	Pdhb	48226
chr5	74431394	74432548	1.6	ATZ	Usp46	Spata18	360671	Dancr	56560
chr1	44230172	44232146	1.6	ATZ	Ercc5	Bivm	28556	Mettl21e	28769
chr16	52830168	52831144	1.6	ATZ	NA	4930404A05Rik	14311	Zpld1	2394144
chr1	120332946	120334062	1.6	ATZ	Clasp1	Mki67ip	102538	2900060B14Rik	21184
chr8	10252032	10253526	1.6	ATZ	Myo16	Tnfsf13b	216033	B930025P03Rik	616896
chr17	45163975	45164490	1.6	ATZ	Supt3	4930564C03Rik	146141	Cdc5l	364349
chr19	49162662	49163903	1.6	ATZ	NA	Sorcs3	282667	Sorcs1	1053888
chr8	46680457	46682056	1.6	ATZ	Sorbs2	Tlr3	184564	D330022K07Rik	126623
chr1	166393413	166394153	1.6	ATZ	NA	Atp1b1	4927	Dpt	332710
chr10	87824865	87826154	1.6	ATZ	NA	Dram1	5045	Gnptab	16003
chr5	120774856	120777230	1.6	ATZ	NA	Rbm19	125876	Lhx5	104665
chr14	108037820	108038686	1.6	ATZ	NA	1700128A07Rik	1152119	Slitrk1	1270520
chr4	41812954	41813176	1.6	cont	Ccl21b Ccl21c G	Ccl27a	91905	Gm13298	23608
chr6	82626168	82626993	1.6	ATZ	NA	Pole4	23309	Hk2	48028
chr8	90696788	90698048	1.6	ATZ	NA	Heatr3	946	Papd5	25064
chr14	39058166	39059289	1.6	ATZ	NA	Ghitm	1109658	Nrg3	122848
chr6	12368862	12370122	1.6	ATZ	Thsd7a	Gm6578	309282	Tmem106b	649637
chr14	68721030	68722113	1.6	ATZ	NA	Nefl	15236	Nefm	15489
chr10	66648327	66649130	1.6	ATZ	Jmjd1c	Reep3	88591	Nrbf2	80307
chrX	43644518	43646823	1.6	ATZ	4930515L19Rik	1110059M19Rik	1198551	Actrt1	35361
chr14	38419541	38420588	1.6	ATZ	NA	Ghitm	471033	Nrg3	761549
chr8	9130192	9132700	1.6	ATZ	4933430N04Rik	Arglu1	439655	4930453L07Rik	11969
chr16	5371790	5372387	1.6	ATZ	NA	Fam86	115741	Rbfox1	512499
chr2	150953111	150953355	1.6	ATZ	NA	Gm14151	23566	4930442J19Rik	174420
chr1	53026378	53028680	1.6	ATZ	NA	1700019D03Rik	16694	Mstn	89827
chr12	52682405	52682696	1.6	ATZ	NA	Scfd1	131322	Coch	11632
chr10	74480164	74481999	1.6	ATZ	Rtdr1	Gnaz	1542	Rab36	17835
chr11	97717320	97718508	1.6	ATZ	Fbxo47	B230217C12Rik	12963	Plxdc1	66043
chr9	41693469	41696705	1.6	ATZ	NA	2610203C20Rik	292899	Sorl1	79867
chr10	87826393	87828103	1.6	ATZ	NA	Dram1	6573	Gnptab	14054
chr18	68632270	68632851	1.6	ATZ	NA	Mc2r	43296	4930546C10Rik	416290
chr3	152605501	152607236	1.6	ATZ	St6galnac5	Pigk	153524	St6galnac3	258237
chr13	104613543	104614517	1.6	ATZ	Erbb2ip	Srek1	58881	Nln	199002
chr7	66399861	66400855	1.6	ATZ	NA	Atp10a	315065	Ube3a	83265
chr13	118030868	118031963	1.6	ATZ	Emb	Gm6416	106177	Hcn1	359164
chr1	36949934	36950875	1.6	ATZ	Tmem131	Zap70	110273	Cnga3	325231
chr4	9843506	9845081	1.6	ATZ	4930448K20Rik	Gdf6	54014	1700123O12Rik	590097

chr15	19323881	19325737	1.6	ATZ	NA	Cdh10	379892	Acot10	1269232
chr11	48153968	48154521	1.6	ATZ	NA	Sgcd	961164	Gnb2l1	459341
chr6	20918577	20920432	1.6	ATZ	NA	Ankrd7	2088993	Kcnd2	245677
chr1	75328604	75329190	1.6	ATZ	NA	Dnpep	14392	Des	27729
chr10	114385442	114386711	1.6	ATZ	NA	Trhde	147016	Tph2	128986
chr5	31652113	31653775	1.6	ATZ	NA	Gckr	22438	Zfp512	101034
chr2	21990672	21991106	1.6	ATZ	NA	Gpr158	238503	Myo3a	158024
chr1	139862747	139865400	1.6	ATZ	Mir181a-1 Mir181b-1	Nr5a2	1005722	Ptprc	94036
chr3	6135400	6135726	1.6	ATZ	NA	Pex2	559152	1700008P02Rik	479687
chr9	54029137	54032749	1.6	ATZ	Cyp19a1	Tnfaip8l3	112919	1700104A03Rik	83638
chr2	41148892	41149310	1.6	ATZ	Lrp1b Mir684-1	Ppp6c	2067034	Kynu	2261539
chr10	18505419	18505982	1.6	ATZ	Gm4922	D10Bwg1379e	41855	4930444F02Rik	11123
chr14	69945546	69947016	1.6	cont	NA	Synb	33190	Gm16677	748
chr1	98209645	98211343	1.6	ATZ	NA	1700063A18Rik	291310	4930533P14Rik	297291
chr11	22240512	22242795	1.6	ATZ	NA	Ehbp1	53714	Tmem17	169491
chr12	82477513	82478815	1.6	ATZ	Gm4787	Slc8a3	43346	Adam4	41721
chr3	112453294	112453967	1.6	ATZ	NA	Gm6602	568375	Amy2b	496607
chr17	21196609	21196852	1.6	ATZ	NA	Zfp160	30789	Vmn1r234	168938
chr2	180448493	180449788	1.6	ATZ	Gid8	Dido1	3789	Slc17a9	10256
chr8	68941269	68941744	1.6	ATZ	March1	BC030870	1287833	Tma16	58501
chr5	43353841	43354778	1.6	ATZ	NA	Bod1l	1118287	Gm7854	188147
chr5	99931672	99932974	1.6	ATZ	NA	Rasgef1b	249726	A930011G23Rik	224789
chr12	97900104	97901520	1.6	ATZ	NA	1700019M22Rik	614672	Galc	1538990
chr3	31587088	31588012	1.6	ATZ	NA	Slc7a14	377847	Kcnmb2	213613
chr17	55786056	55787477	1.6	ATZ	NA	Vmn2r118	22086	Pot1b	3846
chr1	56075363	56076486	1.6	ATZ	NA	9130227L01Rik	46421	Hsfy2	616407
chr12	16678833	16679902	1.6	ATZ	Greb1	Ntsr2	11791	E2f6	137869
chr1	37551357	37551905	1.6	ATZ	Mgat4a	4930594C11Rik	5907	2010300C02Rik	116616
chr2	14440276	14440729	1.6	ATZ	NA	Slc39a12	23672	Cacnb2	85204
chr7	90858891	90859704	1.6	ATZ	Il16	Stard5	68053	1700026D08Rik	64423
chr10	113304846	113305486	1.6	ATZ	NA	Atxn7l3b	938764	Trhde	530391
chr1	178683009	178684995	1.6	ATZ	Cep170	Pld5	477566	Mir350	17461
chrX	73630557	73631572	1.6	ATZ	NA	Gm4937	117545	4930428D18Rik	7117
chr9	18201386	18202465	1.6	ATZ	NA	Naalad2	11014	Ubtfl1	6397
chr13	63535647	63537044	1.6	ATZ	NA	Fancc	2594	Ptch1	75797
chr18	37096578	37096895	1.6	ATZ	Pcdha1	Gm6756	14717	Pcdha2	1964
chr2	169211582	169212945	1.6	ATZ	NA	1700017J07Rik	407176	Tshz2	246201
chr11	52001398	52001672	1.6	ATZ	NA	Olfr1372-ps1	29989	Olfr1371	24882
chr14	30174836	30175968	1.6	ATZ	Cacna2d3	Lrtm1	328008	Selk	605598
chr7	112561108	112563264	1.6	ATZ	NA	Cnga4	3856	Cckbr	11070
chr13	59662098	59662989	1.6	ATZ	NA	Agtpbp1	3418	A230056J06Rik	18548
chr14	40893330	40894320	1.6	ATZ	NA	1700109I08Rik	394749	Sh2d4b	734837
chr10	86003246	86004212	1.6	ATZ	NA	Syn3	41605	1810014B01Rik	144060
chr10	18379500	18380762	1.6	ATZ	D10Bwg1379e	Hebp2	113618	Gm4922	118771
chr5	145803958	145804158	1.6	ATZ	1700018F24Rik	Gm4871	10325	Arpc1a	40580
chr2	65104578	65106311	1.6	ATZ	Mir684-1	Cobll1	27895	Slc38a11	48379
chr13	34831407	34832525	1.6	ATZ	Fam50b	Pxdc1	86857	Prpf4b	134838
chr9	79086113	79086819	1.6	ATZ	NA	Cd109	522046	Gm10635	205025
chr18	9099216	9100050	1.6	ATZ	NA	Wac	1170191	Fzd8	112804
chr2	91409914	91412149	1.6	ATZ	Ckap5	Lrp4	55856	Snord67	24089
chr16	89218841	89220386	1.6	ATZ	NA	Krtap20-2	12202	Krtap21-1	182886

chr5	99936364	99941799	1.6	ATZ	NA	Rasgef1b	254418	A930011G23Rik	215964
chr5	122963541	122965172	1.6	ATZ	NA	Atp2a2	11307	Ift81	35041
chr12	101394420	101396363	1.6	ATZ	Nrde2	Psmc1	32846	Gm10433	29815
chr9	67271644	67272791	1.6	ATZ	Tln2	Mir190	187111	C2cd4b	334453
chr2	14438803	14440215	1.6	ATZ	NA	Slc39a12	22199	Cacnb2	85718
chr7	59270117	59273916	1.6	ATZ	1700015G11Rik	Svip	8729	Luzp2	2816699
chrX	6646464	6648080	1.6	ATZ	Akap4	Ccnb3	27719	Clcn5	87458
chr6	85851209	85852966	1.6	ATZ	1700019G17Rik	Cml2	32078	Cml1	7182
chr1	47001556	47001892	1.6	ATZ	NA	Slc39a10	91202	Tmeff2	3982475
chr4	153950493	153951283	1.6	ATZ	Prdm16	Arhgef16	276489	5930403L14Rik	53517
chr18	52805133	52808718	1.6	ATZ	1700034E13Rik	Zfp474	5649	Gykl1	44615
chr11	73009115	73009904	1.6	ATZ	Ctns	Tax1bp3	13567	Shpk	3080
chrX	108305850	108307063	1.6	ATZ	Cylc1	Pou3f4	294961	Rps6ka6	194738
chr1	136444842	136447126	1.6	ATZ	NA	4931440L10Rik	1243	Kdm5b	9629
chr11	52401493	52402567	1.6	ATZ	NA	9530068E07Rik	179268	Fstl4	175641
chr6	124321395	124323904	1.6	ATZ	NA	1700013D24Rik	14291	Pex5	22930
chr2	180468358	180468938	1.6	ATZ	Slc17a9	Gid8	15136	Bhlhe23	40148
chr4	12338454	12340968	1.6	ATZ	NA	Fam92a	239292	Triqk	493016
chr12	52511919	52512887	1.6	ATZ	Scfd1	G2e3	33946	Coch	181441
chr13	111358642	111360026	1.6	ATZ	NA	Plk2	167591	3110015C05Rik	641651
chr6	49058374	49062140	1.6	ATZ	Igf2bp3	Malsu1	23658	Tra2a	131780
chr1	61896744	61898503	1.6	ATZ	Pard3b	9530026F06Rik	443996	Nrp2	851388
chr12	55595247	55596187	1.6	ATZ	NA	Gm7550	102942	Sptssa	150174
chr1	44135221	44137021	1.6	ATZ	NA	Mettl21c	58370	Tex30	6441
chr16	9036426	9036980	1.6	ATZ	NA	1810013L24Rik	177409	Grin2a	540823
chr4	88083525	88085293	1.6	ATZ	Ptplad2	Focad	26610	Ifnb1	82636
chr11	113033560	113034397	1.6	ATZ	2610035D17Rik	Sox9	384489	4732490B19Rik	30232
chr4	114058845	114062625	1.6	ATZ	NA	Skint11	141212	Trabd2b	16704
chr15	46453727	46455632	1.6	ATZ	4930548G14Rik	4930523O13Rik	126002	Csmd3	956552
chr17	88042884	88046827	1.6	ATZ	Epcam	Calm2	196609	Msh2	25070
chr10	42536242	42538212	1.6	ATZ	Sec63	Ostm1	113974	Scml4	42106
chr8	48029942	48030864	1.6	cont	NA	Irf2	97130	Enpp6	41415
chr11	54342222	54345178	1.6	ATZ	Rapgef6	Fnip1	10479	Cdc42se2	185739
chr5	88407937	88410039	1.6	ATZ	1700066N21Rik	Csn3	46648	Smr3a	21511
chr11	19781164	19782366	1.6	ATZ	NA	4933406G16Rik	805421	Spred2	42079
chr12	55829169	55829992	1.6	ATZ	NA	Eapp	32317	Snx6	17352
chr16	77352432	77353915	1.6	ATZ	2810055G20Rik	Usp25	235407	Mir99a	245266
chr17	92270629	92271539	1.6	ATZ	NA	Nrxn1	778487	Adcyap1	1327223
chr1	3863106	3864563	1.6	ATZ	NA	Xkr4	201527	Rp1	416364
chr11	19776886	19780305	1.6	ATZ	NA	4933406G16Rik	801143	Spred2	44140
chr5	65669826	65671345	1.6	ATZ	Rfc1	Wdr19	18172	Klb	68305
chr3	40420089	40420643	1.6	ATZ	1700017G19Rik	Fat4	1509184	Intu	19046
chr5	146165768	146167402	1.6	ATZ	NA	Cyp3a57	14387	Cyp3a16	29776
chr15	63929790	63931060	1.6	ATZ	Asap1	Fam49b	37780	Adcy8	599537
chr5	5535576	5537390	1.6	ATZ	NA	Cldn12	20600	Gtpbp10	67
chr1	163826944	163828064	1.6	ATZ	NA	Suco	20152	4930558K02Rik	44156
chr3	127560829	127562685	1.6	ATZ	NA	Ap1ar	20419	5730508B09Rik	9921
chr17	53336301	53336496	1.6	ATZ	NA	Kcnh8	214971	Efhb	201718
chr17	89006628	89008251	1.6	ATZ	NA	Ppp1r21	18921	Ston1	17644
chr9	106527235	106528105	1.6	ATZ	NA	Iqcf4	53937	Iqcf6	808
chr16	80855671	80858113	1.6	ATZ	NA	Tmprss15	1764329	Ncam2	342829
chr13	5914743	5916150	1.6	ATZ	NA	Klf6	45104	Pitrm1	631253

chr4	39072668	39074341	1.6	ATZ	NA	Lingo2	2173891	1700009N14Rik	322985
chr16	70224317	70225132	1.6	ATZ	NA	Speer2	360328	Gbe1	89062
chr15	34063900	34064479	1.6	ATZ	Mtdh	Tspyl5	446262	Laptm4b	103302
chr14	44303828	44304113	1.6	ATZ	NA	Gm10375	118150	Ear1	92318
chr17	56799050	56800042	1.6	ATZ	Catsperd	Lonp1	32724	Ranbp3	12606
chr5	146165101	146165570	1.6	ATZ	NA	Cyp3a57	13720	Cyp3a16	31608
chr14	109328532	109329494	1.6	ATZ	NA	Slitrk1	15076	Slitrk6	1818306
chr18	33092226	33094945	1.6	ATZ	NA	Wdr36	66152	Camk4	3750
chr1	80443685	80444454	1.6	ATZ	1700016L21Rik	Cul3	106680	Dock10	53194
chr14	88085314	88086646	1.6	ATZ	NA	Tdrd3	139999	Pcdh20	777908
chr14	67794248	67797039	1.6	ATZ	NA	Ppp2r2a	102940	Ebf2	55090
chr8	86386142	86387026	1.6	cont	NA	Cd97	120932	Lphn1	36971
chr2	131259274	131261015	1.6	ATZ	NA	Rnf24	80646	Smox	56583
chr10	33311882	33312689	1.6	ATZ	Clvs2	Trdn	115367	Gm4794	173541
chr1	166231125	166234481	1.6	ATZ	Blzf1	4930455F23Rik	13147	Nme7	3325
chr10	18022229	18023433	1.6	ATZ	NA	Ccdc28a	67442	Nhsl1	104048
chr14	39709772	39712153	1.6	ATZ	4930529F24Rik	Ghitm	1761264	1700109I08Rik	756798
chr14	41880946	41883064	1.6	ATZ	Dydc2	Fam213a	53882	Dydc1	3136
chr2	57117256	57119105	1.6	ATZ	Gpd2 Mir684-1	4930555B11Rik	76588	Galnt5	731461
chr2	163936298	163936894	1.6	ATZ	Stk4	Tomm34	39460	Kcns1	52461
chr17	8358047	8358274	1.6	ATZ	NA	Rnaset2b	17350	Fgfr1op	109
chr5	27519210	27520168	1.6	ATZ	Dpp6	4930584F24Rik	699356	Speer4b	302181
chr5	39992854	39995206	1.6	ATZ	4930513D17Rik	Clnk	724922	Hs3st1	9968
chr4	31107987	31109437	1.6	ATZ	NA	4930556G01Rik	325093	Map3k7	941645
chr17	91256505	91257806	1.6	ATZ	Nrxn1	Gm4719	1472172	4930480K15Rik	2703
chr11	110013985	110017553	1.6	ATZ	Abca9	Abca8a	56734	Abca6	20583
chr4	147749117	147752825	1.6	ATZ	NA	Ptchd2	87043	Ubiad1	55781
chr8	24370007	24370808	1.6	ATZ	NA	Golga7	2455	Sfrp1	151166
chr10	112163896	112165736	1.6	ATZ	1700010J16Rik	Kcnc2	260536	Atxn7l3b	196748
chr12	118256153	118256847	1.6	ATZ	Ptprn2	Ncapg2	554149	Gm10421	126149
chr7	137450670	137453377	1.6	ATZ	NA	Fgfr2	40348	Ate1	81631
chr18	29601900	29602366	1.6	ATZ	4930527G23Rik	4930474G06Rik	445223	Pik3c3	830184
chr6	111535597	111536845	1.6	ATZ	NA	Grm7	18373	1700054K19Rik	623857
chr5	103776481	103777357	1.6	ATZ	NA	4930429D17Rik	48433	Ptpn13	76854
chr1	177627574	177631706	1.6	ATZ	Wdr64	Opn3	4853	Exo1	179203
chr11	58042466	58043713	1.6	ATZ	NA	Irgm2	6181	Zfp692	76858
chr13	3600200	3600695	1.6	ATZ	BC016423	Gdi2	34693	Asb13	32583
chr18	12475508	12477454	1.6	ATZ	NA	Ankrd29	11279	Lama3	15079
chr8	80149017	80149879	1.6	ATZ	1700092C02Rik	Tmem184c	14465	Ednra	37049
chr19	43462819	43464289	1.6	ATZ	Hpse2	Hps1	608353	Cnnm1	50637
chr11	34407916	34408617	1.6	ATZ	Dock2	Fam196b	85276	Spdl1	214070
chr3	68844906	68846485	1.6	ATZ	Trim59	Smc4	6361	Kpna4	29658
chr13	20296341	20297626	1.6	ATZ	Elmo1	Gpr141	380215	Aoah	588362
chr15	92316941	92318248	1.6	ATZ	Pdzrn4	Cntn1	144543	Gxylt1	751925
chr1	92478872	92479891	1.6	ATZ	NA	Cxcr7	366575	Cops8	20109
chr11	30473676	30474837	1.6	ATZ	Acyp2	4930505A04Rik	101847	Psme4	196938
chr18	74019038	74020484	1.6	ATZ	Mro	Me2	43992	Mapk4	67657
chr14	60109522	60110640	1.6	ATZ	Cab39l	Setdb2	49808	Cdadc1	67585
chr16	85780740	85781736	1.6	ATZ	NA	Cyyr1	230122	Adamts1	12337
chr4	101133226	101134681	1.6	ATZ	Ak4	0610043K17Rik	61436	Dnajc6	34572
chr18	67939131	67940566	1.6	ATZ	4930549G23Rik	Ptpn2	54856	Cep192	19195
chr15	44918078	44918502	1.6	ATZ	NA	A930017M01Rik	201789	Kcnv1	19328

chr15	6750434	6751473	1.6	ATZ	NA	Rictor	34	Osmr	12104
chr4	129528398	129529623	1.6	ATZ	NA	Ptp4a2	1151	E330017L17Rik	53847
chr5	17855699	17855942	1.6	ATZ	Gnai1	Gnat3	330213	Magi2	876922
chr19	34350846	34351468	1.6	ATZ	NA	Acta2	20983	Fas	13681
chr1	179718036	179719146	1.6	ATZ	Adss	1700016C15Rik	34600	Gm16432	202420
chr9	65743924	65745966	1.6	ATZ	2810417H13Rik	Zfp609	68553	Csnk1g1	10851
chr8	93683948	93686295	1.6	ATZ	NA	Aktip	24553	Rpgrip1l	54634
chr17	33527002	33527816	1.6	ATZ	Zfp101	Zfp81	31179	Actl9	42028
chr16	78693995	78696231	1.6	ATZ	NA	D16Ertd472e	117062	Chodl	234962
chr6	116626598	116627142	1.6	cont	NA	Rassf4	2744	Tmem72	15601
chr4	146772593	146772876	1.6	ATZ	Gm13152	Znf41-ps	215869	Gm13154	154510
chr18	62736124	62737834	1.6	ATZ	Spink10	Fbxo38	27727	Spink7	14233
chr17	72086420	72086985	1.6	ATZ	NA	Fam179a	7411	BC027072	5910
chr15	92151946	92153755	1.6	ATZ	Cntn1	Muc19	386960	Pdzrn4	73486
chr3	40828205	40830119	1.6	ATZ	NA	Larp1b	46490	Pgrmc2	40129
chr6	120887740	120890151	1.6	ATZ	Mical3	Bid	20902	Pex26	243534
chr18	3308528	3310750	1.6	ATZ	Crem	Vmn1r238	185116	Gm6225	25664
chr17	70271616	70273437	1.6	ATZ	NA	A330050F15Rik	433043	Dlgap1	500695
chr1	23913723	23918723	1.6	ATZ	Smap1	B3gat2	59026	1110058L19Rik	84055
chr6	100709918	100711585	1.6	ATZ	Gxylt2	Shq1	88767	Ppp4r2	72047
chr17	54090728	54092206	1.6	ATZ	NA	Sult1c2	105445	Sult1c1	8734
chr7	134759875	134760736	1.6	ATZ	NA	Zfp629	1928	Bcl7c	87756
chr7	91899350	91899655	1.6	ATZ	2610206C17Rik	Zfand6	71489	Olfr291	102426
chr10	41752122	41753478	1.6	ATZ	NA	Armc2	13934	Foxo3	152114
chr9	103106302	103106930	1.6	ATZ	NA	Srprb	1907	Trf	4276
chr10	66651827	66652297	1.6	ATZ	Jmjd1c	Reep3	92091	Nrbf2	77140
chr17	51018412	51019064	1.6	ATZ	Tbc1d5	Plcl2	190593	C330011F03Rik	545470
chr1	179738679	179738958	1.6	ATZ	NA	Adss	12039	Gm16432	182608
chr13	99632264	99634279	1.6	ATZ	Tnpo1	Fcho2	46860	1700024P04Rik	119766
chr1	73068851	73070239	1.6	ATZ	1700027A15Rik	Tnp1	6378	Pinc	367720
chr6	101148078	101149384	1.6	ATZ	Pdzrn3	Ppp4r2	329367	Gm9871	574859
chr6	57428432	57428732	1.6	ATZ	NA	Vmn1r20	45836	Ppm1k	27764
chr17	47805248	47806057	1.6	ATZ	Usp49	Med20	43881	Tomm6	17537
chr1	121831354	121833990	1.5	ATZ	Gm101	Tmem177	21609	Sctr	69567
chr5	22196284	22197114	1.5	ATZ	NA	Orc5	140135	A930003O13Rik	47615
chr3	35560908	35562973	1.5	ATZ	Gm6639	Gm3143	945324	Atp11b	90087
chr15	94721788	94722841	1.5	ATZ	Tmem117	Twf1	301533	LOC100043315	205212
chr17	66124831	66127833	1.5	ATZ	NA	Rab31	2739	Ppp4r1	4862
chrY	1425238	1426447	1.5	ATZ	Zfy2	Usp9y	629013	Gm16501 Gm6(	428562
chr6	119827101	119827825	1.5	ATZ	NA	3110021A11Rik	28067	Rad52	24891
chr6	42963463	42964676	1.5	ATZ	NA	Olfr444	57032	Olfr441	101067
chr1	43443259	43444640	1.5	ATZ	NA	Fhl2	222453	Nck2	57956
chr15	32997644	33001375	1.5	ATZ	NA	Sdc2	33168	Cpq	11509
chr1	102147573	102150583	1.5	ATZ	Cntnap5b	Slco6d1	1741616	Gm20268	901762
chr13	56697328	56699001	1.5	ATZ	NA	Lect2	47429	Tgfbi	11963
chr1	73070386	73075293	1.5	ATZ	1700027A15Rik	Tnp1	7913	Pinc	362666
chr6	75365232	75366425	1.5	ATZ	NA	4931417E11Rik	1945571	Ctnna2	1465206
chr11	43163888	43168383	1.5	ATZ	NA	Atp10b	88101	Mir146	19516
chr3	126764873	126765134	1.5	ATZ	NA	Ank2	63503	Larp7	474498
chr3	87800724	87801493	1.5	cont	Bcan	Nes	16351	Hapln2	24179
chr14	51576098	51577107	1.5	ATZ	Pnp2	Pnp	3011	Rnase10	50319
chr16	91114834	91115707	1.5	ATZ	NA	4932438H23Rik	45444	Olig2	110088

chr8	68942164	68943361	1.5	ATZ	March1	BC030870	1288728	Tma16	56884
chr9	99758152	99759659	1.5	ATZ	NA	Cldn18	140466	Sox14	14866
chr6	58533179	58534481	1.5	ATZ	NA	Vmn1r31	110307	Abcg2	12185
chr5	15219200	15219659	1.5	ATZ	Speer7-ps1	4930572O03Rik	56323	4930519H02Rik	150087
chr14	52314452	52314908	1.5	ATZ	NA	Gm5622	31824	Gm5800	16411
chr3	6138058	6141087	1.5	ATZ	NA	Pex2	561810	1700008P02Rik	474326
chr5	31786214	31789316	1.5	ATZ	4930548H24Rik	Zfp512	2088	Gpn1	7818
chr12	52529990	52531701	1.5	ATZ	Scfd1	G2e3	52017	Coch	162627
chr7	36323530	36324115	1.5	ATZ	NA	Tdrd12	767	Nudt19	8089
chr16	5935830	5936772	1.5	ATZ	Rbfox1	Fam86	679781	1700123O21Rik	38908
chr8	69369695	69370290	1.5	ATZ	NA	Npy1r	138998	Naf1	13826
chr14	68399464	68401609	1.5	ATZ	Dock5	Gnrh1	31971	Nefl	300332
chr6	106724132	106724867	1.5	ATZ	Trnt1	Il5ra	25101	Crbn	3372
chr17	38061240	38061501	1.5	ATZ	Olfr128	Olfr127	19776	Olfr761	27495
chr16	85381208	85384669	1.5	ATZ	NA	App	207256	Cyyr1	71821
chr11	85007133	85009470	1.5	ATZ	Appbp2	1700125H20Rik	12477	D630032N06Rik	39198
chr7	134601623	134602216	1.5	ATZ	NA	Zfp689	8951	Prr14	12912
chr4	154497273	154499951	1.5	ATZ	Morn1	Rer1	36867	Ski	28233
chr12	32516108	32517129	1.5	ATZ	Cog5	Gpr22	117317	Hbp1	94191
chr2	17480553	17481838	1.5	ATZ	Nebl	4930515L03Rik	545200	H2afb1	436211
chr18	8065854	8066373	1.5	ATZ	NA	Wac	136829	Fzd8	1146481
chr4	155357810	155358889	1.5	ATZ	NA	Fam132a	17072	B3galt6	4686
chr6	30463134	30465316	1.5	ATZ	1700025E21Rik	Tmem209	3428	Cpa2	26326
chr14	67754173	67756356	1.5	ATZ	NA	Ppp2r2a	62865	Ebf2	95773
chr3	153662210	153664921	1.5	ATZ	Slc44a5	Acadm	54603	Lhx8	304337
chr13	46704017	46704754	1.5	ATZ	Cap2	Rbm24	177549	C78339	60137
chr18	31483746	31484530	1.5	ATZ	NA	Rit2	6964	Syt4	112932
chr8	106126477	106127420	1.5	ATZ	NA	Cdh11	817466	4933400L20Rik	279
chr8	74381173	74381501	1.5	ATZ	NA	Fcho1	131593	Zfp709	24466
chr4	129334160	129335268	1.5	ATZ	Kpna6	Txlna	15851	Tmem39b	18331
chr14	25184485	25184992	1.5	ATZ	NA	E330034G19Rik	55304	Polr3a	82924
chr13	113925845	113927272	1.5	ATZ	NA	Gzma	34656	Gzmk	34810
chr2	57116472	57117169	1.5	ATZ	Gpd2 Mir684-1	4930555B11Rik	75804	Galnt5	733397
chr12	93515509	93517327	1.5	ATZ	NA	Sel1l	427912	Flrt2	3413109
chr13	43096173	43097453	1.5	ATZ	Phactr1	Edn1	692815	Tbc1d7	149656
chr14	8722714	8725405	1.5	ATZ	Flnb	Gm3558	321634	Dnase1l3	72299
chr6	76850004	76854371	1.5	ATZ	Ctnna2	4931417E11Rik	3430343	Lrrtm1	338340
chr8	80292881	80293418	1.5	ATZ	NA	Ednra	44530	Ttc29	443823
chr2	78106181	78107423	1.5	ATZ	Gm14461 Mir68	Cwc22	321771	Ube2e3	601781
chr17	29618846	29621276	1.5	ATZ	NA	Fgd2	102366	Pim1	6714
chr2	113547277	113549881	1.5	ATZ	4930533B01Rik	Tmco5b	409930	Grem1	38951
chr15	20654789	20655616	1.5	ATZ	NA	Acot10	58284	Cdh12	385591
chr10	70329020	70330549	1.5	ATZ	NA	D630013N20Rik	210309	Bicc1	57299
chr1	89300936	89304641	1.5	ATZ	Gigyf2	Kcnj13	9632	3110079O15Rik	62198
chr2	130927816	130931127	1.5	ATZ	NA	Siglec1	15315	Hspa12b	22021
chr12	106760848	106762225	1.5	ATZ	NA	D430019H16Rik	29543	Bdkrb2	39157
chr3	154857634	154859958	1.5	ATZ	NA	Lrriq3	392	9330178D15Rik	779360
chr16	85379583	85381134	1.5	ATZ	NA	App	205631	Cyyr1	75356
chr3	84779320	84781469	1.5	ATZ	Fbxw7	Dear1	9818	1700036G14Rik	339972
chr1	102150647	102151865	1.5	ATZ	Cntnap5b	Slco6d1	1744690	Gm20268	900480
chr2	66008407	66009677	1.5	ATZ	Mir684-1	Galnt3	45557	Ttc21b	12709
chr17	53401273	53404024	1.5	ATZ	NA	Kcnh8	279943	Efhb	134190

chr17	7165663	7165883	1.5	ATZ	NA	Tagap1	158	Rnaset2b	17326
chr2	68404302	68404660	1.5	ATZ	Mir684-1	Stk39	94264	4933409G03Rik	15810
chr13	14410813	14411407	1.5	ATZ	Hecw1	Arid4b	118943	Mrpl32	291161
chr1	100315716	100316649	1.5	ATZ	NA	1810006J02Rik	274484	Slco6d1	1052
chr8	27193113	27195987	1.5	ATZ	Hook3	Fnta	67040	Rnf170	33865
chr7	123138634	123140994	1.5	ATZ	Sox6	A730082K24Rik	1011153	1700003G18Rik	84282
chr4	25897828	25898830	1.5	ATZ	NA	Fut9	170678	Manea	352823
chr1	26743638	26745273	1.5	ATZ	4931408C20Rik	Bai3	857086	Gm597	2087694
chr12	15732682	15733128	1.5	ATZ	NA	4930448C13Rik	675487	Trib2	65405
chr2	131987236	131988410	1.5	ATZ	NA	Slc23a2	16392	Tmem230	76818
chr5	109047928	109049391	1.5	ATZ	Gak	Cplx1	68882	Tmem175	9438
chr17	22635875	22636124	1.5	ATZ	NA	Zfp946	42219	Vmn2r111	48784
chr10	74740993	74741477	1.5	ATZ	Specc1l	Bcr	93325	Adora2a	38211
chr1	72267282	72268971	1.5	ATZ	NA	Mreg	8401	Pecr	36776
chr6	136578670	136579593	1.5	ATZ	Plbd1	Atf7ip	22770	Gucy2c	66212
chr10	67781298	67784930	1.5	ATZ	4930545H06Rik	Arid5b	39824	1700040L02Rik	108774
chr13	112274352	112277228	1.5	ATZ	Gpbp1	Actbl2	226395	Mier3	199158
chr6	8470054	8471999	1.5	ATZ	Glcci1	A430035B10Rik	10893	Ica1	108528
chr14	76974728	76975162	1.5	ATZ	NA	Serp2	18032	1700108F19Rik	102213
chr1	21894797	21895501	1.5	ATZ	Kcnq5	Khdc1b	518332	Rims1	383002
chr15	13521348	13524140	1.5	ATZ	NA	Cdh6	417954	Cdh9	3183716
chr9	18580228	18580918	1.5	ATZ	NA	Olfr24	20151	Olfr828	38880
chr13	46835783	46837319	1.5	ATZ	NA	Nup153	12565	Kif13a	7138
chr2	180469146	180470126	1.5	ATZ	Slc17a9	Gid8	15924	Bhlhe23	38960
chr11	18732535	18734592	1.5	ATZ	NA	Etaa1	878657	Meis1	45839
chr11	37558643	37561887	1.5	ATZ	NA	Tenm2	800898	Gm12130	743163
chr3	38907597	38910533	1.5	ATZ	Fat4	Ankrd50	523859	1700017G19Rik	1493254
chr4	114236968	114237616	1.5	ATZ	Trabd2b	Skint11	319335	Gm12830	256709
chr4	34702464	34705282	1.5	ATZ	Gm136	1700003M02Rik	25009	Smim8	10639
chr6	24323214	24324245	1.5	ATZ	NA	Slc13a1	205122	Iqub	70620
chr2	164541288	164541817	1.5	cont	Spint5	Spint4	13340	Wfdc3	14909
chr2	151257312	151257829	1.5	ATZ	NA	4930442J19Rik	115573	4921509C19Rik	38449
chr5	120256540	120259103	1.5	ATZ	NA	Gm16065	115313	Tbx5	25569
chr18	80825641	80827436	1.5	ATZ	Nfatc1	Gm2176	84356	Atp9b	103444
chr13	21827166	21827656	1.5	cont	Hist1h4j	Hist1h2bm	12819	Hist1h4k	14407
chr4	135545636	135547261	1.5	ATZ	NA	Pithd1	2477	Tceb3	12024
chr15	81235364	81239268	1.5	ATZ	Dnajb7 Xpnpep3	St13	5240	Rbx1	57478
chr11	114285677	114289258	1.5	ATZ	4932435O22Rik	1700092K14Rik	225164	Rpl38	240599
chr9	54113404	54116365	1.5	ATZ	NA	Cyp19a1	72155	1700104A03Rik	22
chr3	105816172	105818608	1.5	ATZ	Pifo	Ovgp1	25831	Chia	97692
chr3	108546456	108547816	1.5	ATZ	Aknad1	Gpsm2	21239	Stxbp3a	48282
chr2	65874093	65877791	1.5	ATZ	Mir684-1	Csrnp3	4490	Galnt3	43032
chr2	56362235	56363002	1.5	ATZ	Mir684-1	Kcnj3	914299	Nr4a2	596635
chr1	10045163	10049274	1.5	ATZ	Cspp1	Cops5	17184	Arfgef1	78314
chr2	105316048	105317564	1.5	ATZ	NA	Rcn1	76572	Pax6os1	58673
chr5	6412462	6414852	1.5	ATZ	NA	Steap1	663145	Zfp804b	354178
chr15	46154816	46157698	1.5	ATZ	1700022A22Rik	Kcnv1	1208336	4930523O13Rik	109714
chr18	70266548	70267698	1.5	ATZ	Rab27b	Ccdc68	137410	Dynap	132385
chr6	29229398	29232014	1.5	ATZ	Fam71f2	Hilpda	3949	Fam71f1	37126
chr4	134467453	134468059	1.5	ATZ	Tmem50a	Rhd	15366	D4Wsu53e	11481
chr4	35850671	35851768	1.5	ATZ	Lingo2	3110043O21Rik	677542	Mir876	740638
chr14	97785706	97786879	1.5	ATZ	NA	Klhl1	867453	Dach1	399187

chr3	120984323	120987281	1.5	ATZ	NA	Tmem56	18089	Alg14	7454
chr5	115762182	115764218	1.5	ATZ	NA	Dynll1	11183	Srsf9	12968
chr5	120408002	120411227	1.5	ATZ	NA	Tbx5	72775	Rbm19	155295
chr6	100711658	100712565	1.5	ATZ	Gxylt2	Shq1	90507	Ppp4r2	71067
chr12	92206866	92209910	1.5	ATZ	NA	Dio2	229988	Cep128	27022
chr16	78223331	78225037	1.5	ATZ	NA	Mir125b-2	576743	E330011O21Rik	26071
chr1	78736788	78737776	1.5	ATZ	NA	Acsl3	32470	Kcne4	75748
chr15	77258312	77258667	1.5	ATZ	Apol7b	Apol9a	16802	Apol10a	48810
chrX	166216007	166217706	1.5	ATZ	4933400A11Rik	Hccs	457732	G530011O06Rik	195269
chr14	27512438	27513933	1.5	ATZ	NA	Pde12	23106	Asb14	193857
chr7	145745184	145748582	1.5	ATZ	NA	Tcerg1l	155771	Mapk1ip1	278919
chr12	84039774	84041685	1.5	ATZ	1700085C21Rik	Sipa1l1	487003	Dpf3	273053
chr2	160683158	160684959	1.5	ATZ	Zhx3	Plcg1	81662	Lpin3	21447
chr5	135085197	135086545	1.5	ATZ	Lat2	Rfc2	10999	Eif4h	9201
chr6	17680702	17682125	1.5	ATZ	NA	Capza2	64166	St7	17091
chr9	15168131	15170997	1.5	ATZ	NA	5830418K08Rik	5899	BC017612	138942
chr1	53028782	53030459	1.5	ATZ	NA	1700019D03Rik	19098	Mstn	88048
chr12	100344742	100345934	1.5	ATZ	NA	Ttc8	123294	4930474N09Rik	53553
chr6	76698124	76699913	1.5	ATZ	NA	4931417E11Rik	3278463	Ctnna2	131718
chr15	47152348	47154164	1.5	ATZ	NA	4930548G14Rik	680944	Csmd3	258020
chr9	104058978	104060633	1.5	ATZ	Dnajc13	Acad11	29002	Acpp	129937
chr1	103098235	103098939	1.5	ATZ	NA	Gm20268	26456	Cdh20	3566457
chr10	51508798	51510190	1.5	ATZ	NA	4933411G06Rik	31798	Vgll2	232302
chr13	65133951	65136740	1.5	ATZ	Spata31d1c	Spata31	109448	Hiatl1	29598
chr11	43709544	43711144	1.5	ATZ	NA	Adra1b	59710	Il12b	502421
chr2	91169357	91170906	1.5	ATZ	1110051M20Rik	Arfgap2	51829	Lrp4	126782
chr16	38143268	38145388	1.5	ATZ	Gsk3b	BC031361	53922	Nr1i2	103047
chr5	118859163	118860589	1.5	ATZ	NA	2410131K14Rik	146040	Med13l	150139
chr2	169213184	169216002	1.5	ATZ	NA	1700017J07Rik	408778	Tshz2	243144
chr1	179719227	179721422	1.5	ATZ	Adss	1700016C15Rik	35791	Gm16432	200144
chr14	44325776	44326406	1.5	ATZ	NA	Gm10375	140098	Ear1	70025
chr15	33478522	33481973	1.5	ATZ	Cpq	1700084J12Rik	142828	4930592A05Rik	41663
chr16	77351769	77352351	1.5	ATZ	2810055G20Rik	Usp25	234744	Mir99a	246830
chr6	83769113	83771153	1.5	ATZ	Paip2b	Nagk	16563	Zfml	93194
chr16	73073139	73074918	1.5	ATZ	NA	Robo1	26794	Robo2	817633
chr18	54414837	54416982	1.5	ATZ	NA	Csnk1g3	299499	9430076G02Rik	164967
chr3	79434015	79434823	1.5	ATZ	4930579G24Rik	Etfdh	1326	Rxfp1	13815
chr16	76121327	76124571	1.5	ATZ	4930578N18Rik	Samsn1	211816	Nrip1	166536
chr14	120073185	120075326	1.5	ATZ	Hs6st3	Uggt2	574529	1700006F04Rik	73123
chr7	39236281	39237164	1.5	ATZ	NA	Pop4	179914	Gm5591	65994
chr7	102701387	102703215	1.5	ATZ	NA	Fam181b	2471156	Tenm4	655932
chr1	83269421	83271713	1.5	ATZ	Sphkap	Wdr69	62274	Pid1	761155
chr1	188748980	188752013	1.5	ATZ	NA	Rrp15	175743	D1Pas1	39282
chr11	97399858	97402736	1.5	ATZ	Srcin1	Arhgap23	36144	2410003L11Rik	57089
chr14	20236574	20237182	1.5	ATZ	NA	Ube2e2	510433	Gm5458	176178
chr1	46723370	46723870	1.5	ATZ	NA	Dnahc7b	292975	Slc39a10	140519
chr4	62254381	62257090	1.5	ATZ	Rgs3	4933430I17Rik	45354	Zfp618	369518
chr18	64435266	64437459	1.5	ATZ	1700091E21Rik	Wdr7	285853	Onecut2	62559
chr15	36210487	36211270	1.5	ATZ	Rnf19a	Spag1	45555	Ankrd46	196153
chr1	96073418	96074300	1.5	ATZ	NA	Pdcd1	124288	4930440C22Rik	619719
chr1	72688411	72689129	1.5	ATZ	NA	Smarcal1	5047	Ankar	425
chr13	43516964	43518660	1.5	ATZ	Ranbp9	Nol7	18737	Ccdc90a	115115

chr5	60793819	60795369	1.5	ATZ	NA	4933402J10Rik	416578	G6pd2	1404713
chr17	7830162	7830648	1.5	ATZ	Gm1604b Rnaset2b	Gm9992	240508	Fndc1	100785
chr11	53167212	53167639	1.5	ATZ	Aff4	Zcchc10	20409	Leap2	68044
chr3	88270656	88271562	1.5	ATZ	NA	Sema4a	5552	Lmna	13509
chr11	14076142	14079835	1.5	ATZ	NA	Cobl	1711179	1700046C09Rik	380504
chr12	71969215	71970467	1.5	ATZ	Frmd6	Tmx1	400604	Actr10	68377
chr1	121794857	121796989	1.5	ATZ	NA	Ptpn4	61209	Tmem177	7489
chr15	79152586	79153554	1.5	ATZ	Pla2g6	Baiap2l2	36647	Maff	24554
chr8	121428540	121430759	1.5	ATZ	Cdh13	Mphosph6	1102711	Hsbp1	437679
chr1	32603342	32603906	1.5	ATZ	Gm5415 Khdrbs	Gm9839	25498	Prim2	906750
chr18	74845541	74846827	1.5	ATZ	Myo5b	1700120E14Rik	154763	Scarna17	91221
chr1	107049585	107050999	1.5	ATZ	NA	Cdh20	157527	Rnf152	122495
chr1	39906633	39910311	1.5	ATZ	NA	1700066B17Rik	2458	Map4k4	47447
chr16	90078445	90080971	1.5	ATZ	NA	Tiam1	103501	Gm10789	61511
chr3	8746016	8747542	1.5	ATZ	NA	Hey1	78978	Mrps28	54604
chr1	163088279	163091187	1.5	ATZ	4930469G21Rik	Klhl20	26669	Prdx6	79056
chr17	77713382	77713839	1.5	ATZ	NA	Gm4710	1425003	Crim1	885749
chr17	54506433	54507634	1.5	ATZ	1700025K24Rik	Slc5a7	68074	St6gal2	1077381
chr7	6339562	6340122	1.5	ATZ	Zfp28	Zfp78	4247	Gm16532	27764
chr3	84764802	84765719	1.5	ATZ	Fbxw7	Tmem154	256305	Dear1	3259
chr2	91167204	91169196	1.5	ATZ	1110051M20Rik	Arfgap2	49676	Lrp4	128492
chr8	104222329	104223498	1.5	ATZ	NA	Cdh8	2281972	Cdh11	933397
chr14	119133727	119134083	1.5	ATZ	NA	Abcc4	28286	Cldn10	53010
chr5	147611949	147614607	1.5	ATZ	NA	Usp12	5417	Rpl21	29859
chr14	124386474	124387683	1.5	ATZ	1700024B18Rik	Itgbl1	13173	NA	NA
chr8	109981247	109983205	1.5	ATZ	Wwp2	Nob1	32309	Mir140	91939
chr15	13083611	13086538	1.5	ATZ	Cdh6	Drosha	218565	Cdh9	3621318
chr17	38039794	38040356	1.5	ATZ	NA	Olfr126	51296	Olfr127	137
chr6	60671499	60672765	1.5	ATZ	NA	A530053G22Rik	317798	Snca	8802
chr8	107949864	107954258	1.5	ATZ	Lrrc36	Kctd19	12477	Tppp3	37134
chr4	10724045	10725306	1.5	ATZ	1700123O12Rik	4930448K20Rik	879501	2610301B20Rik	76339
chrX	133510187	133510761	1.5	ATZ	Tmsb15b1 Tmsb	Tmsb15b2	17669	Slc25a53	4894
chr7	31946862	31947971	1.5	ATZ	Gm10640	Gramd1a	10793	Scgb1b2	127567
chr3	91944426	91947263	1.5	ATZ	Lelp1	Prr9	16557	Sprr2a1 Sprr2a2	72494
chr14	123112957	123114393	1.5	ATZ	Pcca	Zic2	233407	Ggact	175689
chr7	26344210	26345678	1.5	ATZ	4732471J01Rik	Ceacam2	19187	Gm7092	53962
chr6	24682670	24685027	1.5	ATZ	Hyal6	Wasl	67675	Hyal4	13340
chr1	57444308	57445162	1.5	ATZ	Tyw5	1700066M21Rik	2042	9430016H08Rik	18230
chr13	86282937	86283492	1.5	ATZ	NA	Cox7c	96537	Edil3	2677585
chr7	48923080	48925378	1.5	ATZ	Vmn2r-ps54	Zfp788	16178	Vmn2r58	166873
chr1	166121859	166124896	1.5	ATZ	F5	Gm16548	39511	Slc19a2	54281
chr14	76836063	76838028	1.5	ATZ	Tsc22d1	Nufip1	298877	4930444M15Rik	76337
chr9	118295966	118296323	1.5	ATZ	NA	Cmc1	236652	4933432G23Rik	42634
chr15	27785275	27787545	1.5	ATZ	Trio	Fam105a	173978	Dnahc5	345976
chr2	113186581	113188402	1.5	ATZ	Fmn1	Tmco5b	49234	4930533B01Rik	342808
chr12	88070977	88071638	1.5	ATZ	NA	Angel1	3567	Gm6772	3681
chr17	29621499	29625073	1.5	ATZ	NA	Fgd2	105019	Pim1	2917
chr8	37237878	37239551	1.5	ATZ	NA	D8Ertd82e	27037	Lonrf1	39567
chr2	148644721	148647637	1.5	ATZ	Cst13	Cst8	13401	Cst9	13246
chr1	142551975	142553020	1.5	ATZ	4930590L20Rik	Kcnt2	45137	Cdc73	2901609
chr14	52925226	52925605	1.5	ATZ	NA	Mettl3	427	Sall2	5247

chr5	88410379	88410605	1.5	ATZ	Cabs1	1700066N21Rik	2003	Smr3a	20945
chr16	17346471	17347225	1.5	ATZ	Pi4ka	Serpind1	2806	Snap29	58868
chr2	17891682	17892155	1.5	ATZ	NA	Nebl	238987	H2afb1	25894
chr4	125513890	125515501	1.5	ATZ	NA	Grik3	122473	2610028E06Rik	49594
chr3	151838439	151839913	1.5	ATZ	NA	Gipc2	9575	Dnajb4	7006
chr17	71663816	71665047	1.5	ATZ	NA	Emilin2	3511	Gm4566	17494
chr3	6619475	6621077	1.5	ATZ	1700008P02Rik	Pex2	1043227	Pkia	745527
chr1	50162420	50163313	1.5	ATZ	NA	Slc39a10	3252066	Tmeff2	821054
chr10	63282280	63284908	1.5	ATZ	Ctnna3	Dnajc12	410692	Lrrtm3	106337
chr4	19931320	19933069	1.5	ATZ	4930480G23Rik	Atp6v0d2	81607	Ttpa	2506
chr5	22819825	22821040	1.5	ATZ	NA	Lhfpl3	38410	5031425E22Rik	116586
chr8	74968274	74970035	1.5	ATZ	1700030K09Rik	Calr3	597	Cherp	14347
chr17	7830926	7832450	1.5	ATZ	Gm1604b Rnase	Gm9992	241272	Fndc1	98983
chr14	48955198	48957426	1.5	ATZ	NA	Peli2	74640	6720456H20Rik	108601
chr3	34638608	34639558	1.5	ATZ	NA	Gm3143	23024	Gm6639	856516
chr12	88112547	88113375	1.5	ATZ	NA	Gm6772	7802	Irf2bpl	108278
chr10	62709337	62710692	1.5	ATZ	Herc4	Mypn	42637	Sirt1	71061
chr4	103331071	103333167	1.5	ATZ	NA	Gm12718	166278	Dab1	706998

Supplemental file 2. Differential H3K4me3 peaks associated with DSBs

Chromosome	Start	End	Fold_Change	H3K4me3_Accumulated	H3K4me3_Within_genes	H3K4me3_nearest_upstream_gene	Distance_to_nearest_upstream_gene	H3K4me3_nearest_downstream_gene	Distance_to_nearest_downstream_gene
chr13	120278317	120279418	4.1	ATZ	Gm7120	3110070M22Rik	1126	NA	NA
chr8	74543719	74544319	2.2	ATZ	NA	Cyp4f18	10194	Olfr372	37240
chr10	92038881	92039318	2.1	ATZ	NA	Gm20757	200763	Nedd1	108172
chr12	46228758	46229166	2.0	ATZ	NA	Stxbp6	53288	Nova1	1566338
chr14	123339870	123340244	2.0	ATZ	Tmtc4	Ggact	27483	AA536875	227481
chr12	47689674	47689940	1.9	ATZ	NA	Stxbp6	1514204	Nova1	105564
chr1	30910083	30910617	1.9	ATZ	Phf3	Gm597	2072986	Ptp4a1	86531
chr1	174105806	174106269	1.8	ATZ	Dcaf8	Pex19	39178	Pea15a	20591
chr14	37739850	37740196	1.8	ATZ	Ccser2	4930474N05Rik	829809	Rgr	110480
chr7	53090957	53091451	1.8	ATZ	Grin2d	Grwd1	4798	Kdelr1	36759
chr3	61022256	61022818	1.8	ATZ	NA	P2ry1	209355	Rap2b	145611
chr14	55298531	55299105	1.8	ATZ	Acin1	4930579G18Rik	24594	1700123O20Rik	5903
chr1	68983601	68983891	1.7	ATZ	Erbb4	Cps1	1706579	Ikzf2	593893
chr14	69947094	69948831	1.7	Cont	Gm16677	Synb	34738	Entpd4	6377
chr1	165736065	165736909	1.7	ATZ	Kifap3	Mettl11b	80702	Scyl3	123286
chr14	119711630	119713140	1.7	ATZ	Hs6st3	Uggt2	212974	1700006F04Rik	435309
chr7	121631675	121632201	1.7	ATZ	Pde3b	4933406I18Rik	73000	Cyp2r1	61481
chr16	96682408	96683439	1.7	ATZ	NA	Itgb2l	17187	Pcp4	5774
chr2	35040527	35041713	1.7	ATZ	Rab14	Cep110	6185	Gsn	70166
chr4	94976976	94977630	1.7	ATZ	NA	Jun	258063	Fggy	246568
chr1	58613144	58613581	1.7	ATZ	Fam126b	Orc2	51191	Ndufb3	29862
chr1	130430361	130430647	1.7	ATZ	NA	Dars	116368	Cxcr4	54129
chr11	117519434	117520027	1.7	ATZ	Tnrc6c	2900041M22Rik	44703	Tmc6	107272
chr14	87560483	87560755	1.6	ATZ	NA	Diap3	19562	Tdrd3	255635
chr13	3598530	3600130	1.6	ATZ	BC016423	Gdi2	33023	Asb13	33148
chr6	58798485	58799408	1.6	ATZ	Herc3	Abcg2	156040	Nap1l5	55819
chr18	49526007	49526544	1.6	ATZ	NA	G630055G22Rik	1258373	1700044K03Rik	156399
chr4	96197763	96198380	1.6	ATZ	Cyp2j6	Cyp2j8	23686	Cyp2j9	36740
chr19	60615205	60616654	1.6	ATZ	Cacul1	Prlhr	70998	Nanos1	215236
chr19	24457474	24458303	1.6	ATZ	Pip5k1b	Fxn	102398	Fam122a	91966
chr19	43218118	43218916	1.6	ATZ	Hpse2	Hps1	363652	Cnnm1	296010
chr1	157540616	157541159	1.6	ATZ	NA	Acbd6	6253	Lhx4	7665
chr13	32360875	32361606	1.6	ATZ	Gmds	Foxc1	458371	Mylk4	431090
chr3	88353557	88354454	1.6	ATZ	Lamtor2	Rab25	1356	Ubqln4	3184
chr7	104792205	104794646	1.6	ATZ	Rsf1	Aamdc	64198	Clns1a	50521
chr4	48463079	48463756	1.6	ATZ	Tex10	Invs	18254	Msantd3	89062
chr3	36909884	36911272	1.6	ATZ	4932438A13Rik	Trpc3	320795	Adad1	51306
chr10	51679917	51681035	1.6	ATZ	NA	4933411G06Rik	202917	Vgll2	61457
chr15	85204494	85205197	1.6	ATZ	Atxn10	Fbln1	87770	7530416G11Rik	117969
chr17	91164415	91164943	1.6	ATZ	Nrxn1	Gm4719	1380082	4930480K15Rik	95566
chr6	122368081	122368616	1.6	ATZ	NA	Phc1	77836	Rimklb	35011
chr14	106883569	106887643	1.6	ATZ	1700128A07Rik	Trim52	348490	Slitrk1	2421563
chr7	125360206	125361504	1.6	ATZ	Smg1	Arl6ip1	87067	4930583K01Rik	25917

chr2	17015210	17017125	1.6	ATZ	NA	4930515L03Rik	79857	Nebl	251236
chr3	119346166	119347447	1.6	ATZ	NA	Dpyd	210330	Ptbp2	74213
chr5	128202311	128202802	1.6	ATZ	NA	Glt1d1	14421	Tmem132d	61059
chr2	37992137	37994382	1.6	ATZ	Dennd1a Mir684-1	Crb2	337514	Lhx2	212446
chr4	106906182	106906704	1.6	ATZ	Lrrc42	Ldlrad1	15663	Hspb11	19835
chr5	115516929	115517695	1.6	ATZ	Sppl3	Hnf1a	95858	Gm13826	35236
chr1	4598241	4599621	1.6	ATZ	NA	Sox17	111747	Mrpl15	163658
chr11	11387352	11388461	1.6	ATZ	NA	Zpbp	24930	4930415F15Rik	808
chr11	31404267	31404772	1.6	ATZ	NA	Stc2	134206	Bod1	160378
chr12	65955679	65956531	1.6	ATZ	NA	Fscb	379794	Gm527	62367
chr14	37715518	37715988	1.6	ATZ	Ccser2	4930474N05Rik	805477	Rgr	134688
chr9	96983753	96984106	1.6	ATZ	Slc25a36	Spsb4	64979	Trim42	265875
chr13	81118403	81120800	1.6	ATZ	9330111N05 Rik	Arrdc3	83100	Gpr98	113267
chr10	75310686	75311903	1.6	ATZ	NA	Gstt2	13060	Mif	10195
chr1	46971615	46972035	1.6	ATZ	NA	Slc39a10	61261	Tmeff2	4012332
chr2	113956184	113956781	1.6	ATZ	Aqr	C130080G10Rik	68946	Zfp770	62416
chr1	101756912	101757942	1.5	ATZ	Cntnap5b	Slco6d1	1350955	Gm20268	1294403
chr2	139514137	139514801	1.5	ATZ	Ism1	Sptlc3	50727	Tasp1	144415
chr1	68007813	68008575	1.5	ATZ	NA	Cps1	730791	Erbb4	77965
chr2	126752438	126754410	1.5	ATZ	Sppl2a	Trpm7	50441	Ap4e1	80037
chr2	168258992	168260119	1.5	ATZ	NA	Kcng1	164161	Nfatc2	41791
chr6	37729328	37730661	1.5	ATZ	NA	Akr1d1	210513	Trim24	90150
chr6	110210664	110212053	1.5	ATZ	NA	Edem1	1401314	Grm7	383539
chr2	102574656	102575731	1.5	ATZ	Slc1a2	Pamr1	91459	Cd44	75569
chr16	31410287	31411120	1.5	ATZ	NA	Apod	95605	Bdh1	11263
chr6	7512317	7513712	1.5	ATZ	Tac1	Acn9	523097	Asns	111459
chr10	38839161	38842529	1.5	ATZ	Fam229b	Lama4	9167	Tube1	11300
chr1	89335132	89335915	1.5	ATZ	Gigyf2	Kcnj13	43828	3110079O15Rik	30924
chr4	53538058	53538582	1.5	ATZ	Slc44a1	AI427809	254954	Fsd1l	105761
chr6	17682592	17683371	1.5	ATZ	NA	Capza2	66056	St7	15845
chr9	41219401	41220623	1.5	ATZ	3110039I08Ri k	1700063D05Rik	193691	Mir100	118885
chr17	66182699	66183548	1.5	ATZ	Ppp4r1	Rab31	60607	Ralbp1	14221
chr2	143637641	143638377	1.5	ATZ	Pcsk2	Gm12360	22719	Bfsp1	13887
chr9	43996174	43997501	1.5	ATZ	Cbl	Mcam	45365	Ccdc153	51259
chr5	38734483	38736468	1.5	ATZ	4930487D11R ik	Drd5	20934	Slc2a9	4044
chr15	65130901	65132233	1.5	ATZ	NA	Adcy8	377043	Efr3a	486370
chr1	37210611	37213217	1.5	ATZ	NA	Tmem131	214239	Cnga3	62889
chr1	140863788	140864774	1.5	ATZ	Dennd1b	2310009B15Rik	110357	Crb1	230057
chr2	157875633	157876207	1.5	ATZ	Rprd1b	Tti1	40537	2010009K17Rik	19893
chr1	194762231	194763370	1.5	ATZ	Syt14	Sertad4	80285	Diexf	167227
chr6	116207533	116208349	1.5	ATZ	Fam21	Tmcc1	64141	Zfand4	5904
chrX	166434738	166436029	1.5	Cont	NA	Mid1	6009	NA	NA
chr1	121665481	121665873	1.5	ATZ	Ptpn4	Epb4.1l5	119904	Tmem177	138605
chr7	96427465	96427844	1.5	ATZ	Tmem135	Rab38	787383	Fzd4	125032

supplemental file 3. Differential peaks located nearby ncRNAs

Chr	start	end	FC	Overlap_lncRNA	upstream_lncRNA	Downstream_lncRNA
chr2	80475903	80476675	1.64	NA	kinorura	ENSMUSG00000087061
chr5	39697186	39697578	1.71	NA	foflu	yohuse
chr6	116207533	116208349	1.51	NA	D6Wsu116e	Anubl1
chr7	36323530	36324115	1.54	NA	2410004F06Rik	ENSMUSG00000084801
chr11	19781164	19782366	1.58	NA	rakuhe	LOC665868
chr12	102287546	102288352	1.80	NA	Smek1	fupu
chr13	112274352	112277228	1.53	NA	ENSMUSG00000086201	Gpbp1
chr17	53336301	53336496	1.57	NA	LOC668506	yukuya
chr4	41812954	41813176	1.63	NA	Ccl19-ps1	RP23-304I1.3
chr1	73068851	73070239	1.55	NA	weesny	1700027A15Rik
chr1	136444842	136447126	1.59	NA	dusnu	namiyora
chr1	188748980	188752013	1.52	NA	ENSMUSG00000090488	LOC666832
chr10	87824865	87826154	1.63	NA	1200002N14Rik	shyty
chr15	81235364	81239268	1.53	Xpnpep3 yatemi	vular	Rbx1
chr17	6493079	6493317	2.24	NA	neriya	LOC100040531
chr19	43462819	43464289	1.56	NA	LOC545291	tureru
chr6	101148078	101149384	1.55	ENSMUSG00000091352 tisisa	tasisa	ratusu
chr8	106126477	106127420	1.54	NA	Gm20073	4933400L20Rik
chr8	109981247	109983205	1.51	NA	hotetu	choywor
chr9	18201386	18202465	1.60	NA	husone	Naalad2
chr9	54113404	54116365	1.53	NA	Elmod1	Gldnos
chr1	30910083	30910617	1.88	Phf3	tahurora	slasnar
chr1	61896744	61898503	1.58	huhorora	hahorora	nersnu
chr1	72688411	72689129	1.51	sohorora	skeysney	Rpl37a
chr1	142551975	142553020	1.50	4930590L20Rik	foysnu	ENSMUSG00000089418
chr1	163826944	163828064	1.57	tonarora	AI848100	2810442N19Rik
chr1	178683009	178684995	1.60	Cep170 yuhayura mahayura sanirora	tonirora	ENSMUSG00000084749
chr10	66651827	66652297	1.55	Jmjd1c	toytey	wutey
chr11	52001398	52001672	1.60	ENSMUSG00000087101	ENSMUSG00000092028	nihoho
chr11	72423270	72423799	1.65	Ube2g1	zoyrar	wasoho
chr12	84039774	84041685	1.52	1700085C21Rik	wanahi	plorpoy
chr13	20296341	20297626	1.56	Elmo1	chorny	foymoy
chr13	76408015	76408452	1.64	nehumo	AK129128	kanamo
chr13	99632264	99634279	1.55	Tnpo1	wawnoy	tirahi
chr14	8722714	8725405	1.54	1700110I01Rik	kuromu	Flnb
chr14	8949459	8950279	1.63	1700110I01Rik	Rpp14	Pdhb
chr14	20236574	20237182	1.52	1700110I01Rik	smorler	LOC100039441
chr14	20237394	20237681	1.64	1700110I01Rik	smorler	LOC100039441

chr14	70008610	70009670	2.11	ENSMUSG00000085092 romimu	Entpd4	ENSMUSG000000852 43
chr14	76836063	76838028	1.51	Gm19597	kawley	4930444M15Rik
chr14	119133727	119134083	1.51	rimemu	1700044C05Rik	snumey
chr15	20654789	20655616	1.54	sahama	LOC100040265	nasuma
chr15	46453727	46455632	1.58	4930548G14Rik	wahuma	skerla
chr15	79152586	79153554	1.51	Pla2g6	Gm10863	Csnk1e
chr16	96682408	96683439	1.69	sawjey	Dscr2	hitiyu
chr17	6429271	6429489	1.77	LOC547127 ENSMUSG00000091795	huriya	Dynlt1
chr17	6476411	6476645	1.82	ENSMUSG00000091795	LOC547127	neriya
chr17	54506433	54507634	1.51	1700025K24Rik	tukuya	duger
chr17	70271616	70273437	1.55	tokaro	ENSMUSG000000885 81	soroyu
chr17	71663816	71665047	1.50	ENSMUSG00000085916 kakaro	sokaro	Ndc80
chr18	29601900	29602366	1.56	4930578E11Rik 4930527G23Rik	namure	numure
chr18	45861158	45861734	1.69	tumere	tururo	gygu
chr18	64435266	64437459	1.52	St8sia3os	temore	Fech
chr2	14438803	14440215	1.59	ENSMUSG00000084901 susirara	Stam	hiharira
chr2	78106181	78107423	1.54	Gm14461	LOC667920	OTTMUSG000000167 03
chr2	171059519	171060824	1.82	ENSMUSG00000084829 ENSMUSG00000086613	ENSMUSG000000848 18	1700028P15Rik
chr3	40420089	40420643	1.58	1700017G19Rik	fapley	Intu
chr3	84183136	84183927	1.74	4930565D16Rik	flerpler	Arfip1
chr3	91944426	91947263	1.51	A030004J04Rik	kleypler	LOC619984
chr4	154497273	154499951	1.54	Morn1	Pank4	Ski
chr5	22196284	22197114	1.55	LOC668232 ENSMUSG00000087558	horeki	sleeflu
chr5	38734483	38736468	1.52	4930487D11Rik	yahuse	Wdr1
chr5	99931672	99932974	1.61	A930011G23Rik	kenuse	rinese
chr6	8470054	8471999	1.53	Gm16039	A430035B10Rik	soresi
chr6	40877973	40878730	1.70	1700074P13Rik	totesi	LOC100039790
chr6	127749306	127749457	1.65	fushey	Parp11	soybloy
chr6	135609075	135610578	1.74	susesa	sisesa	geyshey
chr7	31946862	31947971	1.51	Gm10640	ENSMUSG000000854 58	borchee
chr7	31948073	31948979	1.72	Gm10640	ENSMUSG000000854 58	borchee
chr7	123138634	123140994	1.54	sumite	simite	EG668479and1110004 F10Rik
chr8	27193113	27195987	1.54	1700047A11Rik	Hook3	kehati
chr8	37237878	37239551	1.50	hoseta	huseta	6430573F11Rik
chr8	46680457	46682056	1.63	LOC100861925 ENSMUSG00000084954 seseta	mituti	ENSMUSG000000886 29
chr8	60007688	60008195	1.64	Galnt7	husota	Gm2921
chr8	66190792	66191168	2.23	tisota	BC013672	tusota

chr9	41219401	41220623	1.52	3110039I08Rik	kemone	D230004N17Rik
chr9	104058978	104060633	1.52	korvaw	DnaJ.0	nahone
chr9	118295966	118296323	1.51	parvaw	povaw	4933432G23Rik
chr1	174105806	174106269	1.82	Wdr42a	sumuyura	ninirora
chr10	66648327	66649130	1.62	Jmjd1c	toytey	wutey
chr10	69070299	69070962	2.06	namonu	tiruni	Ank3
chr10	87826393	87828103	1.62	shyty	1200002N14Rik	ENSMUSG00000091432
chr11	101983975	101985159	1.74	LOC666411	mosey	snorar
chr13	104613543	104614517	1.62	Erbb2ip	harehi	yohomo
chr13	106679224	106679563	1.91	muhomo	sorohi	bleemoy
chr14	24937827	24938301	1.64	sneeler	heromu	Dlg5
chr14	69953593	69956310	1.81	rumimu Gm16677 Entpd4	1700092C10Rik	remimu
chr14	104397145	104397593	1.79	4930432J09Rik namumu	homumu	moley
chr14	124386474	124387683	1.51	momemu 1700024B18Rik	mememu	neromu
chr15	94721788	94722841	1.55	Tmem117	Irak4	LOC100043315
chr16	73073139	73074918	1.52	kunoyu	sakuyo	nawjey
chr17	22635875	22636124	1.53	susiro	1300003B13Rik	numeya
chr18	74019038	74020484	1.56	perfey	mihare	storgar
chr18	77094514	77095422	1.76	EG664805	tahare	ryfey
chr2	14440276	14440729	1.60	ENSMUSG00000084901 susirara	Stam	hiharira
chr2	156217533	156217700	1.82	2900097C17Rik	LOC669792	nawarira
chr4	19931320	19933069	1.50	Ttpa	ENSMUSG00000086071	ENSMUSG00000090934
chr4	146772593	146772876	1.55	ENSMUSG00000084976 ENSMUSG00000091833 LOC100043100	LOC666581	OTTMUSG00000010671
chr6	6243671	6244169	1.66	ENSMUSG00000093482	Slc25a13	hirusi
chr8	51601691	51602594	1.77	misota	Gm2516	mawwor
chr8	80149017	80149879	1.56	1700092C02Rik	ENSMUSG00000091247	tukata
chr9	77655868	77656207	1.82	fawvaw	farvaw	Ick
chr9	83137753	83139451	1.64	hahene	govaw	murita
chr9	122560789	122561878	1.88	ENSMUSG00000086476 9530059O14Rik	monine	reevaw
chr1	36949934	36950875	1.61	Tmem131	EG623414	rarsny
chr1	53026378	53028680	1.62	1700019D03Rik	sehoyara	seherora
chr1	53028782	53030459	1.52	1700019D03Rik	sehoyara	seherora
chr1	73070386	73075293	1.55	1700027A15Rik	weesny	wersny
chr1	78736788	78737776	1.52	zeysny	yonarora	hekayara
chr1	80443685	80444454	1.57	1700016L21Rik	veesnor	hikiyara
chr1	139862747	139865400	1.61	ratirora	ramoyora	Ptprc
chr10	62709337	62710692	1.50	komanu	suty	rominu
chr10	67781298	67784930	1.53	Gm19746 4930545H06Rik nuruni	LOC100040136	noruni
chr10	112163896	112165736	1.56	tayani	noyani	gleety
chr11	14076142	14079835	1.51	ENSMUSG00000085452	hisohe	1700046C09Rik
chr11	19776886	19780305	1.58	rakuhe	ferrer	LOC665868

chr11	54342222	54345178	1.58	Rapgef6	haniho	Cdc42se2
chr11	113033560	113034397	1.58	2610035D17Rik	nonohe	4732490B19Rik
chr11	114285677	114289258	1.53	4932435O22Rik	ENSMUSG00000075431	swyrar
chr12	92206866	92209910	1.52	ENSMUSG00000092100 tenahi	LOC100039295	ENSMUSG00000084566
chr13	37806834	37809620	1.91	momomo	karmoy	blarno
chr13	56697328	56699001	1.55	ruhimo	AU042651	ENSMUSG00000092091
chr13	59662098	59662989	1.60	A230056J06Rik	hoheha	Mak10
chr13	81118403	81120800	1.56	ENSMUSG00000085266 9330111N05Rik	LOC100043074	LOC218368
chr13	95317580	95319459	1.65	chymoy	nokeha	chomoy
chr13	112277401	112278213	1.66	Gpbp1	regulation of transcription	ENSMUSG00000085634
chr14	15942542	15943519	1.67	1700110I01Rik reyamu	ruyamu	ENSMUSG00000090616
chr14	39709772	39712153	1.57	Nrg3os	heyumu	niyumu
chr14	60109522	60110640	1.56	Cab39l	kumome	Cdadc1
chr14	69945546	69947016	1.61	rumimu Gm16677	1700092C10Rik	Entpd4
chr14	69947094	69948831	1.74	rumimu Gm16677	1700092C10Rik	Entpd4
chr14	106883569	106887643	1.60	1700128A07Rik	mawley	LOC668752
chr15	9083841	9086297	1.70	Skp2	1110020G09Rik	Lmbrd2
chr15	46154816	46157698	1.52	kohima 1700022A22Rik	siyami	wahuma
chr15	77258312	77258667	1.52	nahema	glerker	1700025B11Rik
chr16	52830168	52831144	1.63	LOC667895	4930404A05Rik	niteyo
chr16	76121327	76124571	1.52	4930578N18Rik	Samsn1	Nrip1
chr16	77351769	77352351	1.52	2810055G20Rik	ritayu	yarima
chr16	77352432	77353915	1.58	2810055G20Rik	ritayu	yarima
chr16	78693995	78696231	1.56	4930570E03Rik	4930478L05Rik	yaruma
chr17	6429542	6429778	2.63	LOC547127 ENSMUSG00000091795	huriya	Dynlt1
chr17	6475846	6476233	2.18	ENSMUSG00000091795	LOC547127	neriya
chr17	6489260	6489496	1.93	ENSMUSG00000091795	LOC547127	neriya
chr17	6491637	6491975	2.47	ENSMUSG00000091795 neriya	LOC547127	LOC100040531
chr17	6492231	6492684	2.38	ENSMUSG00000091795 neriya	LOC547127	LOC100040531
chr17	29618846	29621276	1.54	tasuro ENSMUSG00000087551	tisuro	Pim1
chr17	29621499	29625073	1.51	tasuro ENSMUSG00000087551	tisuro	Pim1
chr18	33092226	33094945	1.57	ENSMUSG00000087447	4930455D15Rik	sosere
chr18	67939131	67940566	1.56	4930549G23Rik	B430212C06Rik	ENSMUSG00000092977
chr18	68632270	68632851	1.62	komore	D18Ertd653e	sheegar
chr19	43218118	43218916	1.62	LOC545291	verfar	tireru
chr2	105316048	105317564	1.53	ENSMUSG00000086764	Wt1os	Pax6os1
chr2	113547277	113549881	1.54	4930533B01Rik	dosly	deesly
chr2	150953111	150953355	1.62	4921509C19Rik	ENSMUSG00000085805	ENSMUSG00000087337

chr2	151257312	151257829	1.53	4921509C19Rik ENSMUSG00000087495	kekorara	ENSMUSG000000868 00 4930556L07Rik
chr2	169213184	169216002	1.52	vorsly	ENSMUSG000000853 69	ENSMUSG000000853 50
chr2	180448493	180449788	1.61	2310003C23Rik	Dido1	ENSMUSG000000861 66
chr3	35560908	35562973	1.55	LOC625963	Gm3143	dopley
chr4	10724045	10725306	1.51	1700123O12Rik	homeki	Mir3471-1
chr4	22864951	22866296	1.63	ENSMUSG00000028244	tusoki	komeki
chr4	37196302	37197507	2.02	ENSMUSG00000086026	LOC545600	heruko
chr5	6412462	6414852	1.52	rohise	LOC546854	kasise
chr5	15217817	15218108	6.16	Speer7-ps1	nekoka	4930519H02Rik
chr5	15219200	15219659	1.54	Speer7-ps1	nekoka	4930519H02Rik
chr5	39992854	39995206	1.57	mahuse	ENSMUSG000000878 45	soraso
chr5	88406205	88407873	1.64	1700066N21Rik	2310003L06RikandSmr 1	henuse
chr5	88407937	88410039	1.58	1700066N21Rik	2310003L06RikandSmr 1	henuse
chr6	20918577	20920432	1.61	zersher zeysher	romusi	hemusi
chr6	29229398	29232014	1.52	chusher	snorshoy	chosher
chr6	40878942	40880372	1.65	1700074P13Rik	totesi	LOC100039790
chr6	42963463	42964676	1.55	bleysher	BC011487	matesa
chr6	85851209	85852966	1.59	1700019G17Rik	hosasa	Nat8b
chr7	46570637	46571072	1.67	neyate	1700010N08Rik	skeychee
chr7	134601623	134602216	1.54	B130055M24Rik	fushaw	1700008J07Rik
chr8	9130192	9132700	1.62	4933430N04Rik	nesita	beywor
chr9	13105243	13106913	1.64	hamene 4930568E12Rik	tasane	weevee
chr9	67271644	67272791	1.59	Tln2	hihune	beevaw
chr9	106527235	106528105	1.57	tihone	Pcbp4	ENSMUSG000000883 17
chrX	43644518	43646823	1.62	4930515L19Rik	karda	shadu
chr5	99936364	99941799	1.60	A930011G23Rik	kenuse	rinese
